# The PTPN2/PTPN1 inhibitor ABBV-CLS-484 unleashes potent anti-tumour immunity

**DOI:** 10.1038/s41586-023-06575-7

**Published:** 2023-10-04

**Authors:** Christina K. Baumgartner, Hakimeh Ebrahimi-Nik, Arvin Iracheta-Vellve, Keith M. Hamel, Kira E. Olander, Thomas G. R. Davis, Kathleen A. McGuire, Geoff T. Halvorsen, Omar I. Avila, Chirag H. Patel, Sarah Y. Kim, Ashwin V. Kammula, Audrey J. Muscato, Kyle Halliwill, Prasanthi Geda, Kelly L. Klinge, Zhaoming Xiong, Ryan Duggan, Liang Mu, Mitchell D. Yeary, James C. Patti, Tyler M. Balon, Rebecca Mathew, Carey Backus, Domenick E. Kennedy, Angeline Chen, Kenton Longenecker, Joseph T. Klahn, Cara L. Hrusch, Navasona Krishnan, Charles W. Hutchins, Jax P. Dunning, Marinka Bulic, Payal Tiwari, Kayla J. Colvin, Cun Lan Chuong, Ian C. Kohnle, Matthew G. Rees, Andrew Boghossian, Melissa Ronan, Jennifer A. Roth, Meng-Ju Wu, Juliette S. M. T. Suermondt, Nelson H. Knudsen, Collins K. Cheruiyot, Debattama R. Sen, Gabriel K. Griffin, Todd R. Golub, Nabeel El-Bardeesy, Joshua H. Decker, Yi Yang, Magali Guffroy, Stacey Fossey, Patricia Trusk, Im-Meng Sun, Yue Liu, Wei Qiu, Qi Sun, Marcia N. Paddock, Elliot P. Farney, Mark A. Matulenko, Clay Beauregard, Jennifer M. Frost, Kathleen B. Yates, Philip R. Kym, Robert T. Manguso

**Affiliations:** 1 AbbVie, North Chicago, IL USA; 2 Broad Institute of MIT and Harvard, Cambridge, MA USA; 3 Center for Cancer Research and Department of Medicine, Massachusetts General Hospital, Boston, MA USA; 4 Calico Life Sciences, South San Francisco, CA USA; 5 AbbVie, South San Francisco, CA USA; 6 Dana-Farber Cancer Institute, Boston, MA USA; 7 Present Address: Ohio State University Comprehensive Cancer Center and Pelotonia Institute for Immuno-Oncology, Columbus, OH USA; 8 Present Address: Pfizer, Groton, CT USA; 9 Present Address: Bristol Myers Squibb, Summit, NJ USA; 10 Present Address: Ipsen Biosciences, Cambridge, MA USA; 11 Present Address: Monte Rosa Therapeutics, Boston, MA USA; 12 Present Address: Vir Biotechnology, San Francisco, CA USA

**Keywords:** Tumour immunology, Cancer immunotherapy, Preclinical research

## Abstract

Immune checkpoint blockade is effective for some patients with cancer, but most are refractory to current immunotherapies and new approaches are needed to overcome resistance^1,2^. The protein tyrosine phosphatases PTPN2 and PTPN1 are central regulators of inflammation, and their genetic deletion in either tumour cells or immune cells promotes anti-tumour immunity^3–6^. However, phosphatases are challenging drug targets; in particular, the active site has been considered undruggable. Here we present the discovery and characterization of ABBV-CLS-484 (AC484), a first-in-class, orally bioavailable, potent PTPN2 and PTPN1 active-site inhibitor. AC484 treatment in vitro amplifies the response to interferon and promotes the activation and function of several immune cell subsets. In mouse models of cancer resistant to PD-1 blockade, AC484 monotherapy generates potent anti-tumour immunity. We show that AC484 inflames the tumour microenvironment and promotes natural killer cell and CD8^+^ T cell function by enhancing JAK–STAT signalling and reducing T cell dysfunction. Inhibitors of PTPN2 and PTPN1 offer a promising new strategy for cancer immunotherapy and are currently being evaluated in patients with advanced solid tumours (ClinicalTrials.gov identifier NCT04777994). More broadly, our study shows that small-molecule inhibitors of key intracellular immune regulators can achieve efficacy comparable to or exceeding that of antibody-based immune checkpoint blockade in preclinical models. Finally, to our knowledge, AC484 represents the first active-site phosphatase inhibitor to enter clinical evaluation for cancer immunotherapy and may pave the way for additional therapeutics that target this important class of enzymes.

## Abstract (web summary)

An orally bioavailable small-molecule active-site inhibitor of the phosphatases PTPN2 and PTPN1, ABBV-CLS-484, demonstrates immunotherapeutic efficacy in mouse models of cancer resistant to PD-1 blockade.

## Main

The phosphatase PTPN2 (also known as TC-PTP) and its paralogue PTPN1 (also known as PTP-1B) are negative regulators of several cytokine signalling pathways and T cell receptor (TCR) signalling and therefore act as crucial checkpoints of inflammation^6,7^. PTPN2 and PTPN1 (PTPN2/N1) dampen inflammation by dephosphorylating members of the JAK and STAT families, positioning them as negative regulators of signalling through interferon (IFN), interleukin-2 (IL-2), IL-15 and others^7–11^. PTPN2 also targets proximal TCR signalling molecules such as FYN and LCK, thereby dampening antigen sensitivity in T cells^12^. Loss-of-function (LOF) single-nucleotide polymorphisms in the *PTPN2* locus are associated with several autoimmune diseases, which is probably due to the central role of PTPN2 in the regulation of inflammation^10,13^.

Previous studies have shown that loss of PTPN2 in tumour cells enhances the response to immunotherapy by sensitizing tumours to IFNγ^3^. Additionally, deletion of PTPN2 or PTPN1 enhances the expansion of T cells, the response to IL-2 and the ability to control tumours^4–6,11^. Thus, in contrast to current therapies, a PTPN2/N1-targeted therapy would engage a dual anticancer mechanism by acting directly on tumour cells and increasing anti-tumour activity of immune cells.

**Fig. 1:**
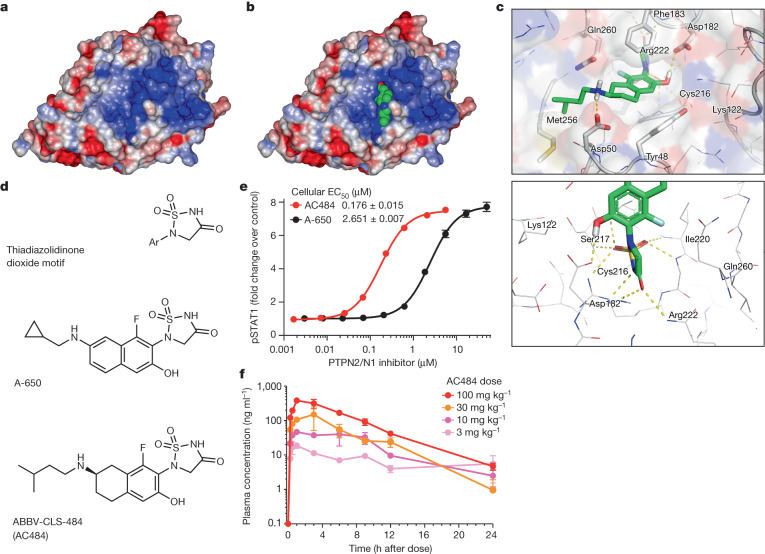
The discovery of AC484, a PTPN2/N1 active-site inhibitor. **a**, Structure of PTPN2, with blue indicating the basicity of the active site (using a pH scale of 0–14, with pH < 7 indicating acidic (red) and pH > 7 basic (blue); pH 7 is neutral (white)). **b**, AC484 (green) in the PTPN2 protein, coloured as in **a**. **c**, Crystal structure of AC484 in the active site of PTPN2. **d**, Structure of PTPN2/N1 inhibitors, including AC484. **e**, Impact of PTPN2/N1 inhibitors on IFNγ-mediated STAT1 phosphorylation in B16 tumour cells (*n* = 10 per inhibitor; mean ± s.e.m.). **f**, AC484 dose-escalating pharmacokinetics in mice at doses of 3, 10, 30 and 100 mg kg^–1^ with once-daily dosing (*n* = 3 per group; mean ± s.e.m.). Source data

The challenge of identifying drug candidates for phosphatase targets is well known in drug discovery. Phosphatases have highly polar active sites that have been described as undruggable^14,15^. Specifically, phosphatase active sites require highly polar inhibitors to drive enzymatic potency (Fig. 1a). Consequently, although PTPN1 inhibitors with potent enzymatic activity (<10 nM) have been identified, reported chemotypes exhibit weak cellular activity (>10 μM) and poor pharmacokinetic properties^16^. Such analogues cannot effectively inhibit the target in cells when dosed in vivo, which makes them unsuitable as therapeutics. Efforts to identify drugs that target phosphatases have focused on allosteric modulators; however, no such allosteric inhibitors have been identified for PTPN2 (ref. ^17^). Owing to the importance of PTPN2/N1 in tumour and immune cells and the high homology of their active sites, we sought to discover a PTPN2/N1 small-molecule inhibitor that targets these two phosphatases.

Here we present the discovery and characterization of AC484, a potent, orally bioavailable small-molecule inhibitor of PTPN2/N1 for cancer therapy.

## Discovery of a dual PTPN2/N1 inhibitor

As loss of either PTPN2 or PTPN1 increased the sensitivity of tumour cells to checkpoint blockade in an in vivo CRISPR screen, and because both phosphatases dampen immune cell activation^3–6,11^, we sought to identify a dual PTPN2/N1 inhibitor to further enhance anti-tumour activity. Significant efforts, including those focused on phosphotyrosine mimetics, have been reported for PTPN1, but did not produce approved drugs^16^. PTPN2 and PTPN1 have highly homologous active sites; therefore we designed molecules that optimized interactions in both PTPN2 and PTPN1 through structure-based drug design (Fig. 1a–c). We focused our design on the optimization of drug-like properties, including low molecular weight (<500 Daltons), good sp^3^ content and low clearance. Structural motifs such as thiadiazolidinone dioxide have been described, although these analogues exhibit modest biochemical and poor cellular potency^16^. Structure-based drug design that optimized interactions near the thiadiazolidinone dioxide ring and the naphthyl side chain led to the discovery of A-650, which demonstrated potent biochemical inhibition of both PTPN2 (half-maximum inhibitory concentration (IC_50_) of 3.9 nM) and PTPN1 (IC_50_ of 2.8 nM) (Fig. 1d and Extended Data Table 1). Although A-650 exhibited moderate cellular activity in an assay for IFNγ-induced phosphorylation of STAT1 (a direct target of both PTPN2 and PTPN1 (ref. ^7^)) in B16F10 mouse melanoma (B16) cells (Fig. 1e, black), poor physiochemical properties precluded its advancement as a drug candidate. Subsequently, partial saturation of the naphthyl core to obtain the corresponding amino-tetralin and optimization of the amine side chain led to the discovery of AC484 (Fig. 1d and Extended Data Table 1). The crystal structure of the PTPN2 protein with AC484 confirmed the formation of several crucial interactions in the active site Cys216 region (Fig. 1b,c and Extended Data Table 2). The highly acidic thiadiazolidinone dioxide moiety made up to nine interactions with residues in this region, including hydrogen bonds with the Cys216, Arg222, Asp182, Ser217 and Ile220 residues (Fig. 1c). We additionally sought interactions proximal to the tetralin ring and designed the isopentyl-amine group to further engage in a hydrogen bond with Asp50 and a hydrophobic interaction with Met256 (Fig. 1c). Owing to the high homology in the active sites, corresponding interactions were also observed in a crystal of AC484 with PTPN1 (data not shown), which resulted in low nanomolar biochemical potency for both PTPN2 (IC_50 _of 1.8 nM) and PTPN1 (IC_50 _of 2.5 nM) (Extended Data Table 1). AC484 is a zwitterionic compound (NH of thiadiazolidinone dioxide p*K*_a_ = 0.9; amine NH p*K*_a_ = 10), and this feature proved pivotal for its observed properties, including the distinct combination of low plasma protein binding (86% fraction unbound in mouse plasma and 50% in human plasma) and low clearance (Extended Data Table 3). AC484 did not exhibit hepatic clearance in vitro or in vivo. Instead, it is cleared through renal and biliary clearance mechanisms. The combination of optimized properties and ligand–target interactions of AC484 drove low nanomolar enzymatic potency and significantly improved cellular potency (STAT1 IC_50_ of 176 nM in B16 cells; Fig. 1e, red). Notably, AC484 also demonstrated linear increases in dose-escalating exposures and good target coverage in mice (Fig. 1f). Thus, oral dosing of AC484 can effectively achieve potent drug levels in vivo. Additionally, in a biochemical phosphatase screen, AC484 demonstrated high selectivity for PTPN2/N1, with 6–8-fold weaker activity on PTPN9 and no detectable activity on SHP-1 or SHP-2 (Extended Data Table 4). Furthermore, wide-range selectivity screens across a diverse panel of phosphatases, kinases and other receptors, including the hERG channel, showed no off-target activity of AC484 (Extended Data Table 5 and Supplementary Tables 1 and 2). AC484 also exhibited low levels in the brain (0.08 unbound mouse brain/plasma ratio) (Extended Data Table 3).

## AC484 sensitizes tumour cells to IFNγ

**Fig. 2:**
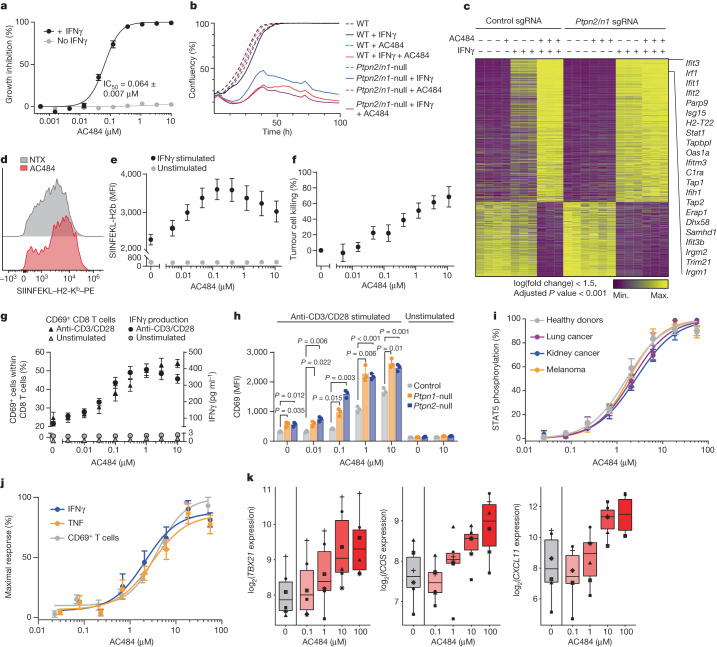
AC484 increases mouse and human tumour cell sensitivity to IFNγ and enhances T cell activation and function in vitro. **a**, Per cent growth inhibition of B16 tumour cells treated with AC484 with or without IFNγ (0.5 ng ml^−1^) (*n* = 5). **b**, In vitro growth curve of control and *Ptpn2/n1*-null B16 cells with or without AC484 (1 µM) and with or without IFNγ (10 ng ml^−1^) (*n* = 3). **c**, Heatmap showing transcriptional response to IFNγ (10 ng ml^−1^) in control or *Ptpn2/n1*-null B16 cells with or without AC484 (10 μM) treatment. **d**,**e**, IFNγ-induced antigen presentation on B16-OVA cells left untreated (NTX) or treated with AC484 (0.1 µM). Representative histograms (**d**) and MFI ± s.e.m. of SIINFEKL–H2-K^b^ (**e**, *n* = 6). **f**, OT-I T cell-mediated B16-OVA tumour cell killing (*n* = 3). **g**, Activation (per cent of CD69-expressing CD8^+^ T cells, *n* = 6) and IFNγ production (*n* = 8) of anti-CD3/CD28-stimulated splenic pan T cells isolated from C57BL/6N mice. **h**, CD69 expression (MFI) of control, *Ptpn2*-null and *Ptpn1*-null mouse CD8^+^ T cells with or without anti-CD3/CD28 stimulation and with or without AC484. **i**–**k**, AC484 pathway engagement and immune activation in human whole blood. **i**, pSTAT5 in whole blood from healthy donors (*n* = 8) and from patients with cancer (*n* = 5 per indication) stimulated with IL-2. **j**, Normalized frequency of CD69-expressing T cells (*n* = 4) and IFNγ and TNF production (*n* = 5) in TCR-stimulated whole blood from healthy donors. **k**, Gene expression for top differentially expressed genes in PBMCs from TCR-stimulated healthy donor whole blood (*n* = 6). The relationship between dose and gene expression is significant (*P* < 0.05) for all genes. Error bars represent the mean ± s.e.m. Source data

**Extended Data Fig. 1:**
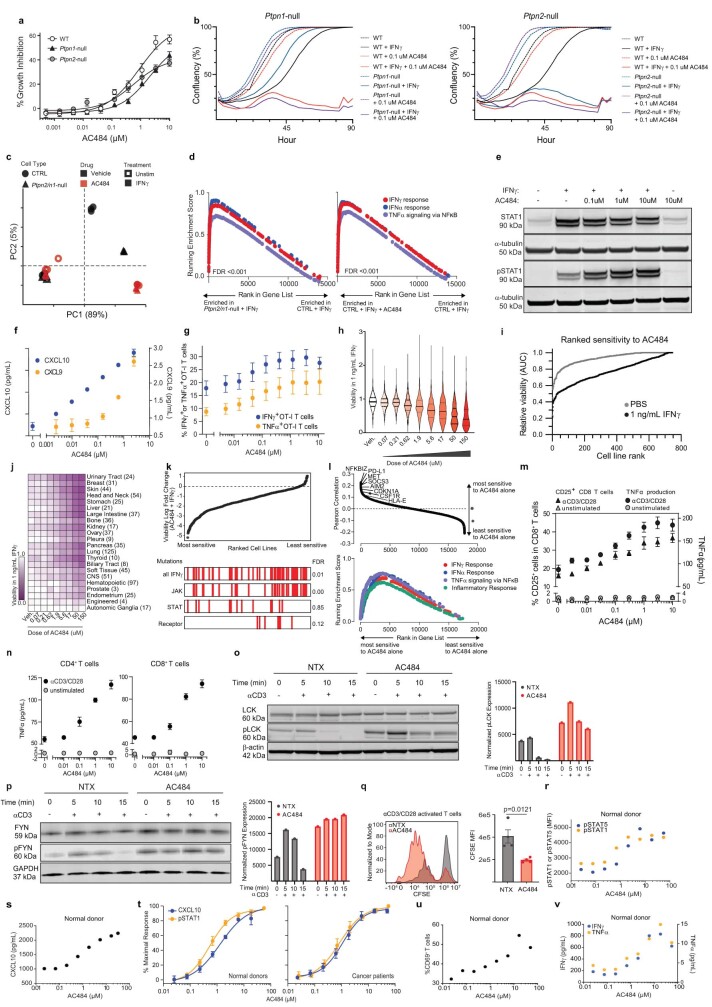
AC484 increases tumour cell sensitivity to IFNγ and immune cell activation in vitro. **a**, IFNγ-induced growth inhibition of wildtype (WT) or *Ptpn1*-null or *Ptpn2*-null B16 tumour cell (n = 3 for each cell line). **b**, In vitro growth curve of control and *Ptpn1*-null (left) and *Ptpn2*-null (right) B16 cells ± AC484 (0.1 µM) and ± IFNγ (10 ng mL^−1^) (n = 2). **c**, PCA of top 500 highly variable genes from in vitro transcriptional profiling of B16 control or *Ptpn2/n1*-null cells ± IFNγ and ± AC484. **d**, GSEA of Hallmark TNFα signaling, IFNγ and IFNα response gene signatures in control or *Ptpn2/n1*-null B16 cells treated with AC484 (10 μM) incubated overnight in the presence of IFNγ (10 ng mL^−1^). **e**, Western blot for total and phosphorylated STAT1 protein in B16 cells that were stimulated for 16 h with IFNγ (100 ng mL^−1^) ± AC484 and then washed out of IFNγ and incubated for 4 h in AC484 only (where applicable). **f**, CXCL10 (blue) and CXCL9 (orange) production by IFNγ-treated B16 tumour cells (n ≥ 4). **g**, B16-OVA tumour cells that were pretreated with IFNγ and AC484 overnight and incubated with OT-I T cells. Percent IFNγ (blue) or TNFα (orange) producing OT-I T cells (n = 4). **h**, Viability at increasing doses of AC484 + IFNγ for the human cell line collection screened with the PRISM pipeline. **i**, PRISM screen cell lines ranked by their sensitivity to AC484 with and without IFNγ (1 ng mL^−1^). **j**, Heatmap showing viability across cancers from different tissue types in the PRISM platform against increasing doses of AC484 + IFNγ. **k**, PRISM screen cell lines ranked by their sensitivity to AC484 + IFNγ (1 ng mL^−1^) (top). Tracks show cell lines with mutations in IFNGR1, IFNGR2, JAK, or STAT genes and the enrichment of these mutations in cell lines that are more or less sensitive to AC484 + IFNγ (bottom). FDR for enrichment of mutant cell lines shown to the right. **l**, Pearson correlation of gene expression in PRISM cell lines with sensitivity to AC484 alone (top) and GSEA of these correlations (bottom). **m**, Activation (Percent CD25-expressing CD8 T cells, n = 6) and TNFα production (n = 8) of anti-CD3/CD28 stimulated primary splenic pan T cells isolated from C57BL/6N mice. **n**, TNFα production of anti-CD3/CD28 stimulated primary splenic CD4 T cells (left, n = 4) and CD8 T cells (right, n = 4) isolated from C57BL6J mice. **o**, Western blot for total LCK and pLCK in naive CD8 T cells stimulated with crosslinked anti-CD3 (2.5 μg mL^−1^) ± AC484 (20 μM) for 0, 5, 10, or 15 min. Quantification of pLCK expression normalized to total LCK at the right. **p**, Western blot for total FYN and pFYN in naive CD8 T cells stimulated with crosslinked anti-CD3 (2.5 μg mL^−1^) ± AC484 (20 μM) for 0, 5, 10, or 15 min. Quantification of pFYN expression normalized to total FYN at the right. **q**, Representative histogram (left) and quantification (right) for CFSE-labeled naïve CD44^+^CD8^+^ T cells from WT mouse spleen sub-optimally stimulated with plate-bound anti-CD3 (1 μg mL^−1^), soluble anti-CD28 (2 μg mL^−1^), IL-2 (100 ng mL^−1^), with or without AC484 (20 μM) for 96 h. T-cell proliferation (CFSE dilution) determined by flow cytometry. **r**-**v**, Representative raw data of AC484 human whole blood pathway engagement and immune activation. **r**, IFNγ-mediated STAT1 phosphorylation and IL-2-induced STAT5 phosphorylation in AC484-treated whole blood from one healthy donor assessed by flow cytometry. **s**, IFNγ-induced CXCL10 (IP10) production in whole blood from one healthy donor. **t**, IFNγ-induced STAT1 phosphorylation (pSTAT1) and CXCL10 (IP10) production in whole blood from healthy donors (left, n = 8) or cancer patients (right, CXCL10 n = 6, pSTAT1 n = 5). **u**, IFNγ and TNFα production following TCR stimulation with Cytostim^TM^ of whole blood from one healthy donor. **v**, Frequency of CD69-expressing T cells following TCR with Cytostim^TM^ of whole blood from one healthy donor. Source data

Genetic ablation of *PTPN2* or *PTPN1* in tumour cells augments signal transduction from type I and type II IFNs, increasing downstream effects, including cell growth arrest, increased production of immune cell chemoattractants and increased antigen presentation^3^. We compared the effect of PTPN2/N1 inhibition to genetic deletion and found that AC484 dose-dependently enhanced IFNγ-driven growth arrest in vitro, comparable to *Ptpn2/n1*-deficient B16 tumour cells (Fig. 2a,b). Furthermore, AC484 demonstrated additive growth inhibitory effects in *Ptpn2*-deficient or *Ptpn1*-deficient B16 tumour cells. This result indicated that the phosphatases have redundant roles and that inhibition of both suppressed tumour cell growth more strongly than inhibiting either alone (Extended Data Fig. 1a,b). Transcriptomic profiling revealed that AC484-treated and *Ptpn2/n1*-deficient tumour cells had highly similar global transcriptional responses to IFNγ treatment (Fig. 2c). In unsupervised principal component analysis (PCA), treatment of unmodified cells with both IFNγ and AC484 clustered near IFNγ-treated *Ptpn2/n1*-deficient cells (Extended Data Fig. 1c). Deletion of both *Ptpn2* and *Ptpn1* led to a stronger induction of IFN-stimulated genes (ISGs) following IFNγ treatment, as measured by the enrichment of the hallmark IFNγ response, IFNα response and TNF signalling through NF-κB gene signatures^18^ (Extended Data Fig. 1d, left). Treatment with both AC484 and IFNγ phenocopied this transcriptional response, showing enrichment of the same signatures compared with cells treated with IFNγ and vehicle control (Extended Data Fig. 1d, right). Notably, cells treated with AC484 alone did not show increased ISG expression, which suggested that AC484 does not spontaneously activate the IFN response (Fig. 2c and Supplementary Table 3). Consistent with the observed increase in ISGs, AC484 and IFNγ treatment enhanced the phosphorylation of STAT1 and the production of IFN-inducible chemokines, including CXCL10 (also known as IP10) and CXCL9 (also known as MIG) (Extended Data Fig. 1e,f). In B16 cells engineered to express the exogenous protein ovalbumin (B16-OVA), AC484 dose-dependently increased the mean fluorescence intensity (MFI) of the OVA-derived immunodominant epitope SIINFEKL presented by MHC class I (H2-K^b^) following stimulation with IFNγ (Fig. 2d,e). Additionally, pretreatment of B16-OVA cells with AC484 led to significantly more tumour killing and cytokine production (IFNγ and TNF) by untreated SIINFEKL-specific OT-I T cells in vitro (Fig. 2f and Extended Data Fig. 1g) than vehicle-treated cells, a result consistent with enhanced antigen presentation. Thus, treatment of tumour cells with AC484 phenocopies the effects of deletion of both *Ptpn2* and *Ptpn1* on cell growth and ISG expression in response to IFN, and enhances tumour sensitivity to T cell-mediated toxicity.

We next used the PRISM platform^19^ to assess the effect of increasing doses of AC484 with and without IFNγ on a broad range of human cancer cell lines to determine cancer cell features that affect sensitivity to AC484. We observed dose-dependent growth inhibition from AC484 treatment only in the presence of IFNγ across the entire human cell line collection (Extended Data Fig. 1h,i). We analysed sensitivity to IFNγ and AC484 in each represented tissue type and found that cancers of the urinary tract, breast, skin, and head and neck were among the most sensitive, whereas tumours of the autonomic ganglia and the endometrium were the most resistant in vitro (Extended Data Fig. 1j). Loss-of-function mutations in the IFNγ-sensing pathway were significantly enriched among the most insensitive tumours (Extended Data Fig. 1k), which confirmed that IFNγ sensing is required for AC484-mediated tumour growth inhibition. Although rare, some cancer cell lines exhibited intrinsic sensitivity to AC484 in the absence of IFNγ, which correlated with increased expression of *NFKBIZ*, *CD274* (which encodes PD-L1), *SOCS3*, *HLA-E*, and several other ISGs (Extended Data Fig. 1l, top). Moreover, gene signatures for IFN response and TNF signalling through NF-κB were highly enriched in intrinsically sensitive cell lines (Extended Data Fig. 1l, bottom), which suggested that cell-intrinsic low-grade inflammation, sometimes called parainflammation^20^, is predictive of intrinsic sensitivity to AC484. Thus, our data suggest that AC484 increases the sensitivity of nearly all human cancer cell lines to IFNγ and inhibits the growth of cancers that exhibit features of intrinsic inflammation.

## AC484 enhances T cell function in vitro

Genetic deletion of *PTPN2* or *PTPN1* in T cells can increase T cell proliferation and cytokine production in vitro and prevent tumour outgrowth in vivo^4–6,11,12^. Activation of primary mouse T cells in vitro in increasing doses of AC484 increased the frequency of activated CD69^+^ and CD25^+^ T cells and the production of the proinflammatory cytokines IFNγ and TNF (Fig. 2g and Extended Data Fig. 1m). The increase in TNF production was observed in both CD4^+^ and CD8^+^ T cells (Extended Data Fig. 1n). Loss of PTPN2 or PTPN1 in T cells can decrease the TCR activation threshold owing to increased LCK and FYN phosphorylation, which could increase the recognition of less immunogenic tumour antigens^11,12^. Indeed, AC484 treatment increased the levels and the duration of LCK and FYN phosphorylation in both resting and anti-CD3-stimulated mouse T cells (Extended Data Fig. 1o,p). In line with these data, AC484 treatment increased the proliferation of T cells activated with suboptimal anti-CD3/CD28 stimulation compared with untreated cells (Extended Data Fig. 1q). Consistent with our results showing an additive effect of dual PTPN2/N1 inhibition in tumour cells, AC484 increased the expression of CD69 in PTPN2-deficient or PTPN1-deficient T cells (Fig. 2h). This result suggests that PTPN2 and PTPN1 have redundant roles in T cells and that dual inhibition further enhances T cell activation. Notably, AC484 only amplified signalling in T cells that were already activated (Fig. 2g,h and Extended Data Fig. 1m,n). Thus, in addition to its effect on tumour cells, PTPN2/N1 inhibition enhances T cell activation, consistent with previously reported effects of genetic ablation of *PTPN2* or *PTPN1*. However, dual inhibition of both phosphatases with AC484 more potently promoted T cell activation than single targeting approaches.

## AC484 drives human immune cell activation

We next assessed the impact of AC484 on human immune cells. We treated pre-stimulated human whole blood with AC484 and measured STAT phosphorylation and immune activation. AC484 dose-dependently increased the phosphorylation of STAT5 to a similar level in whole blood from healthy donors and from patients with cancer (Fig. 2i and Extended Data Fig. 1r). In blood samples from healthy donors and from patients with cancer, AC484 increased IFNγ-induced STAT1 phosphorylation and CXCL10 production, which suggested that AC484-enhanced pathway engagement resulted in amplified downstream functional effects (Extended Data Fig. 1s,t). We also confirmed that AC484 increased T cell activation and function in human whole blood, as indicated by the dose-dependent increase in CD69 expression and in IFNγ and TNF production after TCR stimulation (Fig. 2j and Extended Data Fig. 1u,v). Gene expression analyses of peripheral blood mononuclear cells (PBMCs) isolated from TCR-stimulated whole blood confirmed the immune-stimulatory effects of AC484, as evidenced by the increased expression of *TBX21* (which encodes TBET), *ICOS* and *CXCL11* (Fig. 2k and Supplementary Table 4). These findings show that inhibition of PTPN2/N1 by AC484 enhances human immune cell activation.

## AC484 potently controls tumour growth in mice

**Extended Data Fig. 2:**
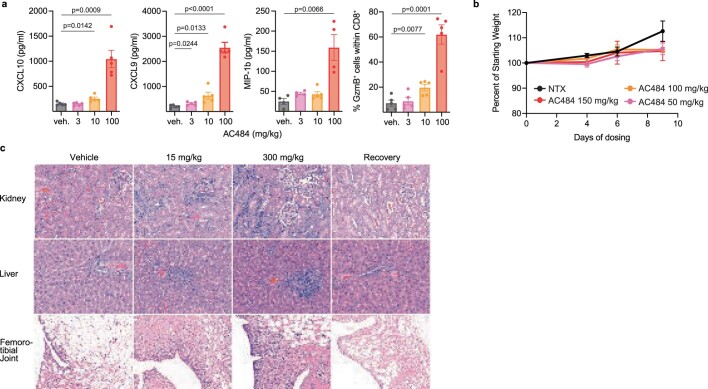
AC484 demonstrates dose-dependent immune activation and reversible immune infiltration of tissues in vivo. **a**, Circulating chemokines (CXCL10, CXCL9, MIP-1b) and frequency of circulating Granzyme B expressing CD8^+^ T cells in MC38-tumour bearing mice after 8 days of dosing with 3, 10, and 100 mg/kg/day AC484. **b**, Weight change over time of B16 tumour-bearing C57BL/6J mice treated with AC484 (50, 100 or 150 mg/kg) compared with untreated controls. **c**, Inflammatory cell infiltrates in tissues of male rats dosed with AC484 for 28 days with resolution after a 28-day recovery period. Sections of kidney, liver, and femoro-tibial joint from animals administered vehicle, 15 mg/kg/day, or 300 mg/kg/day AC484 and from recovery animals previously administered 300 mg/kg/day AC484 as indicated. Source data

*PTPN2* loss-of-function single-nucleotide polymorphisms are associated with autoimmune disorders in humans, and mice with global *Ptpn2* deletion succumb to systemic immune activation a few weeks after birth^21–23^. Thus, we sought to determine whether systemic inhibition of PTPN2/N1 through oral administration could be used as a safe and effective immunotherapy. We tested the systemic immune effects of AC484 across a wide range of doses, ranging from 3 mg kg^–1^ up to 100 mg kg^–1^ twice daily dosing. Circulating chemokines, including CXCL10 and CXCL9, increased by 2–3-fold at 10 mg kg^–1^ twice daily dosing (Extended Data Fig. 2a), a result consistent with reported increases induced by anti-PD-1 therapy in human trials^24^. By contrast, doses of 100 mg kg^–1^ twice daily led to overt systemic immune activation in some animals, manifested by a significant increase in circulating cytotoxic granzyme B (GZMB)^+^ CD8^+^ T cells and cytokines such as CXCL10 and CXCL9 (Extended Data Fig. 2a). We also evaluated once-daily dosing at 50, 100 and 150 mg kg^–1^ in tumour-bearing animals and observed no significant change in body condition score or weight (Extended Data Fig. 2b). Additionally, AC484 was tolerated in naive rats up to 300 mg kg^–^^1^ per day for 28 days. The most notable findings were dose-dependent immune cell infiltrates in kidneys, joints and livers (Extended Data Fig. 2c and Extended Data Table 6). The infiltrates, however, resolved within 28 days of treatment cessation (Extended Data Fig. 2c and Extended Data Table 6). This reversible immune activation effect highlights the benefit of a small-molecule approach. That is, the relatively short half-life of AC484 enables rapid cessation of treatment to resolve inflammatory conditions more rapidly than antibody-based therapeutics.

**Fig. 3:**
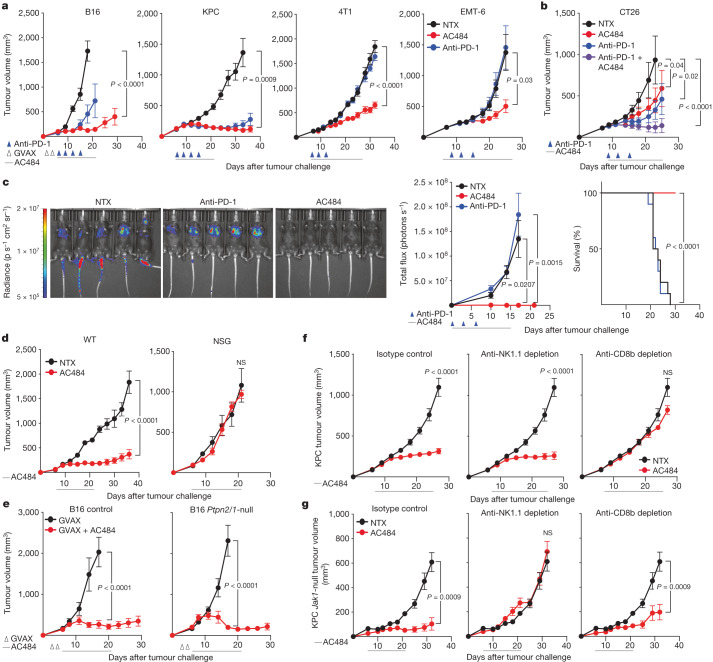
Systemic administration of AC484 induces immune-dependent tumour regression in various syngeneic and metastatic mouse models. **a**, Tumour growth over time of B16 melanoma, KPC pancreatic cancer, and 4T1 and EMT-6 breast cancer tumours in AC484-treated (red), anti-PD-1-treated (blue) or control animals (black) (*n* = 5–10 animals per group, data are the mean ± s.e.m.). **b**, Tumour growth over time of CT26 colon cancer tumours in AC484-treated (red), anti-PD-1-treated (blue) AC484-treated and anti-PD-1-treated (purple) or control animals (black) (*n* = 10 animals per group, data are the mean ± s.e.m., statistics calculated as per cent tumour growth inhibition) **c**, Representative luciferase imaging at day 17 after challenge (left), tumour growth over time (middle) and survival analysis (right) of B16 metastasis model mice in AC484-treated (red), anti-PD-1-treated (blue) or control animals (black) (*n* = 10 per group). **d**, Tumour growth over time for KPC tumours in WT mice with or without AC484 (*n* = 10, *n* = 5, respectively), and for NSG mice with or without AC484 (*n* = 10, *n* = 5, respectively). **e**, Tumour growth over time for control of *Ptpn2/n1*-null B16 tumours treated with GVAX with or without AC484 (*n* = 5 animals per group, data are the mean ± s.e.m.). **f**, Tumour growth over time for KPC tumours treated with an isotype antibody (*n* = 10), anti-CD8b (*n* = 10) or an anti-NK1.1 depleting antibody (*n* = 10) and treated with AC484 (red) compared with an untreated control group (*n* = 10; black). **g**, Tumour growth over time for *Jak1*-null KPC tumours treated with isotype antibody (*n* = 5), anti-CD8b (*n* = 10) or anti-NK1.1 depleting antibody (*n* = 10) and treated with AC484 (red) compared with an untreated control group (*n* = 10; black). Source data

**Extended Data Fig. 3:**
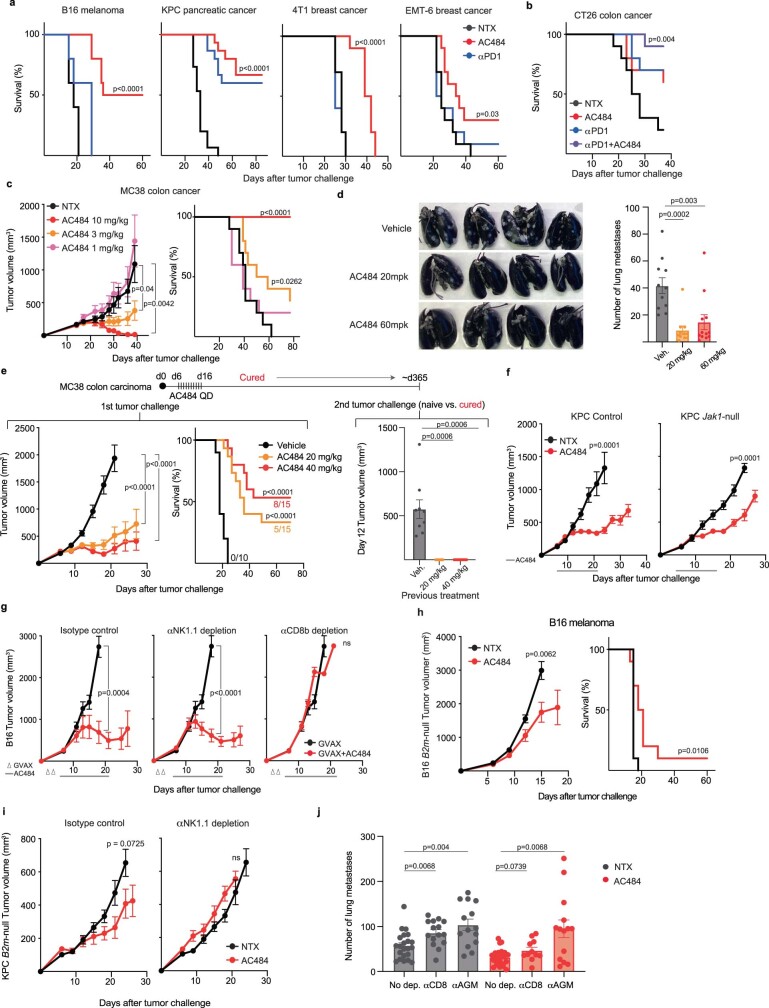
AC484 induces CD8^+^ T cell- and NK cell-mediated tumour regression in a variety of syngeneic and metastatic mouse models. **a**, Survival analysis of animals bearing B16, KPC, 4T1, and EMT-6 tumours and treated with AC484 (red), anti-PD-1 (blue), or no treatment (black). Significance values reported for NTX v. AC484 in each. **b**, Survival analysis of animals bearing CT26 tumours and treated with AC484 (red), anti-PD-1 (blue), AC484 and anti-PD-1 (purple) or no treatment (black). Significance value reported for NTX vs. anti-PD-1 and AC484. **c**, Tumour growth over time and survival analysis of MC38 tumours in untreated or AC484-treated (1, 3, or 10 mg/kg) animals. Significance values reported for NTX vs. AC484 (3 mg/kg) and NTX vs. AC484 (10 mg/kg). **d**, Representative images of 4T1 lung metastases visualized in 15% India ink (left) and lung metastasis counts (right) for AC484-treated (20 mg/kg or 60 mg/kg) and vehicle-treated mice (n = 11-12 animals per group) on day 29 post-challenge **e**, Tumour growth over time and survival analysis for MC38 tumour-bearing mice treated with 20 mg/kg or 40 mg/kg doses of AC484 (left). Naive mice and mice that cleared tumours (cured) were rechallenged with MC38 tumour cells 1 year later. Bar plot showing tumour volumes on day 12 after rechallenge (right). **f**, Tumour growth over time for control or *Jak1*-null KPC tumours ± AC484 (n = 5–10 animals per group, data are mean ± SEM) **g**, Tumour growth over time for GVAX-treated B16 tumours treated with either isotype antibody (n = 10), anti-CD8 (αCD8, n = 10) or anti-NK depleting antibody (αNK1.1, n = 10), and treated with AC484 (red) compared to an untreated control group (n = 10; black). **h**, Tumour growth over time and survival analysis for *B2m*-null B16 tumours treated with AC484 (red) or untreated (black). **i**, Tumour growth over time for *B2m*-null KPC tumours treated with anti-NK depleting antibody (n = 10) or no depletion (n = 10) and treated with AC484 (red) compared to an untreated control group (n = 10; black). **j**, 4T1 lung metastasis counts for mice treated with either anti-CD8, anti-NK depleting antibody, or no depleting antibody, and treated with AC484 (red) compared to untreated control groups (black) (n = 11–23 animals per group). Source data

Having shown that AC484 is well-tolerated up to 150 mg kg^–1^ once-daily dosing, we next compared the efficacy of AC484 treatment to anti-PD-1 blockade in mice bearing B16, KPC (*Kras*^*G12D/+*^*;Trp53*^*R172H/+*^) pancreatic adenocarcinoma, 4T1 mammary carcinoma or EMT-6 breast carcinoma subcutaneous tumours. Treatment with AC484 induced highly significant tumour regression and increased survival in all four models (Fig. 3a and Extended Data Fig. 3a). In each model, tumour regression and survival induced by AC484 were comparable to or greater than anti-PD-1 treatment (Fig. 3a and Extended Data Fig. 3a), especially in the PD-1-resistant 4T1 and EMT-6 models. The combination of AC484 with anti-PD-1 in the CT26 model further enhanced efficacy and survival, which indicated the additive effect of the two therapies (Fig. 3b and Extended Data Fig. 3b). Similar efficacy was observed with AC484 doses as low as 3 mg kg^–1^ in MC38 colorectal carcinoma (Extended Data Fig. 3c).

Metastasis is the leading cause of cancer-related death^25^, and we next evaluated AC484 efficacy in syngeneic metastasis models. We first used the B16 pulmonary metastasis model of intravenous injection of luciferase-expressing B16 cells, which seed the lungs with tumours. Mice in the untreated and anti-PD-1 cohorts developed lung metastases by day 10, whereas AC484-treated mice developed no detectable disease and had a 100% survival rate (Fig. 3c). To extend these results to a model in which metastases arise from a primary tumour, we used the 4T1 orthotopic breast cancer model, in which tumour cells metastasize to the lungs from a primary tumour in the mammary fat pad^26^. Treatment with AC484 reduced the number of lung metastases (Extended Data Fig. 3d). Together, these data show that treatment with AC484 not only enhances the control of established tumours but also improves the surveillance of disseminated metastatic disease.

## PTPN2/N1 inhibition in immune cells is sufficient for efficacy

We next sought to further understand the mechanism by which AC484 mediates tumour growth inhibition. First, to confirm that AC484 efficacy is immune-dependent, we challenged wild-type (WT) C57BL/6J mice or NOD-scid IL-2Rg^null^ (NSG) mice with KPC tumours and treated both cohorts with vehicle control or AC484. AC484 treatment controlled tumour growth in WT but not NSG mice (Fig. 3d). To test whether AC484 responses generated immune memory, we performed a rechallenge of MC38 subcutaneous tumours in naive mice and in mice that had previously cured MC38 tumours following AC484 treatment. Rechallenged mice but not naive mice demonstrated resistance of tumour growth (Extended Data Fig. 3e), which suggested that AC484 treatment supports immune memory.

Loss of PTPN2 and PTPN1 in tumours increases the sensitivity to IFN and can enhance immune sensitivity and immune infiltration through the upregulation of tumour cell MHC class I and IFNγ-regulated chemokines^3^. However, consistent with published reports^4,5,11^, we showed that PTPN2/N1 inhibition enhances T cell activation (Fig. 2i,j), which could also increase anti-tumour immunity. We next evaluated the tumour and immune-mediated effects of systemic PTPN2/N1 inhibition and their relative contributions to anti-tumour efficacy in vivo. To determine whether AC484 efficacy requires PTPN2/N1 inhibition in tumour cells, we implanted *Ptpn2/n1*-deficient or control B16 tumours into mice, treated all the mice with a low dose of granulocyte-macrophage colony-stimulating factor (GM-CSF)-secreting, irradiated tumour cell vaccine (GVAX), and compared the effect of AC484 or vehicle treatment. *Ptpn2/n1*-null tumours grew at the same rate as control tumours in mice receiving only GVAX (Fig. 3e), a result consistent with our previous findings^3^. AC484 also enhanced immune control of *Ptpn2/n1*-null and control tumours to a similar extent (Fig. 3e), which indicated that PTPN2/N1 inhibition in immune cells is sufficient to drive efficacy. To corroborate this finding in another model system, we generated *Jak1*-deficient KPC tumours, which lack the ability to sense IFNγ but respond to immunotherapy in vivo^27^. AC484 treatment reduced tumour growth in both *Jak1*-deficient tumours and control KPC tumours (Extended Data Fig. 3f). Thus, tumour IFN sensing is not absolutely required for the efficacy of AC484, which suggests that its effects on host immune cells are sufficient to induce anti-tumour immunity.

## CD8^+^ and NK cells mediate AC484 efficacy

We next set out to determine which immune populations are required for AC484 efficacy. First, we evaluated subcutaneous tumour models using antibody-mediated depletion of CD8^+^ T cells or natural killer (NK) cells. In both KPC tumours and B16 tumours, CD8^+^ T cells but not NK cells were required for tumour control in AC484-treated mice (Fig. 3f and Extended Data Fig. 3g). However, we have previously shown that immunity in *Jak1*-deficient tumours, which express low levels of MHC class I molecules, depends on NK cells^27^. Consistent with this observation, AC484 efficacy in *Jak1*-deficient KPC tumours was abrogated by the depletion of NK cells but not CD8^+^ T cells (Fig. 3g). To extend this finding to additional contexts associated with immune checkpoint blockade resistance, we next evaluated *B2m*-deficient B16 tumours and KPC tumours and observed a response to AC484 treatment in both models (Extended Data Fig. 3h,i). In *B2m*-null KPC tumours, the effect of AC484 depended on the presence of NK cells (Extended Data Fig. 3i). As NK cells can mediate surveillance of metastasis^28,29^, we next assessed the 4T1 lung metastasis model and showed that AC484 efficacy was unaffected by the depletion of CD8^+^ T cells but was reversed after NK cell depletion (Extended Data Fig. 3j). Together, our depletion studies suggest that AC484 treatment simultaneously enhances the activity of multiple immune effector populations, including CD8^+^ T cells and NK cells. These subsets mediate tumour growth in a context-specific manner, whereby CD8^+^ T cells but not NK cells control MHC class I-proficient tumours, whereas NK cells control tumours with impaired IFN sensing and MHC class I expression. Furthermore, the enhancement of NK surveillance also underlies AC484-mediated suppression of metastasis. The ability of AC484 to enhance the activity of multiple cytotoxic immune subsets highlights its distinct mechanism of action relative to anti-PD-1 therapy. Moreover, our results suggest that AC484 may have efficacy in a broader range of contexts alone or in combination with anti-PD-1.

## AC484 inflames the tumour microenvironment

**Fig. 4:**
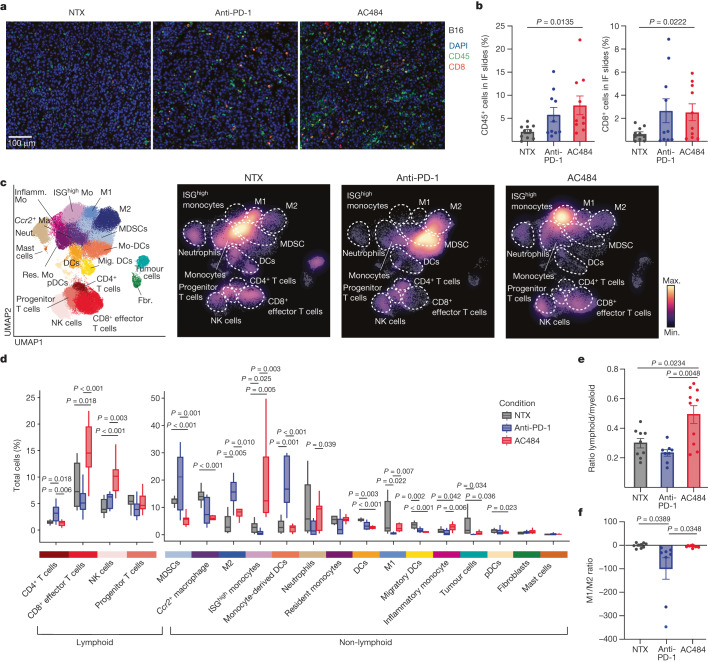
AC484 inflames the TME. **a**, Immunofluorescence (IF) microscopy of representative formalin-fixed paraffin-embedded tumour sections from B16 untreated, anti-PD-1-treated or AC484-treated tumours. Staining: DAPI, blue; CD45, green; CD8, red. **b**, Quantification of CD45^+^ (left) and CD8^+^ (right) cells from B16 tumours from **a**. **c**, Uniform manifold approximation and projection (UMAP) of 68,060 cells and 21 distinct clusters identified among CD45^+^-enriched immune cells (left). Cell density projections by condition (right). DCs, dendritic cells; Fbr., fibroblasts; Inflamm., inflammatory; Ma, macrophage; Mig. DCs, migratory DCs; Mo, monocyte; Mo-DCs, monocyte-derived DCs; Neut., neutrophil; pDCs, plasmacytoid DCs. **d**, Box plots of proportional changes by cluster of CD45^+^-enriched immune cells by treatment. **e**, Ratio of cells belonging to lymphoid-derived clusters versus myeloid-derived clusters by condition. **f**, Directional ratio of cells belonging to clusters identified as M1 macrophages versus M2 macrophages by condition. Source data

**Extended Data Fig. 4:**
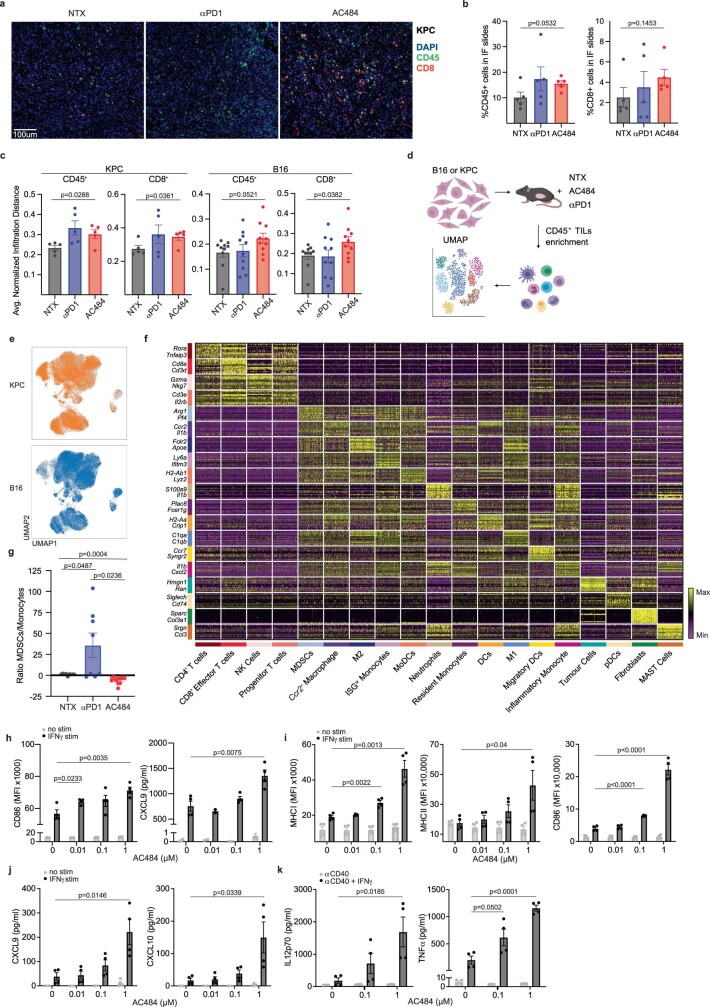
AC484 enhances the activation of immune cell subsets and promotes immune infiltration of tumours. **a**, Immunofluorescence microscopy of representative FFPE tumour sections from KPC untreated, anti-PD-1- or AC484-treated tumours, CD45 (green); CD8 (red); DAPI (blue). **b**, Quantification of CD45^+^ (left) and CD8^+^ (right) cells from KPC tumours from (a). **c**, Barplot showing the average normalized infiltration distance of CD45^+^ and CD8^+^ cells from the tumour border in immunofluorescence stains of KPC and B16 tumours. Infiltration distances are normalized as a fraction of the maximal distance found in each tumour. **d**, Schematic representing experimental design for single cell RNA sequencing on tumour-infiltrating lymphocytes from B16 or KPC tumours from control (n = 9), anti-PD-1- (n = 8) or AC484-treated (n = 10) tumours; figure created with BioRender.com. **e**, UMAP projection showing adequate batch correction of all cells with KPC recovered cells colored orange (top) and B16 colored blue (bottom). **f**, Heatmap showing top 15 differentially expressed genes in each cluster by one vs. rest test with two representative genes from each cluster shown on the y axis. **g**, Directional ratio of MDSCs versus monocytes by condition. **h**, In vitro CD86 expression (n = 6, left) and CXCL9 production (n = 4, right) by BMDM ± IFNγ. **i**, In vitro MHC I (left), MHC II (middle) and CD86 (right) expression on CD103^+^ BMDC ± IFNγ (n = 4). **j**, In vitro CXCL9 (left) and CXCL10 (right) production in CD103^+^ BMDC ± IFNγ (n = 4). **k**, In vitro IL12p70 and TNFα production by CD103^+^ BMDC ± IFNγ + anti-CD40 (αCD40, n = 4). Source data

We next sought to profile the changes in immune infiltration and cell state induced by AC484 in comparison to anti-PD-1. Immunofluorescence microscopy analyses revealed that treatment with AC484 increased the frequency of tumour-infiltrating CD45^+^ and CD8^+^ cells in B16 and KPC tumours, comparable to changes elicited by anti-PD-1 (Fig. 4a,b and Extended Data Fig. 4a,b). Additionally, measuring the average distance from the tumour margin for every CD45^+^ or CD8^+^ cell showed that immune cells in AC484-treated mice infiltrated deeper into the tumour compared with untreated mice (Extended Data Fig. 4c). Thus, PTPN2/N1 inhibition leads to a greater infiltration of total immune cells and CD8^+^ cells into tumours.

We next profiled the effects of AC484 treatment on the phenotype of tumour-infiltrating immune cells using single-cell transcriptional profiling. We performed single-cell RNA sequencing (scRNA-seq) of CD45^+^ tumour-infiltrating lymphocytes (TILs) from B16 tumours and KPC tumours from untreated, AC484-treated or anti-PD-1-treated mice (Fig. 4c, Extended Data Fig. 4d–f and Supplementary Table 5). AC484 induced large shifts in the proportion and transcriptional state of many immune subsets, including an increase in the overall lymphoid to myeloid cell ratio driven by a significant expansion in the relative frequencies of CD8^+^ T cells and NK cells (Fig. 4c–e). We also observed that AC484 elicited a significant increase in the ratio of M1 macrophages to M2 macrophages and the ratio of monocytes to myeloid-derived suppressor cells (MDSCs) (Fig. 4f and Extended Data Fig. 4g). Notably, AC484 also induced a distinct population of monocytes, characterized by the high expression of ISGs, and a marked reduction in MDSCs (Fig. 4c,d). In support of this observation, AC484 enhanced the activation and cytokine production of IFN-stimulated mouse bone-marrow-derived macrophage monocultures in vitro, as measured on the basis of the upregulation of the activation marker CD86 and secretion of the T cell chemoattractant CXCL9 (Extended Data Fig. 4h). Similarly, in bone-marrow-derived dendritic cells (BMDCs), AC484 induced the expression of MHC class I and class II molecules (Extended Data Fig. 4i) and promoted the activation of CD103^+^ BMDCs through the upregulation of CD86 and the production of CXCL9 and CXCL10 (Extended Data Fig. 4i,j). Finally, in CD103^+^ BMDCs activated with agonistic anti-CD40 antibody, AC484 significantly increased IL-12 and TNF production (Extended Data Fig. 4k). These data show that PTPN2/N1 inhibition elicits a proinflammatory phenotype in myeloid subsets that is characteristic of an inflamed tumour microenvironment (TME).

**Extended Data Fig. 5:**
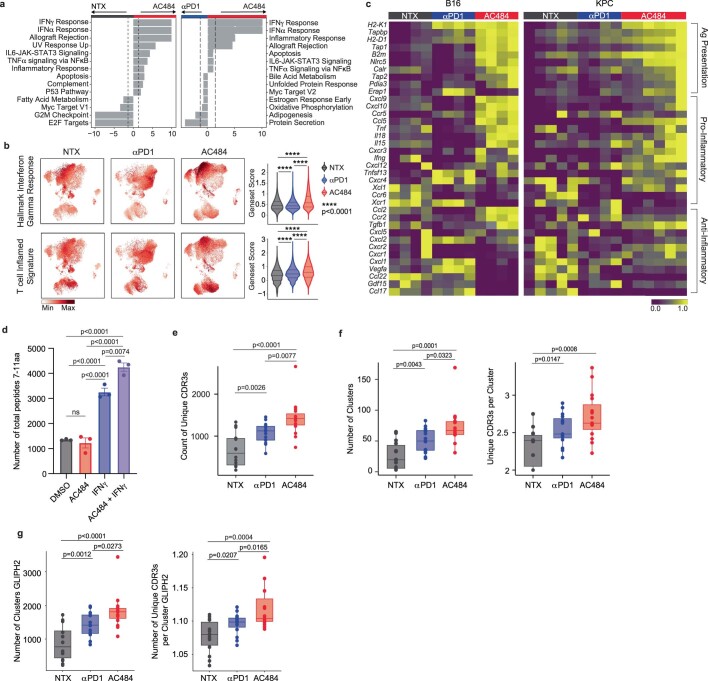
AC484 inflames the TME and enhances the repertoire of the tumor-directed T cell response. **a**, Top positively or negatively enriched Hallmark gene signatures determined by GSEAPreranked on differentially expressed genes calculated by a logistic regression by condition. Dotted lines indicate FDR < 0.25. **b**, UMAP projections downsampled by condition of the Hallmark IFNγ Response gene set and T cell Inflamed Signature (left) (Methods). Violin plots showing quantification by condition, statistical significance <0.05 in an independent t test (right). **c**, Heatmap showing averaged pseudobulk expression of key pro-inflammatory and anti-inflammatory cytokines and chemokines and antigen processing-related genes by model. **d**, Number of 7–11 amino acid length peptides presented on MHC I of B16 tumour cells treated in vitro overnight with DMSO as control, 0.3 μM AC484 alone, 0.5 ng/mL IFNγ alone, and 0.5 ng/mL IFNγ + 0.3 μM AC484. **e**, Counts of unique CDR3 sequences captured in targeted PCR of α and β TCR transcript from bulk RNA of B16 untreated (n = 14), anti-PD-1 (n = 15), and AC484 (n = 15) treated tumours. **f**, Quantification of the number of clusters or recognition groups found in each replicate (left) and number of unique CDR3s per each cluster by replicate (right). **g**, CDR3 sequences amplified from B16 untreated, AC484, or anti-PD-1-treated tumours clustered by GLIPH2 into proposed antigen recognition groups, number of clusters (left) and unique CDR3 sequences per cluster (right). Source data

Differential expression and gene set enrichment analysis (GSEA) of TILs from treated tumours showed a broad upregulation of inflammation-related gene programmes, including several JAK–STAT signalling pathways^18^ (Extended Data Fig. 5a). Higher expression of ISGs and upregulation of a T cell-inflamed gene expression signature were observed across several immune subsets (Extended Data Fig. 5b). This result shows that inhibition of PTPN2/N1 enhances multiple JAK–STAT signalling pathways, which results in immune activation and IFN-mediated inflammation.

Finally, we aimed to assess the effects of therapy on the inflammatory milieu of the TME. We analysed the effect of AC484 on the expression of MHC class I-related antigen-presentation genes and key proinflammatory and anti-inflammatory cytokines and chemokines in pseudobulk analyses of scRNA-seq data of TILs from B16 tumours and KPC tumours (Extended Data Fig. 5c and Supplementary Table 6). We observed significant increases in the expression of proinflammatory cytokines and chemokines such as *Ifng*, *Tnf*, *Il15*, *Il18*, *Cxcl9*, *Cxcl10*, *Cxcl12* and *Ccl5* in AC484-treated tumours (Extended Data Fig. 5c and Supplementary Table 6). AC484 treatment also induced a marked reduction in the expression of the chemokines *Ccl22* and *Ccl17*, which have been implicated in the recruitment of regulatory T cells in tumours^30^ (Extended Data Fig. 5c). Thus, systemic inhibition of PTPN2/N1 causes proinflammatory remodelling of the TME to support anti-tumour immunity. Notably, the microenvironment-remodelling effects of AC484 are distinct from those induced by anti-PD-1, consistent with the different mechanism of action of AC484 that affects a broader repertoire of immune cells.

## AC484 increases TCR diversity in tumours

Given our observation of increased antigen presentation in AC484-treated tumour cells both in vitro and in vivo (Fig. 2d–f and Extended Data Fig. 5c), we next evaluated whether inhibition of PTPN2/N1 elicited changes in the peptide repertoire presented on MHC class I molecules of tumour cells. A higher number of distinct peptides were recovered from IFNγ-stimulated B16 cells treated with AC484 compared with cells treated with IFNγ or AC484 alone (Extended Data Fig. 5d). As PTPN2/N1 inhibition lowered the threshold for TCR signalling and T cell activation (Fig. 2g,h and Extended Data Fig. 1j–n), in addition to enhancing the presentation of tumour antigens, AC484 may affect the repertoire of tumour-reactive T cells. To assess TCR diversity, we profiled the TCRβ chain repertoire from B16 tumours that were untreated or treated with anti-PD-1 or AC484. AC484 induced a more diverse TCR response, as measured by the increase in specific CDR3 recovery compared with untreated or anti-PD-1-treated groups (Extended Data Fig. 5e). These increases could be the result of a greater diversity of TCRs recognizing the same dominant tumour antigens, new T cell responses generated against weak tumour antigens or a combination of both mechanisms. To better understand how AC484 treatment diversifies the T cell response, we clustered the distinct TCRs recovered from each condition into putative shared recognition groups by edit distance and evaluated the number of clusters and sequence diversity within clusters^31^. AC484 treatment led to an increase in the number of antigen recognition groups relative to those treated with anti-PD-1 or vehicle (Extended Data Fig. 5f, left). Additionally, the number of specific CDR3 sequences per recognition group from AC484-treated tumours was significantly larger than the vehicle-treated group and trending higher than the anti-PD-1-treated group (Extended Data Fig. 5f, right). We confirmed these results using GLIPH2, an algorithm that considers conserved short sequence motifs instead of single amino acid edits to assess TCR similarity^32^ (Extended Data Fig. 5g).

## AC484 induces a specific CD8^+^ effector T cell state

**Fig. 5:**
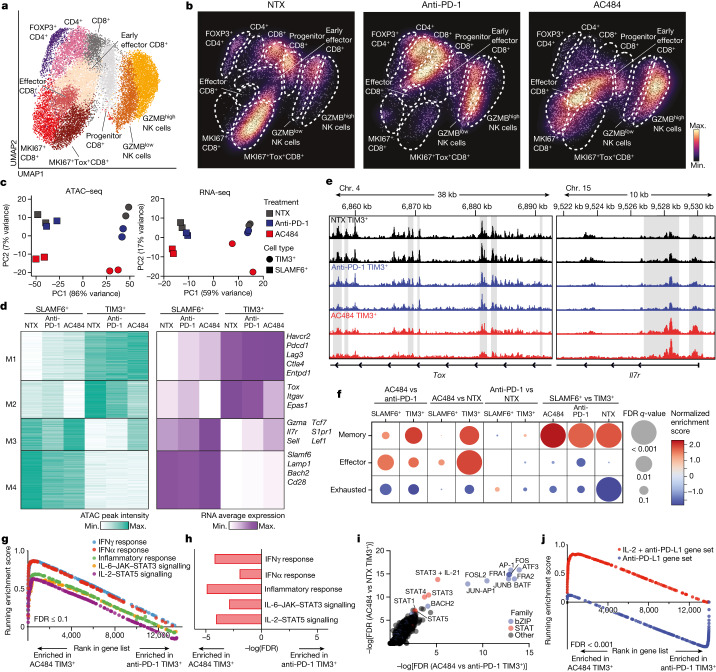
Systemic administration of AC484 in mice enhances IL-2–pSTAT5 signalling in the TME and induces a distinct T cell differentiation state. **a**, UMAP of 20,035 re-clustered lymphoid cells that belonged to clusters identified in the original projection as expressing transcripts for *Cd8a*, *Ncr1* or *Cd4*. **b**, Cell density projections by condition. **c**, PCA of ATAC–seq and RNA-seq samples. **d**, Unbiased *K*-means clustering of normalized peak intensity for differential OCRs (left). Average normalized mRNA expression of genes adjacent to peaks in each module (right). In both plots, values represent the average of two replicates. **e**, ATAC–seq tracks of the *Tox* and *Il7r* loci for TIM-3^+^ samples. Grey shaded regions are significantly differential between conditions. Two replicates shown per condition. **f**, Heatmap of RNA-seq-derived GSEA for memory, effector and exhausted CD8^+^ T cell gene sets (Methods) in all relevant pairwise comparisons. **g**, RNA-seq-derived GSEA of hallmark IFNγ, IFNα and inflammatory response, and IL-6–JAK–STAT3 and IL-2–STAT5 signalling gene sets significantly enriched in AC484 TIM-3^+^ compared with anti-PD-1 TIM-3^+^ samples. False discovery rate (FDR) for all was <0.1. **h**, Differential enrichment measured by hypergeometric test of hallmark gene sets in adjacent genes of differential OCRs between AC484 TIM-3^+^ and anti-PD-1 TIM-3^+^ conditions. **i**, TF motif enrichment analysis (Methods) of differential OCRs in AC484-treated TIM-3^+^ T cells relative to control (*y* axis) or anti-PD-1 (*x* axis) treated TIM-3^+^ samples. –log(FDR) values calculated using binomial tests are plotted on the axes. **j**, GSEA of IL-2 and anti-PD-L1 and of anti-PD-L1 gene sets (data are from ref. ^37^) between AC484 TIM-3^+^ and anti-PD-1 TIM-3^+^ RNA-seq. FDR for both was <0.001. Source data

**Extended Data Fig. 6:**
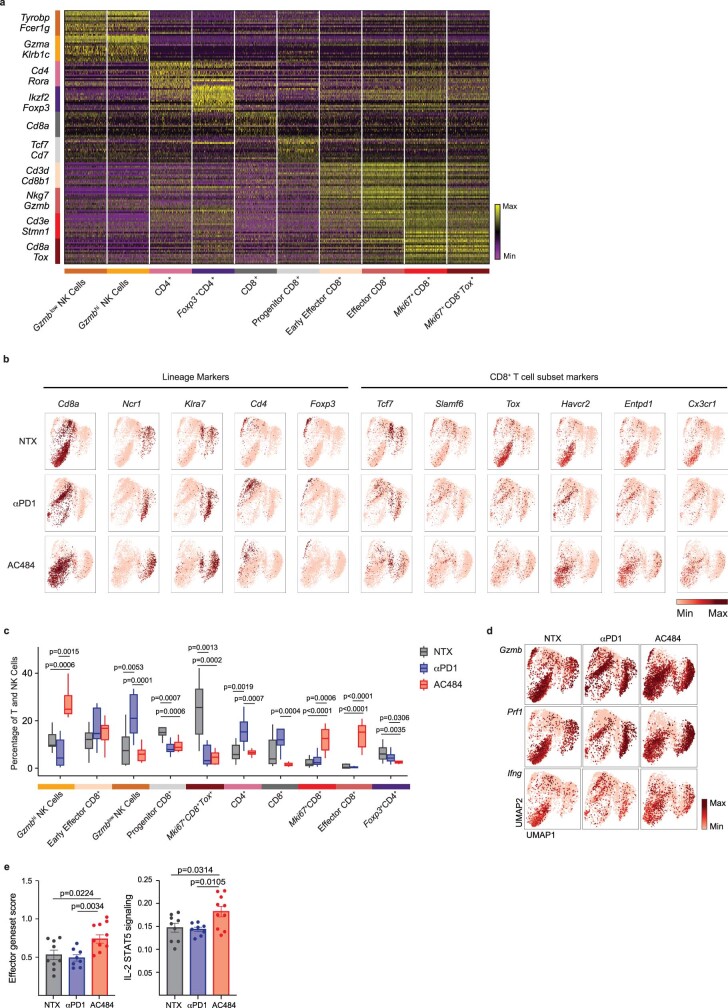
AC484 induces a unique CD8+ effector T cell state. **a**, Heatmap showing top 15 differentially expressed genes in each cluster by one vs. rest test with representative genes from each cluster shown on the y axis. **b**, Relative expression of lineage and CD8^+^ T cell subset markers among re-clustered lymphoid cells expressing *Cd8a*, *Ncr1*, or *Cd4* from Fig. 5a. **c**, Box plots of proportional changes by cluster of lymphoid cells by treatment. **d**, UMAP projections showing capture and expression of *Gzmb* (top), *Prf1* (middle) and *Ifng* (bottom), downsampled for an equal number of cells across conditions. **e**, Bar plot showing scoring of a previously published effector T cell geneset (GSE9650_NAIVE_VS_EFF_CD8_TCELL_DN) in all clusters with captured *Cd8a* transcript averaged by replicate (left). Bar plot showing scoring of the Hallmark IL-2/STAT5 Signaling gene set in all clusters with captured *Cd8a* transcript averaged by replicate (right). Source data

To further investigate PTPN2/N1 inhibition in lymphocytes, we separately re-clustered CD4^+^ T cells, CD8^+^ T cells and NK cells, which enabled higher resolution clustering of phenotypically distinct populations of T cells and NK cells (Fig. 5a, Extended Data Fig. 6a,b and Supplementary Table 7). AC484 treatment induced a significant increase in the number of cytotoxic GZMB^high^ NK cells and a relative depletion of FOXP3^+^ regulatory T cells (Fig. 5b and Extended Data Fig. 6c), consistent with our results showing a decrease in the expression of chemokines that recruit regulatory T cells (Extended Data Fig. 4j).

Although the untreated and anti-PD-1-treated conditions contained progenitor, early effector and exhausted T cell populations that have been previously described^33–35^, AC484 induced a distinct effector CD8^+^ T cell population that expressed high levels of cytotoxic and effector genes such as *Gzmb*, *Prf1* and *Ifng* (Fig. 5b and Extended Data Fig. 6c,d). This population was not observed in untreated or anti-PD-1-treated tumours but was the most abundant CD8^+^ T cell population in AC484-treated tumours (Fig. 5b and Extended Data Fig. 6c,d). A proliferating but similar subset with increased *Mki67* expression was also AC484 specific (Fig. 5b and Extended Data Fig. 6c). GSEA of AC484 compared with anti-PD-1 or untreated tumours revealed enrichment of an effector T cell signature and the *IL2*–*STAT5* gene signature (Extended Data Fig. 6e), a result consistent with increased JAK–STAT signalling caused by PTPN2/N1 inhibition.

## AC484 prevents T cell exhaustion

**Extended Data Fig. 7:**
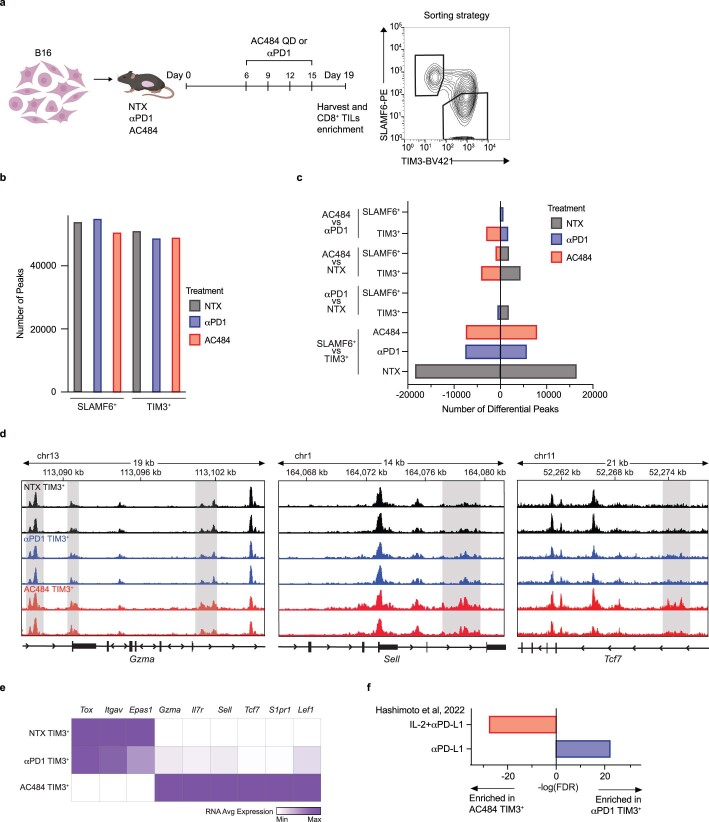
AC484 induces transcriptional and epigenetic programs that promote effector function and reduce T cell exhaustion. **a**, Schematic representing experimental design for ATAC-seq and RNAseq sequencing of tumour-infiltrating lymphocytes from B16 tumours from untreated (n = 14), anti-PD-1- (n = 14) or AC484-treated (n = 19) mice; figure created with BioRender.com. **b**, Number of called peaks for each sample condition (Methods). **c**, Number of differential open chromatin regions between conditions (FDR < 0.05). Top row shows the comparison of AC484 SLAMF6^+^ vs anti-PD-1 SLAMF6^+^. Bottom row shows the comparison of NTX SLAMF6^+^ vs NTX TIM-3^+^. Color denotes the condition in which regions are differentially open. **d**, ATAC-seq tracks of the *Gzma*, *Sell*, and *Tcf7* loci for TIM-3^+^ samples. Gray shaded regions are significantly differential between conditions. Two replicates shown per condition. **e**, Average normalized mRNA expression for genes called out in Fig. 5d. Colors are scaled per each column individually. **f**, Differential enrichment calculated by hypergeometric test of IL-2+anti-PD-L1 and anti-PD-L1 genesets in adjacent genes of differential OCRs between AC484 TIM-3^+^ and anti-PD-1 TIM-3^+^. Red denotes enrichment in AC484 TIM-3^+^. Blue denotes enrichment in anti-PD-1 TIM-3^+^. Source data

Previous work has shown that loss of PTPN2 in T cells enhances the formation of a terminally exhausted CD8^+^ cell population characterized by high expression of TIM-3 and TOX^5,36^. To study the impact of AC484 treatment on CD8^+^ T cell exhaustion, we sorted tumour-infiltrating SLAMF6^+^ (progenitor exhausted) and TIM-3^+^ (terminally exhausted) CD8^+^ T cells from untreated mice, AC484-treated mice and anti-PD-1-treated mice, and performed bulk RNA-seq and ATAC–seq (Extended Data Fig. 7a,b). SLAMF6^+^ and TIM-3^+^ samples clustered distinctly in both transcriptional and epigenetic PCA, whereas AC484 treatment drove additional separation within the SLAMF6^+^ and TIM-3^+^ subsets (Fig. 5c). Consistent with this result, we identified the most differential peaks between the SLAMF6^+^ and TIM-3^+^ subsets within each treatment, with smaller numbers of differential peaks between the same subsets from different treatment conditions (Extended Data Fig. 7c). Notably, among all treatment comparisons, AC484-treated TIM-3^+^ samples gained the most significant accessible peaks compared with untreated TIM-3^+^ samples and anti-PD-1-treated TIM-3^+^ samples (Extended Data Fig. 7c). We performed unsupervised clustering of differential open chromatin regions (OCRs) and defined a set of four differentiation state-specific or condition-specific modules (Fig. 5d, left, and Supplementary Table 8). For each cluster, OCRs identified using ATAC–seq closely correlated with the expression of adjacent genes in RNA-seq (Fig. 5d, right). As expected, we identified modules that defined the TIM-3^+^ terminal effector and exhausted CD8^+^ T cells (M1) and SLAMF6^+^ progenitor CD8^+^ T cells (M4) detected in each treatment (Fig. 5d). We also identified OCR modules that showed distinct patterns between treatment conditions. Specifically, M2-associated chromatin accessibility and expression of the exhaustion-related genes *Tox*, *Itgav* and *Epas1* were reduced in the AC484-treated progenitor and terminal subsets (Fig. 5d,e). Notably, the M3 module, which contains effector and memory genes, including *Gzma*, *Il7r*, *Sell* and *Tcf7*, was enriched in AC484-treated conditions relative to untreated and anti-PD-1-treated samples (Fig. 5d,e, Extended Data Fig. 7d,e and Supplementary Tables 8 and 9). These features suggest that AC484 not only induces an exaggerated T cell effector phenotype but also reduces the expression of exhaustion-associated transcripts (*Tox*) and increases the expression of transcripts associated with memory (*Il7r*, *Sell*, *Lef1* and *Tcf7*). Differential gene expression analysis revealed enrichment of memory and effector gene signatures and a reduction in exhaustion-associated genes in TIM-3^+^ T cells from AC484-treated mice relative to anti-PD-1-treated mice (Fig. 5f and Supplementary Table 9). Programmes associated with JAK–STAT signalling, including the IFNγ–IFNα response, inflammatory responses, IL-6–JAK–STAT3 signalling and IL-2–STAT5 signalling, were among the top enriched signatures from both datasets (Fig. 5g,h), a result consistent with PTPN2/N1 inhibition leading to increased signalling through JAK–STAT pathways. Recent work has shown that the combination of IL-2 and anti-PD-1 induces a specific T cell phenotype that is also associated with increased effector and memory-like capacity and a reduction in exhaustion-associated gene programmes through STAT5 activation^37,38^. To compare the epigenetic and transcriptional state induced by PTPN2/N1 inhibition to this phenotype, we looked for enrichment of transcription factor (TF) motifs and gene signatures associated with IL-2–STAT5 signalling. Unbiased TF motif enrichment of differential OCRs revealed a significant enrichment in TF motifs, including those for BATF, JUNB, FOS, STAT3, STAT1 and STAT5 (Fig. 5i). Overall, nine of the top ten most differentially enriched motifs in both comparisons were identical to those previously reported^38^ to be enriched in orthogonal IL-2-treated T cells (Fig. 5i). Finally, we mined our data for enrichment of gene sets derived from effector T cells treated with a combination IL-2 and anti-PD-L1 or with anti-PD-L1 alone, as previously described^37^. We found a highly significant enrichment of the combination IL-2 and anti-PD-L1 signature in AC484-treated TIM-3^+^ T cells and an enrichment for the anti-PD-L1 treated gene signature in anti-PD-1-treated TIM-3^+^ T cells^37^ (Fig. 5j and Extended Data Fig. 7f). Thus, by enhancing JAK–STAT signalling, AC484 treatment causes transcriptional and epigenetic reprogramming of effector CD8^+^ T cells that promotes effector function, reduces T cell exhaustion and phenocopies treatment with anti-PD-L1 and IL-2.

## AC484 increases T cell effector function and fitness

**Fig. 6:**
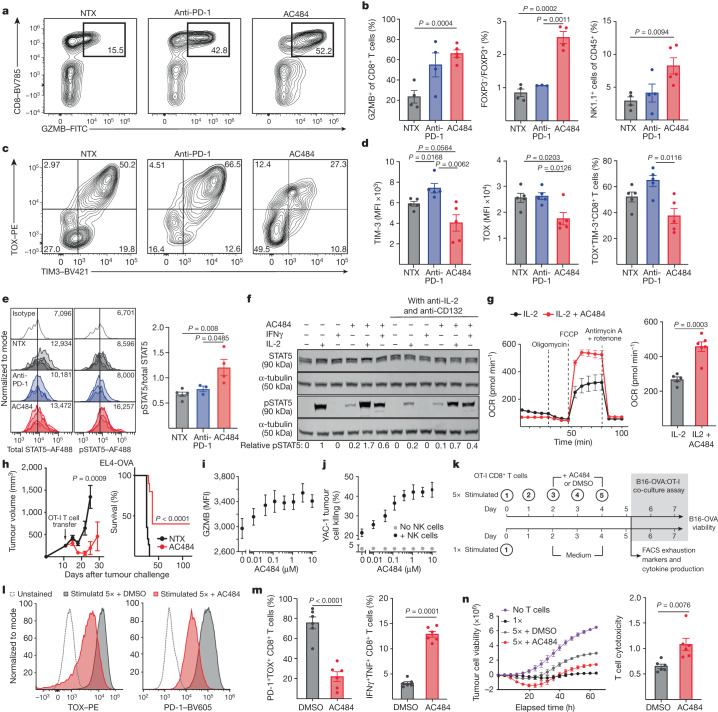
AC484 induces potent NK and T effector cells. **a**–**e**, TIL analysis from untreated, anti-PD-1-treated or AC484-treated B16 tumours. **a**, Representative GZMB and CD8 staining on TCRb^+^ cells. **b**, Quantifications of flow cytometry analyses showing per cent of GZMB^+^ CD8^+^ T cells (left), ratio of FOXP3^−^ CD4^+^ T cells to FOXP3^+^ CD4^+^ T cells (centre) and per cent of NK1.1^+^ cells among CD45^+^ cells (right). **c**, Representative CD8^+^ T cell TOX and TIM-3 staining. **d**, Quantification of TIM-3 and TOX MFI and percentage of TOX^+^TIM-3^+^ CD8^+^ T cells as in **c**. **e**, Histograms of total STAT5 and pSTAT5 staining (left) and relative pSTAT5/STAT5 levels in B16 TILs (right). **f**, pSTAT5 and STAT5 expression in naive T cells treated with IL-2 (100 ng ml^−1^), IFNγ (1 ng ml^−1^), AC484 (20 μM), AC484 and IL-2, or AC484 and IFNγ in vitro with or without anti-IL-2 (2 μg ml^−1^) and anti-CD132 (36 μg ml^−1^). **g**, Oxygen consumption rate over time (left) and mean oxygen consumption rate (right) in naive T cells stimulated with anti-CD3/CD28. FCCP, carbonyl cyanide p-trifluoromethoxyphenylhydrazone. **h**, Tumour growth (left) and survival (right) of EL4-OVA tumour-bearing mice adoptively transferred with OT-I CD8^+^ T cells expanded in vitro in IL-2 (10 ng ml^−1^) with or without AC484 (100 nM). **i**, GZMB MFI of primary mouse NK cells co-cultured with YAC-1 tumour cells with or without AC484 in vitro (*n* = 8). **j**, NK-mediated YAC-1 tumour cell killing with or without AC484 (*n* = 6). **k**, Experimental design of in vitro chronic antigen stimulation assay on primary mouse CD8^+^ T cells. **l**, TOX and PD-1 staining on chronically stimulated CD8^+^ T cells. **m**, Per cent of chronically stimulated CD8^+^ T cells expressing PD-1 and TOX (left) and IFNγ and TNF (right). **n**, B16-OVA tumour cell viability alone (purple) or co-cultured with acutely (black) or chronically stimulated OT-I CD8^+^ T cells treated with AC484 (red) or DMSO (grey). Relative cytotoxicity between AC484-treated or DMSO-treated chronically stimulated CD8^+^ T cells, normalized to acutely (1×) stimulated T cells (right). Source data

**Extended Data Fig. 8:**
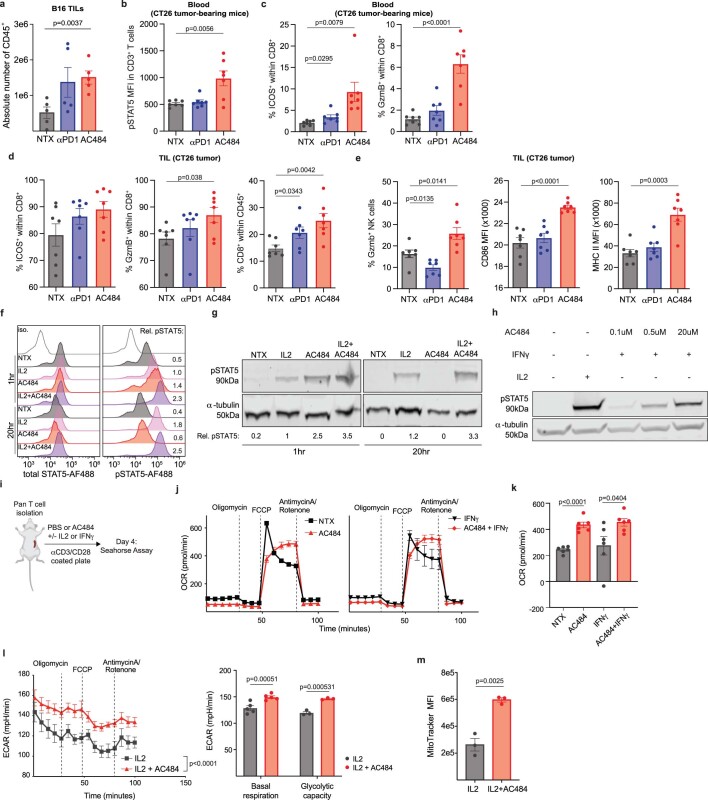
AC484 increases immune infiltration into tumors and increases STAT5 signaling and the metabolic fitness of T cells. **a**, Bar plot showing quantification of total CD45^+^ cells from flow cytometry analysis of tumour-infiltrating lymphocytes from untreated B16 tumours, or B16 tumours treated with AC484 or anti-PD-1. **b**-**e**, CT26 tumour bearing animals were treated for 8 days with AC484 starting after tumour size-match at day 7. **b**, MFI of pSTAT5 in whole blood CD3^+^ T cells. **c**, Frequency of ICOS-expressing and GZMB-expressing CD8^+^ T cells in blood. **d**, Frequency of ICOS-expressing and GZMB-expressing CD8^+^ T cells as well as frequency of CD8^+^ T cells among CD45^+^ immune cells in tumours. **e**, Frequency of GZMB-expressing NK cells and CD86 and MHC I expression on macrophages in tumours. **f**-**g**, Murine splenic CD8^+^ T cells were stimulated with plate-bound anti-CD3 (5 µg mL^−1^) and soluble anti-CD28 (2 µg mL^−1^), expanded in IL-2, and then treated with IL-2 (100 ng mL^−1^), AC484 (20 μM), or both for 1 or 20 h. **f**, Flow cytometry histograms of total STAT5 and pSTAT5 staining and relative pSTAT5 quantification of stimulated T cells. **g**, Western blot for and relative quantification (normalized to loading control) of pSTAT5 of stimulated T cells. **h**, Western blot for pSTAT5 ± IFNγ, ± IL-2, and increasing dose of AC484 (0.1 μM, 0.5 μM, 20 μM). **i**, Schematic representation of experimental design for the Seahorse assay on primary mouse T cells treated with the indicated cytokines ± AC484; figure created with BioRender.com. **j**, Oxygen consumption rate over time via the Seahorse assay using naïve T cells stimulated with plate-bound anti-CD3 (1 μg mL^−1^), soluble anti-CD28 (2 μg mL^−1^), and no cytokine (left) or IFNγ (right), treated with or without AC484 for 72 h. Oxidative phosphorylation profile was characterized by measuring OCR before and after injections of oligomycin (1.5 μM), FCCP (1 μM) and antimycin A and rotenone (0.5 μM). **k**, Quantification of mean OCR. **l**, Quantification of extracellular acidification rate (ECAR) over time (left) and mean ECAR (right) via the Seahorse assay using naïve T cells stimulated with plate-bound anti-CD3 (1 μg mL^−1^), soluble anti-CD28 (2 μg mL^−1^), IL-2 (100 ng mL^−1^), and treated with or without AC484 for 72 h. ECAR was measured before and after injections of oligomycin (1.5 μM), FCCP (1 μM) and antimycin A and rotenone (0.5 μM). **m**, Bar plot quantifying Mitotracker dye staining of naïve T cells stimulated with plate-bound anti-CD3 (1 μg mL^−1^), soluble anti-CD28 (2 μg mL^−1^), and IL-2 (100 ng mL^−1^) ± AC484 for 96 h. Source data

We next performed flow cytometry profiling of CD45^+^ TILs from untreated, anti-PD-1-treated or AC484-treated B16 tumours and CT26 tumours. We confirmed that AC484 led to an increase in overall CD45^+^ immune cell infiltration, a relative depletion of regulatory T cells, an increase in the number of infiltrating NK cells and an increase in the percentage of NK cells and CD8^+^ T cells expressing GZMB (Fig. 6a,b and Extended Data Fig. 8a–e). Consistent with our epigenetic and transcriptional profiling data, CD8^+^ T cells from AC484-treated tumours had lower TIM-3 and TOX expression and an overall reduction in the frequency of TIM-3^+^ TOX^+^ cells compared with untreated mice and anti-PD-1-treated mice (Fig. 6c,d). Finally, we isolated TILs from B16 tumours and measured phosphorylated STAT5 (pSTAT5) directly ex vivo by flow cytometry. AC484-treated but not anti-PD-1-treated mice showed a significant increase in pSTAT5 MFI compared with untreated controls (Fig. 6e). Thus, we confirmed at the protein level that systemic inhibition of PTPN2/N1 induces activation of the STAT5 pathway and a reduction in markers associated with T cell exhaustion.

Increased pSTAT5 in tumour-infiltrating T cells is surprising because the cytokines that typically drive STAT5 activation (IL-2, IL-7 and IL-15) are generally scarce in the immunosuppressive TME, and we wondered whether PTPN2/N1 inhibition causes the activation of STAT5 through cytokine-independent tonic JAK1 and JAK3 signalling. To test this, we deprived in vitro expanded CD8^+^ T cells of their supplemental IL-2 for 24 h and then treated them with IL-2, AC484 or a combination of both. AC484 augmented IL-2-induced pSTAT5, whereas cells treated with only AC484 did not show increased pSTAT5 levels. This result indicates that AC484 does not cause STAT5 activation in the absence of cytokine signalling (Extended Data Fig. 8f,g). Thus, it is possible that AC484 sensitizes T cells to low levels of IL-2 or IL-15 in the TME, or that other more abundant inflammatory cytokines provide the upstream signal for STAT5 activation. IFNγ is produced at high levels by terminal effector CD8^+^ T cells, but does not normally result in STAT5 activation. Notably, the combination of AC484 and IFNγ treatment induced pSTAT5 in CD8^+^ T cells in vitro (Fig. 6f). This result was AC484 dose dependent and occurred even when we blocked common γ-chain receptor signalling, which is required for IL-2 and IL-15 signalling (Fig. 6f and Extended Data Fig. 8h). Although these data do not prove that IFNγ signalling is responsible for the activation of STAT5 in vivo, they suggest that PTPN2/N1 inhibition can result in non-canonical STAT5 activation through IFNγ signalling, and this may contribute to STAT5 activation in the TME.

After activation, metabolic fitness can influence T cell function, and previous work has shown that JAK–STAT pathway activation can increase T cell metabolic function^39,40^. Thus, we assessed the impact of AC484 on the metabolic function of T cells using the Seahorse assay (Extended Data Fig. 8i). Compared with vehicle treatment, AC484 increased both maximal oxygen consumption rate and extracellular acidification rate in activated primary mouse T cells (Fig. 6g and Extended Data Fig. 8j–l), which suggested that there was enhanced activity of both mitochondrial and glycolytic metabolism. We also observed an increase in total mitochondrial content by MitoTracker dye staining in AC484-treated primary T cells (Extended Data Fig. 8m). We next evaluated whether the epigenetic and metabolic changes induced by transient AC484 treatment in vitro would durably improve their function and persistence in vivo. We activated SIINFEKL-specific OT-I transgenic CD8^+^ T cells treated with AC484 or vehicle control for 4 days, washed out the drug and transferred the T cells into mice bearing EL4-OVA tumours. Only the recipients of T cells treated with AC484 were able to suppress EL4-OVA tumour growth, and four of ten mice had complete responses (Fig. 6h). Thus, treatment with AC484 results in epigenetic and metabolic changes associated with improved CD8^+^ T cell effector function that persist even after removal of the drug.

**Extended Data Fig. 9:**
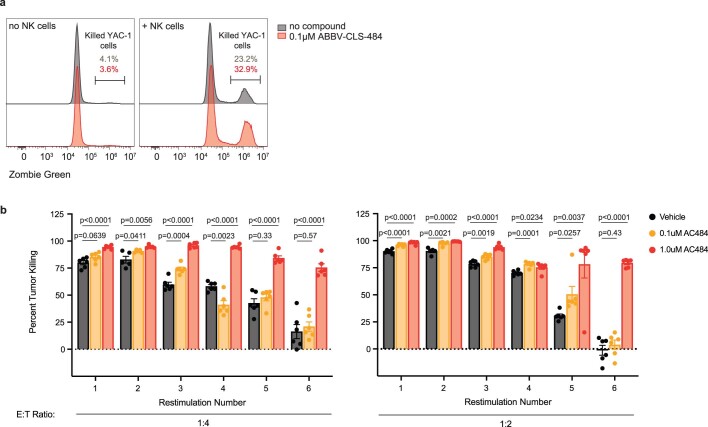
AC484 increases NK and T cell cytotoxicity in vitro. **a**, Representative histograms of killed (Zombie Green^+^) Yac-1 tumour cells by primary splenic mouse NK cells ± AC484 in vitro. **b**, Percent redirected killing of A375 melanoma cells expressing membrane-bound anti-CD3 and CD80 by primary human T cells following serial in vitro co-cultures at effector:target ratios of 1:2 or 1:4 ± AC484 (0, 0.1, or 1.0 μM) (n = 5-6). Source data

Our phenotypic profiling data suggested that AC484 treatment enhances the effector functions of CD8^+^ T cells and NK cells, and reduces CD8^+^ T cell exhaustion. To confirm that AC484 treatment has a functional impact on the cytotoxicity and persistence of these populations, we performed in vitro cytotoxicity assays. First, we cultured primary mouse NK cells with target YAC-1 tumour cells. We found that AC484 dose-dependently increased NK expression of GZMB and enhanced killing of YAC-1 cells (Fig. 6i,j and Extended Data Fig. 9a).

To functionally assess the effect of AC484 on CD8^+^ T cell cytotoxicity and exhaustion, we repetitively stimulated CD8^+^ T cells isolated from OT-I transgenic mice with SIINFEKL for 5 days with or without AC484 treatment, and assessed their phenotype and function (Fig. 6k). This assay elicits an exhausted and dysfunctional T cell phenotype characterized by high expression of the transcription factor TOX and PD-1 (refs. ^36,41^) (Fig. 6l, grey). Notably, AC484 treatment during chronic antigen stimulation significantly reduced TOX and PD-1 expression levels and the frequency of PD-1^+^TOX^+^ CD8^+^ T cells (Fig. 6l,m). Furthermore, AC484 induced a threefold increase in T cells that produced both IFNγ and TNF, a hallmark of multifunctionality^42^ (Fig. 6m). Finally, we evaluated the function of these chronically stimulated cells in a co-culture killing assay with B16-OVA tumour cells. Compared with acutely activated T cells, which had been stimulated once with SIINFEKL peptide, chronically stimulated T cells had reduced cytotoxic activity (Fig. 6n, black compared with grey). However, T cells that had been chronically stimulated and treated with AC484 showed significantly increased cytotoxic activity (Fig. 6n, red). This finding was confirmed in a human T cell redirected killing assay^43^, using T cells that had been repetitively co-cultured with target cells in the presence of AC484 or vehicle (Extended Data Fig. 9b). Thus, inhibition of PTPN2/N1 with AC484 during chronic antigen exposure significantly reduces the expression of exhaustion-related genes in T cells and improves both the cytokine production and cytotoxic activity of CD8^+^ T cells and NK cells.

## Conclusions

Here we described the discovery and preclinical characterization of AC484, a new first-in-class, orally bioavailable active site inhibitor of PTPN2/N1. AC484 treatment potently sensitized cancer cells to IFNγ and enhanced the activation and effector functions of T cells and NK cells. With AC484, we showed that oral administration of a PTPN2/N1 systemic inhibitor is a well-tolerated and effective cancer immunotherapy in multiple preclinical models, including those that are resistant to PD-1 blockade.

We showed that AC484 enhances the anti-tumour effects of IFNγ and has broad phenotypic and functional effects on several subsets of tumour-infiltrating immune cells, including macrophages, dendritic cells, T cells and NK cells. The activity across many immune cells led to AC484 efficacy in settings in which T cell immunity is insufficient to control tumour growth, such as in JAK1-deficient or MHC class I-deficient tumours, and the surveillance of metastasis. This result suggests that AC484 has the potential to overcome tumour immune-evasion mechanisms, such as mutations in genes encoding β_2_-microglobulin, HLA and JAK1 and JAK2 (refs. ^44–47^). The broad immune-activating effects are probably due to the activation of multiple JAK–STAT signalling pathways in immune effector cells, including IL-2–STAT5. Cytokine signalling through these pathways has been linked to enhanced T cell metabolic fitness^39,40^, which is consistent with the observed AC484-enhanced mitochondrial content and function in T cells.

Previous work has indicated that PTPN2 deficiency may increase T cell exhaustion or dysfunction owing to heightened chronic antigen and IFN sensing^5,48^. Here we showed that PTPN2/N1 inhibition reduces exhaustion and increases T cell memory signatures, as we observed reduced expression of PD-1, TOX and TIM-3 in chronically antigen-stimulated or tumour-infiltrating CD8^+^ T cells. Moreover, inhibition of PTPN2/N1 and activation of JAK–STAT signalling in T cells caused epigenetic, transcriptional and metabolic changes that result in a distinct T cell transcriptional and functional state that is both highly cytotoxic and more persistent and polyfunctional. Notably, the transcriptional and epigenetic programmes we observed in AC484-treated T cells are highly similar to those identified in T cells treated with a combination of anti-PD-L1 and IL-2, which suggests that AC484 may enhance T cell functionality through a similar mechanism. The large number of important immune signalling pathways that are enhanced by inhibition of PTPN2/N1, including IFN sensing, TCR signalling and IL-2, IL-7 and IL-15 signalling suggest that AC484 can work to stimulate immune responses through a variety of cellular and molecular mechanisms to exert its anti-tumour activity. Based on this compelling pharmacology and safety profile in preclinical toxicology studies, AC484 is currently being evaluated in Phase I clinical studies as monotherapy and in combination with anti-PD-1 in solid tumours (ClinicalTrials.gov identifier NCT04777994).

## Methods

### Compound

AC484 was synthesized using previously reported methods^49^. AC484 characterization: zwitterion: ^1^H NMR (501 MHz, DMSO-*d*_6_) *δ* ppm 9.22 (s, 1H), 8.39 (br s, 2H), 6.47 (s, 1H), 3.93 (s, 2H), 3.43–3.35 (m, 1 H), 3.09 (dd, *J* = 16.0, 5.6 Hz, 1H), 3.03 (td, *J* = 7.0, 2.1 Hz, 2H), 2.81 (dt, *J* = 17.2, 4.7 Hz, 1H), 2.73 (ddd, *J* = 17.0, 11.0, 5.3 Hz, 1H), 2.56–2.51 (m, 1H), 2.20–2.13 (m, 1H), 1.68 (ddt, *J* = 17.2, 13.1, 6.0 Hz, 2H), 1.50 (dt, *J* = 9.7, 6.8 Hz, 2H), 0.92 (d, J–6.6 Hz, 6H); MS (ESI^−^) *m/z* 384 [M-H]; Na-salt: ^1^H NMR (600 MHz, DMSO-*d*_6_) δ 6.42 (s, 1H), 3.96 (s, 2H), 2.84 (dd, *J* = 16.2, 5.0 Hz, 1H), 2.78 (m, 1H), 2.73 (dt, *J* = 17.2, 5.0 Hz, 1H), 2.61 (m, 3H), 2.21 (dd, *J* = 16.0, 8.6 Hz, 1H), 1.91 (m, 1H), 1.63 (nonet, *J* = 6.8 Hz, 1H), 1.43 (m, 1H), 1.32 (q, *J* = 7.4 Hz, 2H), 0.87 (d, *J* = 6.6 Hz, 6H); ^13^C{^1^H} NMR (101 MHz, DMSO-*d*_6_) δ 172.7, 160.3, 157.9, 154.4, 138.1, 114.2, 111.5, 57.7, 52.8, 45.0, 39.3, 28.8, 28.7, 27.7, 26.1, 23.12, 23.09; ^19^F NMR (376 MHz, DMSO- *d*_6_) δ –122.7; HRMS (ESI/Orbitrap) *m/z*: [M–H]^–^ Calcd for C_17_H_23_FN_3_O_4_S 384.1393; found 384.1396; ${[\alpha ]}_{D}^{24}$ = +40.4 (*c* = 1.0, DMSO); Mp: 234–237 °C (dec.).

A-650 was synthesized using previously reported methods^50^ (Ex 103). A-650 characterization: ^1^Η NMR (400 MHz, DMSO-*d*_*6*_) δ ppm 10.04 (s, 1Η), 7.60 (d, J = 8.9 Hz, 1Η), 7.18 (dd, J = 8.9, 2.2 Hz,1Η), 6.97 (s, 1Η), 6.95 (br s, 1Η), 4.42 (s, 2Η), 3.05 (d, J = 6.8 Hz, 2Η), 1.12-1.04 (m, 1Η), 0.56–0.45 (m, 2Η), 0.32–0.24 (m, 2Η); MS (ESI^−^) *m/z* 364 [M-H]^−^.

### Crystallography

#### Protein expression and purification

Human PTPN2 (UniProtKB database accession number P17706) was generated by cloning the *PTPN2* gene encoding Met1–Asn314 into a pET28b vector. A coding sequence for an 8× histidine tag was added to the 3′ of the construct sequence. This plasmid was transformed into BL21 (DE3) *Escherichia coli* cells and grown at 37 °C in Terrific Broth containing 100 μg ml^–1^ kanamycin. At an OD_600_ of 1.0, PTPN2 expression was induced using 1 mM IPTG. Cells were collected by centrifuge after overnight growth at 18 °C.

Cell pellets were resuspended in lysis buffer containing 50 mM Tris-HCl (pH 8.0), 500 mM NaCl, 1 mM TCEP, 10 mM imidazole, 0.1% Triton X-100 and complete EDTA-free protease inhibitor, and lysed using a microfluidizer, followed by ultracentrifugation at 20,000*g*. The supernatant was loaded onto a HisTrap HP Nickel chelating column in 50 mM Tris-HCl (pH 8.0), 10 mM imidazole, 500 mM NaCl and 1 mM TCEP, and protein was eluted with the addition of 300 mM imidazole. Fractions containing PTPN2 were pooled and concentrated, then loaded onto a HiLoad 26/600 Superdex 200 PG column equilibrated with 25 mM Tris (pH 8.0), 250 mM NaCl and 2 mM dithiothreitol. The protein was concentrated to 20 mg ml^–1^ for use in crystallization.

#### Protein crystallization and X-ray crystallography

Purified PTPN2 was crystallized in the presence of a displaceable active-site inhibitor compound using the sitting drop vapour diffusion technique at 290 K with precipitant reservoir of 25% PEG 3350, 0.2 M (NH_4_)_2_SO_4_ and 0.1 M Tris pH 8.5. The complex with AC484 was obtained by transferring the crystal to a solution of 3 mM compound in the reservoir with 15% of ethylene glycol and soaking the crystal for 6 h. The crystal was collected in a loop mount and preserved by plunging into liquid nitrogen. X-ray diffraction data were collected at 100 K on the IMCA-CAT beamline 17-ID of the Advanced Photon Source at Argonne National Laboratory, USA. The structure was solved by molecular replacement using the program Phaser and the Protein Data Bank entry 1L8K as a search model. The structure was rebuilt with iterative rounds of refinement using COOT and BUSTER software (Global Phasing). Figures were generated using PyMol (Schrodinger).

### Mobility shift assay

Full-length recombinant PTPN2 or PTPN1 were incubated with increasing concentrations of AC484 or A-650 for 10 min at room temperature. Oregon green-labelled insulin receptor probe (sequence: (OG488)-DPEG2-T-R-D-I-(PY)-E-T-D-Y-Y-R-K-K-CONH2) was added and incubated for another 10 min. Finally, the reaction was quenched with water and a potent known PTPN2/PTPN1 inhibitor. Fluorescent signals of product and probe were subsequently measured on a LabChip EZ Reader (Perkin-Elmer).

### Selectivity analyses

Wide-range phosphatase, kinase and preclinical safety pharmacology off-target activity screens were conducted at Eurofins (Extended Data Table 5 and Supplementary Tables 1 and 2). A small phosphatase panel was developed at AbbVie to assess IC_50_ values on select phosphatases. The following enzymes used in the phosphatase selectivity panel were purchased from Eurofins: PTPN1 (PTP1B; 14–621), PTPN2 (TC-PTP; 14–646), PTPN9 (14–592), SHP1 (14–591) and SHP2 (14–622). The catalytic activity of the phosphatase was monitored using the small-molecule substrate DiFMUP (ThermoFisher) in a fluorescence assay format. In brief, the phosphatase reactions were performed at room temperature in the following assay buffer: 50 mM HEPES, pH 7.2, 100 mM NaCl, 1 mM EDTA, 0.005% Tween-20 and 5 mM TCEP. Each phosphatase was incubated with increasing concentrations of AC484 for 5 min, after which the substrate DiFMUP was added and incubated at 25 °C for 30 min. The final concentration of the enzyme and substrate was maintained at 0.5 nM and 5 μM, respectively, in all the assays. The reaction was then quenched by the addition of a 100 μM solution of bpV(Phen) (Enzo Life Sciences). The fluorescence signal was monitored using an Envision microplate reader (Perkin-Elmer) using excitation and emission wavelengths of 340 nm and 450 nm, respectively. The dose–response curves for the inhibitors were analysed using normalized IC_50_ regression curve fitting with control-based normalization.

### B16 pSTAT fluorescence resonance energy transfer assays

To assess pSTAT1, B16 cells were pre-incubated with AC484 for 3 h followed by stimulation with 100 ng ml^−1^ IFNγ for 10 min. Next, 3 μM staurosporine was added and incubated for 1 h. Cells were lysed in 50 μl of 1× lysis buffer (kit provided) and assayed for pSTAT1 using a phospho-STAT1 (Tyr701) Cellular HTRF kit (Cisbio). Data are reported as the fold change of the HTRF ratio with the PTPN2 inhibitor over DMSO control.

### Mouse and human unbound AC484 fraction in plasma and mouse pharmacokinetics

The unbound fraction of AC484 in human and mouse plasma (fu,p) was determined by equilibrium dialysis using a 96-well HT Dialysis apparatus with dialysis membrane strips (MWCO 12–14 kDa) according to previously reported methods^51^.

Unbound mouse in vivo clearance was determined from a low-dose pharmacokinetics study described below, with determined clearance being divided by the fraction unbound in mouse plasma.

AC484 pharmacokinetics were evaluated following single intravenous or oral doses to groups of male CD1 mice (Charles River Laboratories). AC484 was administered as a solution in DMSO: dextrose 5% in water (5:95, v/v) for intravenous dosing or as a solution in 0.5% HPMC for oral dosing (10 ml kg^–1^ dose volume for both). Groups of three mice received a single 1 mg kg^–1^ intravenous dose, administered as a slow bolus in a penile vein under isoflurane anaesthetic. An additional group of 3 mice received a 10 mg kg^–1^ oral dose, administered by gavage. EDTA-preserved blood samples were obtained from each animal at 0.1 (intravenous only), 0.25, 0.5, 1, 3, 6, 9, 12 and 24 h after dosing. Plasma was separated from the blood samples by centrifugation and stored frozen (<15 °C) until mass spectrometry analysis.

### Dose-escalating mouse pharmacokinetics

AC484 pharmacokinetics were evaluated following single oral doses to groups of male C57Bl/6N mice (Charles River Laboratories). Mice were permitted free access to food and water. AC484 was administered as a solution in ethanol: PEG-400: Phosal 50 PG (Lipoid, LLC) (10:30:60, w/w) to mice (10 ml kg^–1^ dose volume). Groups of mice (3 per group) received a single 3, 10, 30 or 100 mg kg^–1^ oral dose by gavage. EDTA-preserved blood samples were obtained at 0.25, 0.5, 1, 3, 6, 9, 12 and 24 h after the dose in each mouse. Plasma was analysed as described for the low-dose study above.

### Cell lines

B16 and B16-GM-CSF (GVAX) lines were received as a gift from G. Dranoff (Dana-Farber Cancer Institute). The KPC pancreatic cancer cell line was a gift from A. Maitra (MD Anderson Cancer Center) and S. Dougan (Dana-Farber Cancer Institute). MC38 colon carcinoma cell lines were obtained from the National Cancer Institute or as a gift from A. Sharpe (Harvard Medical School). A375 melanoma cells, CT26.WT colon carcinoma (referred to as CT26), EMT-6 and 4T1 cells were purchased from the American Type Culture Collection (ATCC). All cell lines were tested for mycoplasma and cultured in DMEM or RPMI (Gibco) supplemented with 10% FBS; at the Broad Institute, cell lines were also cultured with antibiotics. Nuclight-Red-expressing B16-OVA cells generated at AbbVie using Incucyte Nuclight Red Lentivirus (Sartorius) were cultured in DMEM (Gibco)/10% FBS-HI. All experiments were performed using cells below passage 16.

### Generation and validation of CRISPR-mediated knockout cell lines

Cells were transiently transfected with pX459_Cas9_sgRNA (Addgene, 62988) containing non-targeting control, *Ptpn1*, *B2m* or *Jak1* single guide RNA (sgRNA) sequences with Lipofectamine transfection reagent (ThermoFisher Scientific, L3000015). B16 *Ptpn2*-null cells used were generated as previously described^3^. Transfected populations were selected in antibiotic for 2–4 days, and bulk transfectant populations were used for subsequent experiments. All cell lines were validated for knockout efficiency by flow cytometry, western blotting or amplicon sequencing of targeted loci. For amplicon sequencing-based validation of knockout efficiency, custom PCR primers were designed surrounding target sites for the respective *Ptpn1* and *Ptpn2* sgRNAs. Genomic DNA was isolated from edited cells using a QIAmp DNA mini kit (Qiagen) and targeted loci were PCR-amplified and analysed by Illumina sequencing.

### CRISPR sgRNA sequences

The gene name, sgRNA number and sequence were as follows.

Control sgRNA1: GCGAGGTATTCGGCTCCGCG; control sgRNA2: GCTTTCACGGAGGTTCGACG; control sgRNA3: ATGTTGCAGTTCGGCTCGAT.

*Ptpn1* sgRNA1: GGAAAGAAGCCGGTGACTCG; *Ptpn1* sgRNA2: GGTGCAATTTAATCCGACTG; *Ptpn1* sgRNA3: GCCCTTTACCAAACACATGT; *Ptpn1* sgRNA4: GCCCTTTACCAAACACATGTNGG.

*Ptpn2* sgRNA1: TCATTCACAGAACAGAGTGA; *Ptpn2* sgRNA2: AAGGCAGTGGTTTCTTACGA; *Ptpn2* sgRNA3: GGAGTGGAAACGCTTCATCG; *Ptpn2* sgRNA4: ACCATTTCTCTGTCATCCGTNGG.

*Jak1* sgRNA1: GTACTCACTGCACTCCTGGG; *B2m* sgRNA1: AGTATACTCACGCCACCCAC.

### In vitro growth kinetics of tumour cells

Control, *Ptpn2* or *Ptpn1* single deletion, or *Ptpn2/n1* dual deletion B16 cells were seeded at a density of 10,000 cells per well in a 96-well plate and incubated in an IncuCyte S3 Live-Cell Analysis system (Sartorius) at 37 °C and scanned at ×10 magnification every 3 h for 100 h. *Ptpn2/n1* dual deletion B16 cells were incubated in complete DMEM medium, with IFNγ (10 ng ml^−1^, Peprotech), and control cells were incubated with IFNγ (10 ng ml^−1^), AC484 (1 or 0.1 µM), or both in complete DMEM medium. In vitro growth was assessed by well confluency as calculated using the IncuCyte S3 Live-Cell Analysis system Basic Analyzer. Data were analysed using Graph-Pad Prism (v.9.0).

For in vitro dose-escalation studies in tumour cells, B16 mouse melanoma cells were seeded in DMEM with 10% FBS and treated with 0.5 ng ml^−1^ IFNγ or medium as a control the following day. AC484 was added and confluence imaged every 6 h in an IncuCyte S3 Live-Cell Analysis system (Satorius) for 5 days. Confluence values were obtained when the ‘no compound/no IFNγ’ control reached a confluence of >95%, and the per cent growth inhibition of AC484 at the indicated concentration was calculated relative to the ‘no compound/with IFNγ’ control. *Ptpn2* and *Ptpn1* knockout B16 cells for these experiments were generated by the Genome Engineering and iPSC Center at Washington University in St Louis using CRISPR technology.

### Transcriptional profiling by RNA-seq analysis

Control and *Ptpn2/n1* dual deletion B16 cells were seeded at a density of 250,000 cells per well in a 12-well plate and stimulated with IFNγ (10 ng ml^−1^), AC484 (10 µM) or both. After 24 h, cell pellets were collected for transcriptional profiling. RNA was isolated from cell pellets using a Qiagen RNeasy Mini kit according to the manufacturer’s instructions. First-strand Illumina-barcoded libraries were generated using a NEB RNA Ultra Directional kit according to the manufacturer’s instructions, a NEBNext Poly(A) mRNA Magnetic Isolation Module and a 12-cycle PCR enrichment step. Libraries were sequenced on an Illumina NextSeq 500 instrument using paired-end 37-bp reads.

### Analysis of gene expression from RNA-seq data

Read data were adapter and quality trimmed using Trimmomatic (v.0.36)^52^. Trimmed reads were quantified by pseudoalignment to mm10 using Kallisto (v.0.46.0)^53^. RNA-seq counts were quantified using the program tximport. A variance stabilizing transformation was applied to the counts matrix, and differentially expressed genes were quantified using the DESeq2 R package^54^. GSEA was performed using GSEAPreranked^18,55^.

### B16-OVA antigen presentation and OT-1 tumour cell killing assays

To assess antigen presentation, B16-OVA cells generated at AbbVie were cultured in DMEM with 10% FBS overnight and treated thereafter with AC484 with or without 1 ng ml^–1^ IFNγ. Cells were cold-lifted on ice, stained using a Zombie UV Fixable Viability kit (BioLegend) for dead-cell exclusion, surface stained with anti-PD-L1 and anti-H-2Kb bound to SIINFEKL, and analysed on a BD LSR Fortessa X-20 (BD Biosciences).

For the tumour killing assay, CD8^+^ T cells were negatively isolated using a mouse CD8a^+^ T cell isolation kit (Miltenyi Biotec) from transgenic OT-1 mouse splenocytes (Jackson Laboratories) and stimulated with 2 μg ml^–1^ plate-bound anti-CD3 antibody (BioLegend, clone 145-2C11) and 2 μg ml^–1^ soluble anti-CD28 antibody (BioLegend, clone 37.51) in T/NK cell medium (RPMI 1640 supplemented with 10% FBS, 50 nM 2-ME, 100 U ml^–1^ penicillin and 100 µg ml^–1^ streptomycin) containing 10 ng ml^–1^ IL-2 for 2 days. Cells were expanded by a change of medium every 2 days for a total of 8 days. Nuclight Red-expressing B16-OVA cells generated using Incucyte Nuclight Red Lentivirus (Satorius) were incubated for 4 h and treated with AC484 and 0.5 ng ml^–1^ IFNγ. After overnight incubation, expanded mouse OT-1 T cells were added for 16–18 h at an effector to target (E:T) cell ratios of 1:4 in T/NK cell medium. T cells were decanted, and tumour cells were lifted with trypsin. Cells were stained using a Zombie Violet Fixable Viability kit (BioLegend), and the absolute live tumour cell count was analysed using a Quanteon flow cytometer (NovoCyte), gating on Nuclight Red positive, Zombie Violet low for viable tumour cells. Tumour killing is expressed relative to similarly treated wells of tumour cells containing no T cells.

### Primary mouse T cell activation and cytokine production analysis

Primary mouse T cells were negatively selected from spleens of C57BL/6N mice (Charles River Laboratories) using a MACS Pan T cell Isolation kit II, a CD4^+^ T cell Isolation kit and a CD8^+^ T Cell Isolation kit (Miltenyi Biotech). For *Ptpn2* and *Ptpn1* knockout, CD8^+^ T cells were negatively selected using an EasySep Mouse CD8^+^ T Cell Isolation kit (StemCell Technologies). Cells for knockout were cultured in T/NK cell medium with anti-CD3 and anti-CD28 antibodies for 48 h before CRISPR RNP with Cas9 and sgRNA (IDT). Knockout cells were rested for 48 h before inhibitor treatment and activation. All primary T cells were cultured in T/NK cell medium and treated with AC484 and stimulated with mouse anti-CD3/anti-CD28 T-Activator Dynabeads (Gibco). Pan T cells were stimulated at a ratio of 1 bead to 5 cells, whereas the CD4^+^, CD8^+^ or knockout T cells were stimulated at a ratio of 1 bead to 1 cell. After 48 h of incubation, cells were stained using a Zombie UV Fixable Viability kit for dead-cell exclusion followed by surface staining with CD3, CD4, CD8, B220, CD25 and CD69 surface antigens. Cells were analysed using an LSR Fortessa X-20 (BD Biosciences). Supernatants were analysed for IFNγ and TNF using MSD V-plex ELISA kits (Meso Scale Discovery) after 48 or 72 h of incubation for isolated CD4^+^ and CD8^+^ T cells or pan T cells, respectively.

### PRISM screen

A viability screen was conducted using the PRISM screening platform of 489 adherent and 278 suspension cancer cell lines. Pools of cell lines (*n* = 20–26) were thawed and plated into 384-well plates at 1,250 (adherent cell pools) or 2,000 (non-adherent cell pools) cells per well. AC484 was added at an 8 point dose with 3-fold dilutions in triplicate (with and without IFNγ at 1 or 100 ng ml^–1^) using a HP D300e Digital Dispenser, and cells were incubated for 5 days. Cells were then lysed, and cell line barcodes were detected using Luminex. Data analyses were performed using a pipeline developed by PRISM^19,56^. From this, we were able to obtain dose–response curves and log_2_(fold changes) at each individual dose. We then looked for biomarkers correlated with sensitivity to AC484. To find individual genes associated with sensitivity, we calculated the Pearson correlation between variance stabilizing transformation (vst)-normalized gene expression and response to inhibitor. Gene sets associated with response were calculated by passing this list of Pearson correlations into GSEAPreranked^18,55^.

### In vitro human whole blood studies

#### Sample collection

Human blood samples were acquired through AbbVie’s internal blood donation programme in accordance with AbbVie’s Occupational Safety and Health Administration protocols. The protocol, under which human blood samples were acquired, was approved by and is reviewed on an annual basis by WCG IRB (Puyallup, Washington). WCG IRB is in full compliance with the Good Clinical Practices as defined under the US Food and Drug Administration Regulations, US Department of Health and Human Services regulations and the International Conference on Harmonisation Guidelines. All human research participants signed informed consent forms. Blood samples from patients with cancer were acquired from Discovery Life Sciences. Data from whole blood showing occasional overt clotting after overnight culture were excluded.

#### pSTAT flow cytometry analyses

Heparinized blood samples were treated with AC484 for 3 h followed by stimulation with either IL-2 or IFNγ (both at 100 ng ml^−1^) for 1 h. Fixation and red blood cell lysis was performed with BD Phosflow Lyse/Fix buffer (BD Biosciences). Cells were subsequently permeabilized with Perm III buffer (BD Biosciences) and stained for CD45, CD3, CD14 and phospho-STAT5 following IL-2 stimulation or phospho-STAT1 following IFNγ stimulation for 2 h. Samples were run on an LSR Fortessa X-20 (BD Biosciences) and data were analysed using FlowJo (v.10.8.1) analysis software (BD Biosciences). STAT phosphorylation was measured on the basis of the MFI of pSTAT5 in CD3^+^ T cells or pSTAT1 in CD14^+^ monocytes.

#### Immune activation and functional studies

Fresh human heparinized whole blood samples were treated with AC484, followed by T cell stimulation with optimized concentrations of CytoStim (Miltenyi Biotec). After 18 h of incubation, supernatants were analysed for IL-2, IFNγ and TNF using Mesoscale Discovery V-plex kits. Fixation and red blood cell lysis was performed on the cells with BD Phosflow Lyse/Fix buffer, then surface stained for 45 min for CD45, CD3 and CD69. Cells were washed and acquired on an LSR Fortessa X-20 (BD Biosciences). The data were analysed using FlowJo (v.10) analysis software. For CXCL10 analysis, fresh heparinized whole blood samples were treated with increasing concentrations of AC484 and 100 ng ml^–1^ IFNγ for 18 h, and CXCL10 levels were measured in supernatants using a V-Plex Plus Human IP10 kit (Mesoscale Discovery).

#### Gene expression analysis

Whole blood was stimulated through the TCR with CytoStim (Miltenyi Biotec) and treated with AC484 overnight followed by PBMC isolation. Gene expression levels in PBMCs were measured using Nanostring with the nCounter immunology panel. Expression levels were calculated using the NanoStringNorm package (v.1.2.1.1.; https://cran.r-project.org/web/packages/NanoStringNorm/index.html). Differential expression was performed on adjusted counts between vehicle-treated and drug-treated samples using the limma method, with dose as the continuous covariate and blocking on donor. Multiple test correction was performed using the Benjamini–Hochberg procedure. Full results are available in Supplementary Table 4.

### Rat toxicity study with AC484

Male Sprague–Dawley rats were administered AC484 by oral gavage once daily for 4 weeks to characterize the toxicity profile. All animal use was conducted in accordance with the Guide for the Care and Use of Laboratory Animals and was approved by AbbVie’s Institutional Animal Care and Use Committee (IACUC). AC484 was formulated in 2% (w/v) hydroxypropylcellulose (HPC-SL) with 0.2% (w/v) sodium dodecyl sulfate (SDS) in water. Rats were administered either vehicle (2% (w/v) HPC-SL with 0.2% (w/v) SDS in water) or AC484 in vehicle once daily at 15 or 300 mg kg^–^^1^ per day. Ten rats were assigned to each dosing group. Six additional rats were assigned to recovery groups dosed daily with either vehicle or AC484 at 300 mg kg^–^^1^ per day for 4 weeks followed by a 4-week dose-free period. Blood was collected from the vena cava in rats anaesthetized with isoflurane at the end of the dosing and recovery phases for haematology, clinical chemistry and coagulation analysis. Surviving rats were euthanized at the end of their assigned dosing or recovery period, tissues were examined macroscopically at necropsy, and tissue samples were collected and preserved in 10% neutral-buffered formalin. Samples of femorotibial joint were decalcified before tissue processing. Tissue samples were embedded in paraffin, sectioned, stained with haematoxylin and eosin, and examined by microscopy.

### Pathway engagement

Blood samples were collected from mice on day 8 of dosing and treated with 100 ng ml^–1^ IL-2. Sample fixation and red blood cell lysis were performed using Phosflow Lyse/Fix buffer (BD Biosciences). Cells were subsequently permeabilized by adding Perm III buffer (BD Biosciences), stained with CD45, CD3 and phospho-STAT5 for 2 h, and run on an LSR-Fortessa X20 flow cytometer (BD Biosciences). The amount of STAT phosphorylation (pSTAT5) as a readout of pathway engagement is expressed as the MFI of pSTAT5 in CD3^+^ T cells.

### Animal treatment

AbbVie is committed to ensuring the humane care and use of laboratory animals in the company’s research and development programmes. All animal studies were reviewed and approved by AbbVie’s IACUC and in compliance with the NIH Guide for Care and Use of Laboratory Animals guidelines. Animal studies were conducted in an AAALAC-accredited programme where veterinary care and oversight was provided to ensure appropriate animal care. For all in vivo studies conducted at AbbVie, 8- to 12-week-old female C57BL/6N or BALB/c mice obtained from Charles Rivers Laboratories were used. The maximal tumour volume end point allowable per the IACUC protocol was 3,000 mm^3^ (the tumour volume was estimated by applying the following equation: volume = (length × width^2^) / 2). Exceeding a tumour volume of 3,000 mm^3^ or a body weight loss of animals of ≥20% warrants humane euthanasia. In most cases during the study, any animal with a tumour volume above 2,000 mm^3^ or a 15% loss in body weight was euthanized and removed from study.

All in vivo studies conducted at the Broad Institute were approved by the Broad Institute IACUC committee, and mice were housed in a specific-pathogen free facility. All in vivo studies at Calico were conducted according to protocols approved by the Calico IACUC. Six-to-eight-week-old C57BL/6J female mice were obtained from Jackson Laboratories or Charles River Laboratories. BALB/cJ mice were obtained from Jackson Laboratories or Charles River Laboratories. A colony of immune incompetent NOD.Cg-Prkdc^scid^ Il2rg^tm1Wjl^/SzJ (NSG) mice were bred at the Broad Institute. For all tumour challenges, 7-to-12-week-old, age-matched mice were used, and pre-specified end points for tumour size were adhered to as defined by the Broad IACUC, including 2.0 cm in maximum dimension for validation studies. CO_2_ inhalation was used to euthanize mice on the day of euthanasia.

### Tumour challenges and analyses of tumour growth

For subcutaneous tumour challenges, mice were inoculated in the lower flank with 6 × 10^4^ (B16, KPC or 4T1) or 1 × 10^5^ (EMT-6, MC38 or CT26) cells resuspended in Hanks balanced salt solution (HBSS, Gibco) mixed 1:1 by volume with Matrigel (Corning, BD Biosciences) on day 0. Tumours were measured every 3 days beginning on day 6 until time of death. Death was defined as the point at which a progressively growing tumour reached 2.0 cm in the longest dimension. Measurements were manually taken by collecting the longest dimension (length) and the longest perpendicular dimension (width). Tumour volume was estimated with the formula: (length × width^2^)/2. Optimal group sizes were empirically determined. Researchers were not blinded to group identity, and randomized size-matching of animal groups was done when appropriate.

For GVAX treatment in B16 tumour challenges, mice were vaccinated on days 1 and 4 with 4.0 × 10^5^ GM-CSF-secreting B16 (GVAX) cells that had been irradiated with 3,500 Gy to elicit an anti-tumour immune response. Where indicated, mice were intraperitoneally treated with rat monoclonal anti-PD-1 (Bio X Cell, clone: 29F.1A12) every 3–4 days starting from day 6 or 7 for a total of three or four doses. Mice received anti-PD-1 doses of 5 mg kg^–1^ (B16) or 10 mg kg^–1^ (KPC, 4T1, EMT-6 and CT26). AC484 was administered either twice daily at 10 mg kg^–1^ or once daily at 20 mg kg^–1^ or 100 mg kg^–1^ by oral gavage beginning on day 6 or 7 for 15 (KPC and B16) or 21 (4T1, EMT-6 and CT26) consecutive days. For depletion experiments, mice were treated with 200 μg of anti-CD8b (Bio X Cell, clone 53-5.8), 200 μg of anti-NK1.1 (Bio X Cell, clone PK136, monoclonal) or 200 μg of mouse IgG2a isotype control (Bio X Cell, clone C1.18.4, monoclonal) by intraperitoneal injection every 3 days starting from 1 day before tumour implantation and continuing until 21 days after tumour challenge.

For the B16 pulmonary metastasis model, 5 × 10^5^ B16 cells stably expressing Firefly luciferase were intravenously injected by the tail vein. AC484 was administered daily by oral gavage at 100 mg kg^–1^ beginning on day 0 for 15 consecutive days. Next, 100 μg of anti-PD-1 was administered on days 0, 3 and 6 following tumour inoculation. In vivo bioluminescent imaging was used to monitor metastatic progression on a bi-weekly basis until humane end point was reached. On imaging days, mice were anaesthetized with 2% isoflurane and intraperitoneally injected with IVISbrite d-Luciferin potassium salt (Perkin Elmer) resuspended in PBS (150 mg kg^–1^). After 10 min, ventral images were captured using an IVIS Spectrum CT In Vivo Imaging system (Perkin Elmer). Living Image software (v.4.5.5, PerkinElmer) was used to determine regions of interest (ROI) for each subject, and the bioluminescent signal was quantified within each ROI as total flux (photons s^–1^).

For the 4T1 metastasis model, 1 × 10^5^ 4T1 cells were subcutaneously implanted into the mammary fat pad. Mice were treated daily with vehicle or AC484 (20 or 60 mg kg^–1^) starting at day 7 until euthanasia. Lung metastases were analysed by inflation with 15% India ink. After intratracheal injection of India ink, lungs were collected and washed with Feket’s solution (70% ethanol, 3.7% paraformaldehyde and 0.75 M glacial acetic acid). Lungs were placed in fresh Feket’s solution overnight, and surface white tumour nodules were counted in a randomized blinded manner by a third party. For CD8-depletion and NK cell-depletion studies, naive female mice were treated with 200 μg anti-CD8a (Bio X Cell, clone 2.43), 50 µl of anti-Asialo GM1 (Wako Chemicals, polyclonal) or 200 μg IgG2b isotype control (Bio X Cell, clone LTF-2) 4 days before 4T1 tumour implant and then every 4 days for 4 additional doses before stopping. Successful depletion was consistently observed by peripheral blood FACS analysis.

### Drug preparation for in vivo administration

AC484 was synthesized at AbbVie and dissolved in DMSO and further diluted in a solvent consisting of H_2_O, PEG-40, Tween-80 and DMSO at a ratio of 70:20:5:5, respectively. AC484 was formulated in ethanol, PEG400 and Phosal-50PG at a ratio of 10:30:60, respectively, for oral dosing.

### Histology

Whole KPC tumours were fixed for 24 h in 10% neutral-buffered formalin and then transferred to 70% ethanol until processing. Fixed tissue was subsequently embedded into paraffin, sectioned and then mounted onto slides for staining against mouse CD45 and CD8 at the BWH Pathology Core. Slides were imaged on an Aperio Versa 200 and analysed using QuPath (v.0.3.0). Infiltration distances were measured from the nearest point on the tumour border. Distances for an image were normalized by dividing by the largest infiltration distance found in that image.

### scRNA-seq of tumour-infiltrating immune cells

C57BL/6J mice were subcutaneously injected with B16 or KPC tumours and treated with AC484, anti-PD-1 or no treatment. A total of 16 (5 untreated, 5 anti-PD-1-treated and 6 AC484-treated) unilateral KPC tumours, and 12 (4 untreated, 4 anti-PD-1-treated and 4 AC484-treated) unilateral B16 tumours from GVAX-vaccinated mice were collected on day 13, weighed and chopped before chemical and mechanical digestion with a tumour dissociation kit (Miltenyi) and a gentleMACS dissociator (Miltenyi) using the m-TDK-1 programme. Isolation of immune cells was performed by density centrifugation with lympholyte reagent (Cedarlane Labs) followed by positive selection for CD45^+^ cells with MicroBeads and a magnetic separator (Miltenyi). Droplet-based isolation of single cells and subsequent preparation of 5′ sequencing libraries were performed using a Chromium Controller with the 10x Genomics platform according to the manufacturer’s specifications. Libraries were prepared utilizing Chromium Next GEM Single Cell Reagent kits 5′ v.1 chemistry or 5′ v.2.2 chemistry. scRNA-seq profiles were generated from 4–6 tumours per treatment condition in each model, resulting in a dataset of 68,060 immune cell transcriptomes from all conditions combined.

### Single-cell data analyses

Pooled equimolar 5′ 10x output libraries were sequenced on an Illumina SP flow cell using a paired-end 150 cycle kit. Downstream preprocessing steps were performed using cellranger (v.5.0.1). Individual replicate quality was evaluated based on the number of cells recovered, mean reads per cell and median genes per cell. All replicates were sequenced at a depth that surpassed Cell Ranger’s recommended mean reads per cell of 25,000. Early quality-control metrics determined that one KPC anti-PD-1-treated tumour had very low cell recovery and was excluded. After this exclusion, 87,134 cells were recovered across conditions. Additional cell and gene filtering was performed using scanpy (v.1.7.2)^57^. Cells with greater than 10% mitochondrial gene content were excluded. In addition, genes that were not recovered in any cell were also excluded from the downstream analysis.

Gene counts were library size normalized to 100,000 and log-transformed with a pseudocount of 1. PCA and nearest neighbour graphs were calculated to visualize on a UMAP projection. Harmony batch correction was then used to correct PCA embeddings for technical batch effects between experiments^58^. Cells were then grouped into 21 distinct clusters using the leiden algorithm. Clusters driven by a high doublet score or markers of low cell viability, such as long noncoding RNA *Malat1*, were excluded. After this additional filtering, 68,080 cells were left for downstream analysis. Cells were re-clustered and classified based on the built-in scanpy function one-vs-rest differentially expression and immune-related gene signatures. To gain more granularity between the T and NK cell subtypes, subclustering was performed on cells in clusters expressing *Cd8a, Cd4* and *Ncr1* transcripts. New PCA embeddings, nearest neighbourhood graphs and harmony batch corrections were calculated for this subgroup on a set of 10,000 highly variable genes. Differentially expressed genes between treatment conditions were calculated using a logistic regression model^59^. Ranked lists of differential genes were created using signed *P* values calculated by the logistic regression model and passed to GSEAPreranked to search for enriched gene sets by treatment^18,55,60^.

### TCR profiling from bulk tumour tissue

B16 cells were subcutaneously injected on the back of 45 C57BL/6J mice. Tumours were treated with anti-PD-1, AC484 or no treatment. On day 12, 100 μg of tissue was collected from each tumour and stored in RNAlater at −80 °C. RNA was extracted using a Tissue Lyser II Homogenizer (Qiagen) and an RNEasy Mini kit (Qiagen) following manufacturer’s instructions. A SMARTer Mouse TCR a/b Profiling kit (Takara) was used to capture complete V(D)J variable regions of TCR transcripts following the manufacturer’s instructions. Complete libraries were sequenced on an Illumina MiSeq using a 600 cycle v.3 chemistry kit.

### TCR sequencing analysis

The package MIXCR^61^ was used to perform quality control and to reconstruct raw reads to quantifiable amino-acid level clonotypes. Replicates were normalized to account for differences in sequencing depth so that each replicate had the same total CloneCount. After read depth normalization, TCRB sequences were clustered into groups of similar sequences, proposed to recognize similar or the same peptide MHCs, in parallel by two similarity metrics: Gliph2 (ref. ^32^) and Levenshtein edit distance^31,62^. Levenshtein edit distance was calculated using python-levenstein 0.12.2 package. A matrix of edit distances was passed to sklearn DBSCAN to call clusters with an epsilon of 1 and minimum samples as 2.

### Library clean-up, quantification and sequencing

Clean-up of all sequencing libraries was performed using Ampure XP (SPRI) beads (Beckman-Coulter). Characterization of all sequencing libraries was performed using BioAnalyzer (Agilent), TapeStation (Agilent) and Qubit (ThermoFisher) instruments. Sample libraries were quantified using a KAPA Library Quantification kit (Roche) when necessary. Pooled equimolar libraries were sequenced on Illumina instruments (MiSeq, NovaSeq and NextSeq 500).

### Immunopeptidomics

#### MHC peptide purification

MHC class I immunopeptidomics was performed on 1 × 10^8^ B16 treated cells. Cells were lysed in 2% CHAPS buffer (120 mM NaCl, 50 mM Tris-Cl pH 8.0 and 2% CHAPS). Cell lysates were ultracentrifuged for 1 h at 50,000*g*. Immunoprecipitation was performed with H-2Kb/H-2Db (clone 28-8-6) crosslinked protein A sepharose beads. MHC-bound beads were extensively washed, and elution was performed with 0.1% trifluoroacetic acid (TFA). Eluted peptides were fractionated and prepared for mass spectrometry (MS) injection.

#### LC–MS/MS method

MHC peptides were separated with an Ultimate 3000 RSLCnano (ThermoFisher Scientific) equipped with an Acclaim PepMap 100 trap column (75 μm i.d. × 20 mm, ThermoFisher Scientific) packed with 3 μm C18 resin, and an Easy Spray ES802 analytical column. Loading solvent buffers were 0.05% (v/v) TFA in water and 0.05% (v/v) TFA in acetonitrile. Analytical column mobile phases were 0.1% (v/v) formic acid (ThermoFisher Scientific) in water (buffer A) and 0.1% (v/v) formic acid in acetonitrile (ThermoFisher Scientific) (buffer B). Peptide data were acquired using a data-dependent acquisition method. Fusion Lumos full scan resolving power was set to 120,000, acquired from 300–1,750 *m/z*. MS1 automated gain control target 400,000 ions, maximum injection time 50 ms, lock mass 445.12 *m/z*. Peptide fragmentation selection criteria were set between 1 × 10^4^ and 1 × 10^12^ intensity range, charge states +1 to +5. Peptides were sampled as follows: +1 charge states if within 750–1,750 *m/z* range, and all +2 to +5 charge states. Fragmented peptides were sampled two times then dynamically excluded from additional selection for 30 s. Peptides were selected with a 1.2 *m/z* quadrupole isolation window. Fragmentation was performed with collision-induced dissociation and higher energy C-trap dissociation (29% and 21% normalized collision energy, respectively). MS/MS peptide fragments were analysed in the Orbitrap with standard AGC targets and dynamic maximum injection times collected in a 5 s cycle time.

#### Data analysis

Raw mass spectrometry files were uploaded into PEAKS software for label-free proteomics analysis. Unique peptide identification was performed at an FDR of 5%, followed by database search (mouse proteome database) to identify peptide source proteins. Total peptides include peptides with exact and partial sequence mapping to the mouse proteome.

### In vitro dendritic cell and macrophage experiments

BMDCs or bone marrow-derived macrophages (BMDMs) were generated from C57BL/6N mouse bone marrow over 14 or 7 days, respectively, by differentiating to BMDCs with 1 ng ml^–1^ GM-CSF (Peprotech) and 100 ng ml^–1^ FLT3L (Peprotech) or to BMDMs with 100 ng ml^–1^ M-CSF (Peprotech). BMDCs and BMDMs were then stimulated for 20 h with 5 or 50 ng ml^–1^ IFNγ (R&D Systems) with or without 5 μg ml^–1^ anti-CD40 antibody (BioLegend, clone 1C10) or rat IgG2A isotype control (BioLegend, clone RTK2758) as indicated in the presence of AC484. Cell-activation markers CD40, CD80, CD86, MHC class I and MHC class II on live CD11b^+^F4/80^+^ BMDMs or CD103^+^ BMDCs were measured by flow cytometry on a Cytek Aurora (Cytek Biosciences). Cytokines and chemokines, including IL12p70, TNF, CXCL10 and CXCL9, in supernatants were measured using a Milliplex Mouse Cytokine/Chemokine Magnetic Bead Panel (MCYTOMAG-70K; Millipore Sigma).

### Tumour-infiltrating T cell isolation and library preparation for RNA-seq and ATAC–seq

B16 cells were subcutaneously injected on the back of 47 C57BL/6J mice. Tumours were treated with anti-PD-1 (*n* = 14), AC484 (*n* = 19) or no treatment (*n* = 14). On day 19 after tumour challenge, tumours were collected, weighed and chopped before chemical and mechanical digestions with a Tumour Dissociation Kit II (Miltenyi) and a gentleMACS Dissociator (Miltenyi) using the m-TDK-1 programme. Isolation of immune cells was performed by density centrifugation with lympholyte reagent (Cedarlane Labs) followed by positive selection for CD8^+^ cells. CD8^+^ T cells were pooled, pre-sorting into samples from 5–11 tumours, depending on the cell count recovered, and then sorted into TIM-3^+^ and SLAMF6^+^ populations with two pooled samples per treatment group. Dead cells were excluded from sorted populations using Fixable Viability Dye eFluor 780 (1:1,000, ThermoFisher).

For ATAC–seq, 15,000 SLAMF6^+^ and 31,000 TIM-3^+^ cells were incubated in 1 µl of transposition reaction mix (containing 2× TD buffer, Tn5 transposase and 1% digitonin) per 1,000 cells for 30 min at 37 °C with agitation at 1,000 r.p.m. DNA was purified using a Qiagen MinElute Reaction kit. Samples were sequenced on an Illumina NovaSeq sequencer using paired-end 50 bp reads.

For RNA-seq, 1,000 SLAMF6^+^ and 10,000 TIM-3^+^ cells were lysed and RNA was isolated from cell pellets using a Qiagen RNeasy Micro kit according to the manufacturer’s instructions. First-strand Illumina-barcoded libraries were generated using a NEBNext Ultra II Directional RNA Library Prep kit according to the manufacturer’s instructions, a NEBNext Poly(A) mRNA Magnetic Isolation Module and a 16-cycle PCR enrichment step. Libraries were sequenced on an Illumina NextSeq 500 instrument using single-read 37-bp reads.

### Tumour-infiltrating T cell RNA-seq and ATAC–seq data pre-processing

Raw sequencing reads were demultiplexed using bcl2fastq (v.2.20.0). Adapter sequences were trimmed using Trimmomatic (v.0.36). Pre-trimming and post-trimming quality control was done using FastQC (v.0.11.7).

### Tumour-infiltrating T cell RNA-seq analysis

Trimmed reads were quantified by pseudoalignment to mm10 using Kallisto (v.0.46.0). RNA-seq transcript counts were quantified using the tximport (v.1.24.0) R package. A vst step was applied to the counts matrix, and differentially expressed genes were quantified using the DESeq2 (v.1.36.0) R package. PCA was done using DESeq2. GSEA was performed using the GSEAPreranked^18,55^. For GSEA, a ranked list of genes was formed on the basis of the signed adjusted *P* value from DESeq2 differential expression. Genes that had a DEseq2 adjusted *P*  value of NA were not considered. Unbiased GSEA was performed on MSigDB Mouse Hallmark gene sets (v.2023.1.Mm). From the Human MSigDB v.2023.1.Hs collection, the memory signature was defined as the overlapping genes between GSE9650_EFFECTOR_VS_MEMORY_CD8_TCELL_DN and GSE9650_EXHAUSTED_VS_MEMORY_CD8_TCELL_DN. The effector gene signature was defined as the overlap between GSE9650_EFFECTOR_VS_EXHAUSTED_CD8_TCELL_UP and GSE9650_EFFECTOR_VS_MEMORY_CD8_TCELL_UP. The exhausted signature was defined as the overlap between GSE9650_EFFECTOR_VS_EXHAUSTED_CD8_TCELL_DN and GSE9650_EXHAUSTED_VS_MEMORY_CD8_TCELL_UP. Human genes from these signatures were mapped to mouse genes using http://www.informatics.jax.org/downloads/reports/HOM_MouseHumanSequence.rpt. IL-2 + anti-PD-L1 and anti-PD-L1 gene sets were derived from a previously published RNA-seq counts matrix (GSE206739). Differential expression between the IL-2+anti-PD-L1 and anti-PD-L1 conditions was done using DESeq2. Gene signatures were defined by those genes with magnitude of log fold change greater than 2.5 and *P* adjusted value of less than 0.05.

### Tumour-infiltrating T cell ATAC–seq analysis

Trimmed reads were aligned to mm10 using Bowtie2 (v.2.5.0). Outputs (.sam) from Bowtie2 were converted to bam files and sorted using samtools (v.1.6). PCR duplicates were marked using picard MarkDuplicates (v.2.27.5). Peaks were called using macs2 (v.2.2.7.1) on combined bam files for each sample condition with a *q*-value threshold of 0.01. Outputs (.narrowPeak) from each condition were merged to form a single peak universe. Using bedtools (v.2.30.0), the single peak universe was sorted, overlapping peaks were merged, and peaks within blacklisted regions of the mm10 genome were removed. For each biological replicate, the number of reads intersecting each of the single universe peaks was quantified to form a counts matrix. A variance stabilizing transformation (vst) was applied to the counts matrix, and differential expression was performed using DESeq2 (v.1.36.0). Differentially accessible peaks were defined by having an adjusted p-value less than 0.05. Principal component analysis was performed using DESeq2 on the 5,000 peaks with highest variance among samples. Averaged vst normalized counts for conditions were K-means clustered using Morpheus (https://software.broadinstitute.org/morpheus/). Optimal number of *K*-means clusters was found using the Elbow Method (yellowbrick v1.3). Using GREAT (v.4.0.4, http://great.stanford.edu/great/public/html/) and default settings, peaks were mapped to nearby genes. Average vst normalized RNA-seq counts for the mapped genes from each ATAC–seq defined cluster were visualized using Morpheus. ATAC–seq tracks were visualized using Integrative Genomics Viewer (v.2.15.4, Broad Institute) on tdf files using the Normalize Coverage Data feature. Enrichment of gene sets was found using the following procedure: for each comparison, differential peaksets in condition A and condition B were defined by those with an adjusted *P* value less than 0.05. Condition A and condition B peaksets were mapped to genesets. Genes which existed in both the condition A and condition B genesets were removed. A hypergeometric test (scipy v.1.7.3) was used to determine whether genesets of interest were differentially enriched in the condition A geneset vs condition B geneset. FDR Correction for multiple hypothesis testing was performed using the Benjamini–Hochberg procedure (statsmodels v.0.13.5). GSEA was performed on MSigDB Mouse Hallmark Genesets (v.2023.1.Mm). IL-2+anti-PD-L1 and anti-PD-L1 genesets were derived from a previously published ATAC–seq counts matrix (Gene Expression Omnibus database identifier GSE206739). Differentially accessible peaks were identified using DEseq2. The IL-2 + anti-PD-L1 and anti-PD-L1 peak sets contained peaks with a magnitude of log(fold change) greater than 1 and adjusted *P* value less than 0.05. Peak sets were mapped to genes using GREAT. Genes that existed in both gene sets following this method were removed. Motif enrichment analysis was performed using HOMER (v.4.11.1).

### Flow cytometry analysis of TILs

Mice were subcutaneously injected with 6 × 10^4^ B16 cells and treated with AC484, anti-PD-1 or no treatment, as above. Tumours were collected on day 14 or day 20 for pSTAT5 staining, weighed and chopped before chemical and mechanical digestion with a Tumour Dissociation kit (Miltenyi) and a gentleMACS Dissociator (Miltenyi) using the m-TDK-1 programme. The resulting cell suspension was passed through a 70-µm filter. Isolation of immune cells was performed by density centrifugation with lympholyte reagent (Cedarlane Labs) followed by positive selection for CD45^+^ cells with MicroBeads and a magnetic separator (Miltenyi). Cells were blocked with TruStain FcX Plus (anti-mouse CD16/32) antibody (1:100, BioLegend) in PBS with 2% FBS. Dead cells were excluded using Live/Dead Fixable Blue Dead Cell stain (1:1,000, ThermoFisher) added concurrently with surface antibodies. Samples were stained with indicated antibodies for 30 min on ice. After washing, cells were fixed using a FOXP3/Transcription Factor Staining buffer set (eBiosciences) as per the manufacturer’s instructions, blocked with TruStain FcX, mouse and rat serum, then stained with intracellular antibodies. For pSTAT5 staining, cells were fixed in 4% paraformaldehyde for 15 min at 37 C and then permeabilized with 90% methanol for 20 min on ice before proceeding with blocking and staining for intracellular and extracellular markers. Spherotech AccuCount Fluorescent particles were added for cell quantification before analysis on a Beckman Coulter Cytoflex LX flow cytometry system using single-colour compensation controls and fluorescence-minus-one thresholds to set gate margins. One-way analysis of variance was used to make comparisons between groups. Generated *P* values were adjusted for multiple comparisons.

### Immunophenotyping

On day 8 of dosing, animals were euthanized, and tumours, spleens and blood were collected for flow cytometry analysis. Tumours were dissociated on a gentleMACS following the protocol of the Tumour Dissociation kit (Miltenyi BioTec). Single-cell suspensions from all three tissues were prepared, stained with the immunophenotyping antibody panel (Supplementary Table 10) and acquired on a Cytek Aurora. Flow cytometry data files were excluded when total cellularity, that is gated live, single cells, was fewer than 1,000 events. Furthermore, calculated subsets were omitted when the number of events in key lineages dropped below 500 events. This criterion was applied to the total T cell gate in the T cell panel, and the macrophage gate in the myeloid panel. The gating strategy for immunophenotyping is shown in Extended Data Fig. 6.

### Western blotting

Whole cell lysates from either primary mouse T cells or tumour cells were prepared using RIPA lysis and extraction buffer (ThermoFisher Scientific), Halt protease inhibitor cocktail (ThermoFisher Scientific) and PhosSTOP (Roche). Sample protein concentration was measured using a Pierce BCA Protein Assay kit (ThermoFisher Scientific) before addition of NuPAGE LDS sample buffer (ThermoFisher Scientific) and dithiothreitol. Subsequently, samples were heated to 70 °C for 10 min and 20–30 μg of protein was loaded on NuPAGE 4–12% Bis-Tris gels (ThermoFisher Scientific) in NuPAGE MOPS SDS running buffer (ThermoFisher Scientific). For assays with limited number of cells (<1 × 10^5^), cell pellets were lysed in 2× Laemmli buffer (Millipore Sigma) with Halt protease inhibitor cocktail (ThermoFisher Scientific) and PhosSTOP (Roche). Samples are heated to 95 °C for 5 min before proceeding with gel loading. Protein was transferred to nitrocellulose membranes using iBlot 2 (ThermoFisher Scientific) and washed with Tris-buffered saline plus 0.1% Tween 20 (TBS-T) before blocking for 1 h at room temperature with Intercept (TBS) blocking buffer (LI-COR). The primary antibody was then added and incubated overnight at 4 °C before again washing with TBS-T. Fluorescent secondary antibodies were added in (TBS) blocking buffer and incubated for 1 h at room temperature and again washed with TBS-T. Blots were then visualized using a LI-COR Odyssey CLx and analysed with ImageStudio Lite and ImageJ software.

### Stimulation protocols for TCR signalling analyses

For signalling assays, CD8^+^ T cells were isolated from spleens of 7-to-12-week-old female C57BL/6J mice using a CD8a^+^ T Cell Isolation kit (Miltenyi Biotec). T cells (2 × 10^6^ ml^−1^) were incubated in T cell medium with anti-CD3 (2.5 µg ml^−1^) crosslinked with anti-hamster IgG1 and with or without AC484 (20 µM) for 5–15 min at 37 °C. After stimulation, cells were washed with PBS and lysed in modified RIPA buffer as described above.

To assess STAT5 phosphorylation, CD8^+^ splenocytes were similarly isolated and incubated in T cell medium with plate-bound anti-CD3 (5 µg ml^−1^), soluble anti-CD28 (2 µg ml^−1^), and stimulated with IL-2 (100 ng ml^−1^) for 48 h. Cells were then expanded in fresh plates with IL-2 (100 ng ml^−1^) in T cell medium for 72 h and stimulated for 1 or 20 h with no treatment, AC484 (20 µM) or IL-2 (100 ng ml^−1^), or both. After stimulation, cells were washed with PBS and analysed by western blotting or flow cytometry.

### In vitro cytokine stimulation and blocking

Pan T cells from the spleens of 7-week-old WT female C57BL/6J mice were extracted using a Pan T Cell Isolation kit II (Miltenyi Biotec) and plated in T25 flasks in RPMI with 10% FBS and 10 mM HEPES buffer, 55 µM 2-mercaptoethanol, 1× MEM non-essential amino acids (NEAA) solution (ThermoFisher Scientific) and 1 mM sodium pyruvate. The following cytokines were serially added to each treatment group and incubated for 1 h at 37 °C before centrifugation and cell lysis: IL-2 (100 ng ml^−1^, BioLegend), IFNγ (1 ng ml^−1^, Peprotech), anti-IL-2 monoclonal antibody (2 μg ml^−1^, Invitrogen) and anti-mouse CD132 (common γc inhibitor) (36 μg ml^−1^, BioXCell). For treatments containing both IL-2 and neutralizing antibodies (anti-IL-2 and anti-CD132), solutions containing the cytokine and neutralizing antibodies were combined and incubated for 2 min at room temperature before addition to the flask.

### Metabolic analysis

Metabolic analysis of T cells was carried out using a Seahorse Extracellular Flux Analyzer XF^e^96 (Seahorse Bioscience). Mitochondrial respiration was measured by oxygen consumption using a XF Cell Mito Stress Test kit (Seahorse Bioscience). Pan T cells were isolated from spleens of 7-week-old female C57BL/6J mice using a Pan T Cell Isolation kit II (Miltenyi Biotec). T cells were incubated with plate-bound anti-CD3 (1 µg ml^−1^) and soluble anti-CD28 (2 µg ml^−1^), and stimulated with IL-2 (100 ng ml^−1^) and IFNγ (1 ng ml^−1^) with or without AC484 (20 µM) for 3 days. T cells were seeded at a density of 495,000 per well in a poly-l-lysine-coated Seahorse XF96 Cell Culture Microplate in RPMI 1640 base medium (US Biological, R9011, pH 7.4) supplemented with 10 mM glucose, 2 mM glutamine and 1 mM sodium-pyruvate (all Sigma-Aldrich). Assays were performed according to the manufacturer’s instructions using 1.5 µM oligomycin to inhibit ATP synthase, 1 µM FCCP to uncouple oxygen consumption from ATP production and 0.5 µM rotenone and antimycin A to stop the electron transport chain. Data were analysed using Wave Software (Seahorse Bioscience). Using Prism, the data were statistically assessed using a multiple unpaired *t*-test or a two-way analysis of variance.

### EL4-OVA adoptive cell therapy model

C57BL/6J mice were subcutaneously implanted with 1 × 10^6^ EL4-OVA (ATCC, no Matrigel) on day 0. On day 7, naive WT OT-I CD8^+^ whole splenocytes were stimulated with 100 ng ml^–1^ OVA I (Anaspec, AS-60193) with or without AC484 (100 nM) for 48 h then expanded with fresh medium, drug and exogenous IL-2 (10 ng ml^−1^) (Peprotech, 212-12) for an additional 2 days. Viable day 4 effector CD8^+^ T cells were enriched through Ficoll gradient for adoptive transfer. On day 11, EL4-OVA tumour-bearing mice received 7.5 × 10^5^ vehicle or AC484-treated OT-I CD8^+^ T cells. Mice were randomized into size-matched groups on the day of transfer, and tumour growth was monitored over time.

### Primary mouse NK activation and tumour cell killing assays

Primary mouse NK cells were negatively selected using an EasySep Mouse NK isolation kit (StemCell Technologies) from spleens of C57BL/6N mice and cultured in T/NK cell medium containing 10 ng ml^–1^ IL-2 (R&D Systems) and 10 ng ml^–1^ IL-15 (BioLegend). Medium was changed every 2 days. After 8 days, NK cells were cultured in T/NK cell medium containing 10 ng ml^–1^ IL-2, 10 ng ml^–1^ IL-15 and 100 ng ml^–1^ IL-18 (MBL International) and treated with AC484 for 1 h, with an additional 4 h in the presence of GolgiStop (BD Biosciences). Cells were stained with Zombie Green (BioLegend) for dead-cell exclusion followed by surface staining with anti-NK1.1 antibody. Cells were fixed and permeabilized using a Cytofix/Cytoperm Fixation/Permeabilization kit (BD Biosciences), intracellularly stained for IFNγ and GZMB, and analysed on an LSR Fortessa X-20 (BD Biosciences).

For tumour cell killing assays, NK cells were stained with Cell Trace Violet (ThermoFisher Scientific) and YAC-1 tumour cells were stained with Cell Trace Far Red (ThermoFisher Scientific). NK and YAC-1 cells were cultured at a 1:4 E:T ratio in medium containing 10 ng ml^–1^ IL-2, 10 ng ml^–1^ IL-15 and 100 ng ml^–1^ IL-18 with increasing concentrations of AC484. After 24 h, cells were stained for viability with Zombie Green. Samples were acquired on a Quanteon flow cytometer (NovoCyte), and the frequency of killed YAC-1 cells (Zombie Green positive cells) of total YAC-1 cells (Cell Trace Far Red positive cells) was quantified.

### Mouse in vitro repeat antigen stimulation

Bulk CD8 T cells were isolated from splenocytes of 4–6-week-old female OT1 mice (C57BL/6-Tg(TcraTcrb)1100Mjb/J: The Jackson Laboratory, 003831) using a total CD8 isolation kit (StemCell Technology, 19853). CD8 T cells were plated at 1 million per ml in complete T cell medium, consisting of RPMI with 10% FBS and 10 mM HEPES buffer, 55 µM 2-mercaptoethanol, 1× MEM NEAA solution (ThermoFisher Scientific) and 1 mM sodium pyruvate, with the addition of 5 ng ml^–1^ each of recombinant mouse IL-7 (Peprotech, 217-17-10UG) and recombinant mouse IL-15 (Peprotech, 210-15-10UG). Cells were cultured according to a previously published protocol^63^. In brief, for single peptide stimulation, cells were cultured in the presence of 10 ng ml^–1^ of OVA(257–264) peptide (Invivogen, vac-sin) for the first 48 h in complete T cell medium. The peptide was then removed by washing the cells with complete medium, and for the remaining 3 days, the cells were cultured in complete T cell medium. For repeat peptide stimulation, fresh 10 ng ml^–1^ OVA(257–264) peptide was added daily for a total of five stimulations in complete T cell medium. Each day, the peptide was removed by washing the cells in complete medium, the cells were counted and replated at 1 million per ml with fresh OVA peptide. AC484 (1 µM or 20 µM) or DMSO control was added after the second stimulation and was added fresh every day with the OVA peptide for the last three stimulations. Unstimulated control cells were cultured in complete T cell medium.

To measure intracellular cytokine production, on the day after the fifth stimulation, cells were treated with GolgiPlug (BD Biosciences, 555029) in fresh complete T cell medium for 6 h before staining cells for FACS analysis. Cells were stained for live/dead and extracellular markers first and then permeabilized using a Fixation/Permeabilization kit (FOXP3/Transcription Factor Staining buffer set, eBioscience, 00-5523-00) followed by intracellular staining (Supplementary Table 1) overnight. All data were acquired the next day on Cytek Aurora and analysed using FlowJo software (FlowJo_v10.7.1).

### Mouse in vitro killing assay: 3D spheroid co-culture

The day before the assay, B16-OVA Nuclight Red cells were detached with accutase and resuspended in T cell basal medium (complete T cell medium without cytokines). Next 2,000 tumour cells were plated per well of a 96-well ultralow adhesion plate (Corning, 7007) in 100 µl volume. Plates were centrifuged at 1000 r.p.m. for 5 min and incubated overnight. The assay began on the day after the fifth stimulation of the above in vitro exhaustion protocol. OT1 T cells were washed and resuspended in T cell basal medium, and 6,000 viable cells per well were added in 100 µl for a final volume of 200 µl per well. A total of 6 wells were used per condition. Plates were placed in an Incucyte SX5 (Sartorius) and imaged every 1 or 2 h for up to 2.5 days. Total fluorescent object integrated intensity/area image was collected and analysed using the Incucyte Spheroid Analysis software module (Sartorius).

### Human in vitro T cell repetitive stimulation assay

Primary healthy human donor T cells were isolated from a fresh Leukopak (StemCell Technologies) using a EasySep Human T cell isolation kit (StemCell Technologies, 17951) and frozen. After thawing, T cells were cultured in RPMI containing 10% FBS, 1× NEAA, 10 mM HEPES, 1 mM sodium pyruvate, 55 µM 2-mercaptoethanol and 100 IU ml^–1^ IL-2 (StemCell Technologies, 78036) and allowed to rest for 4 h before co-culture.

For the cytotoxicity assay, A375 melanoma cells expressing luciferase and membrane-tethered anti-CD3 scFv and human CD80 (adapted from ref. ^43^) were plated in 6-well plates and allowed to adhere for 4–6 h. Subsequently, human T cells were added at a 1:2 E:T ratio in medium containing 0, 0.1 or 1 µM A484. T cells were maintained in A484 throughout the experiment. After 3 days of culture, T cells were collected from the plate and counted to measure expansion and replated at a 1:2 E:T ratio on fresh A375 target cells. T cell killing capacity was measured at baseline and after each re-stimulation by co-culturing T cells and Firefly luciferase expressing A375 target cells in 96-well plates at various E:T ratios with or without A484 treatment. After 72 h incubation, luciferase activity was measured using a Promega Luciferase Bright Glo assay. Relative killing was calculated by comparing the luciferase intensity of no target control wells (100% killed) and no effector control wells (0% killed).

### T cell mitochondrial content

Pan T cells were isolated from spleens of 7-week-old female C57BL/6J mice using a Pan T Cell Isolation kit II (Miltenyi Biotec). Mitochondrial content was measured using MitoTracker Deep Red FM (5 nM) after T cells (2 × 10^5^) were activated with anti-CD3 (1 µg ml^−1^), anti-CD28 (2 µg ml^−1^), incubated with IL-2 (100 ng ml^−1^) and treated with or without AC484 for 96 h in 96-well round-bottom plates in complete T cell medium at 37 °C.

### Antibodies

The following primary antibodies were used to detect designated protein expression in western blot assays: anti-TCPTP (Abcam, ab180764); STAT1 (Cell Signaling, 9172); phospho-STAT1 (Cell Signaling, 9167); STAT5 (Cell Signaling, 94205); phospho-STAT5 (Cell Signaling, 9359); LCK (Invitrogen, AHO0472); phospho-LCK (Cell Signaling, 70926); phospho-SRC family kinase (Cell Signaling, 6943); and FYN (Cell Signaling, 4023). For flow cytometry, the following anti-mouse fluorochrome-conjugated antibodies were used: CD8α (clone 53-6.7, BioLegend or BD Biosciences); CD4 (clone RM4-5 or GK1.5, BioLegend); TCRβ (clone H57-597, BioLegend); Tim-3 (clone 5D12, BD Biosciences or clone RMT3-23, BioLegend); Lag-3 (clone C9B7W, BD Biosciences); CD45 (clone 30-F11, ThermoFisher); CD45.2 (clone 104, BioLegend); NK1.1 (clone 108741, BioLegend); CD44 (clone 103028 or IM7, BioLegend); FOXP3 (clone JFK-16s, eBioscience); Granzyme B (clone GB11, BioLegend); Tox (clone TXRX10, eBioscience); Perforin (clone S16009B, BioLegend); PD-L1 (clone 10 F.9G2, BioLegend); MHC-I (clone 28-8-6, BioLegend); MitoTracker Deep Red FM (ThermoFisher Scientific); CD278 (clone C3978.4A, BD Biosciences); CD27 (clone LG.3A10, BD Biosciences); KLRG1 (clone 2F1, BD Biosciences); CD69 (clone H1.2F3, BD Biosciences); Slamf6 (clone 13G3, BD Biosciences), PD-1 (clone 29 F.1A12, BioLegend); Ki-67 (clone B56, BD Biosciences); CD62L (clone MEL-14, BioLegend); CTLA-4 (clone UC10-4F10-11, BD Biosciences); TCF7 (clone 2203, Cell Signaling); TNF (clone MP6-XT22, BioLegend); CD3 (clone 17A2, BioLegend); IFNγ (clone XMG1.2, BioLegend); STAT5 (Cell Signaling, 94205); phospho-STAT5 (Cell Signaling, 9359); and anti-rabbit IgG (H+L), F(ab′)2 fragment (Cell Signaling, 4412).

### Statistics

Statistical analyses were conducted, and *P* values determined using GraphPad Prism software. T two-tailed unpaired Student’s *t*-test was used for analysis between groups. Compound dose–response curves were determined using a four-parameter logistic-nonlinear regression model from which IC_50_ or half-maximal effective concentration (EC_50_) values were calculated. Statistical analysis and *P* values for mouse efficacy studies were determined using Welch’s *t*-test of tumour volume between noted groups or per cent tumour growth inhibition (TGI) and log-rank (Mantel–Cox) test (per cent survival). TGI was calculated as follows: 1 – (mean tumour volume of treatment group/mean tumour volume of treatment control group) × 100. TGI was based on data collected at the same study time point. Statistical analysis and *P* values are derived from Student *t*-test comparison (one-sided two-sample) using the log-transformed ratio of the tumour volume to the baseline. Measurements were taken from distinct samples. Error bars represent standard error of the mean. Measurements were taken from distinct samples. Error bars represent the standard error of the mean. For human data, owing to patient-to-patient variability in baseline values, data were normalized defining the highest and lowest values as 100% and 0%, respectively. Representative dose–response curves using raw data values from one healthy donor are shown in Extended Data Fig. 1. *P < 0.05, **P < 0.01, ***P < 0.001, ****P < 0.0001. For all mouse experiments, no statistical methods were used to predetermine sample sizes, but at least five mice were included in each group, based on previous publications^3^. Data distribution was assumed to be normal, but this was not formally tested. Animals were randomized before treatment. Data collection and analysis were not performed blinded to the conditions of the experiment. No data points were excluded from analysis.

### Reporting summary

Further information on research design is available in the Nature Portfolio Reporting Summary linked to this article.

## Online content

Any methods, additional references, Nature Portfolio reporting summaries, source data, extended data, supplementary information, acknowledgements, peer review information; details of author contributions and competing interests; and statements of data and code availability are available at 10.1038/s41586-023-06575-7.

### Supplementary information

Supplementary Fig. 1 a, Gating strategies for Fig. 6. b, Gating strategies for Extended Data Fig. 8. c, Full western blot scans with MW markers for Fig. 6f and Extended Data Figs. 1e,o,p and 8g,h.

Reporting Summary

Supplementary Table 1 AC484 kinase selectivity screen.

Supplementary Table 2 AC484 receptor selectivity screen.

Supplementary Table 3 GSEA results for AC484-treated and PTPN2/N1 dual deletion cells vs control untreated cells.

Supplementary Table 4 Nanostring differential expression of AC484-treated human PBMCs isolated from whole blood.

Supplementary Table 5 scRNA-seq analysis of CD45^+^ cells from B16 tumours and KPC tumours.

Supplementary Table 6 Pseudobulk differential expression of key anti-inflammatory, proinflammatory and antigen-processing genes from scRNA-seq of CD45^+^ TILs from B16 tumours and KPC tumours.

Supplementary Table 7 scRNA-seq analysis on re-clustered lymphoid cells isolated from B16 tumours and KPC tumours.

Supplementary Table 8 Differentially accessible peaks in ATAC–seq of tumour-infiltrating CD8^+^ T cells.

Supplementary Table 9 Differentially expressed genes in RNA-seq of tumour-infiltrating CD8^+^ T cells.

Supplementary Table 10 Antibodies used for flow cytometry.

### Source data

Source Data Fig. 1

Source Data Fig. 2

Source Data Fig. 3

Source Data Fig. 4

Source Data Fig. 5

Source Data Fig. 6

Source Data Extended Data Fig. 1

Source Data Extended Data Fig. 2

Source Data Extended Data Fig. 3

Source Data Extended Data Fig. 4

Source Data Extended Data Fig. 5

Source Data Extended Data Fig. 6

Source Data Extended Data Fig. 7

Source Data Extended Data Fig. 8

Source Data Extended Data Fig. 9

## Figures and Tables

**Extended Data Table 1:**
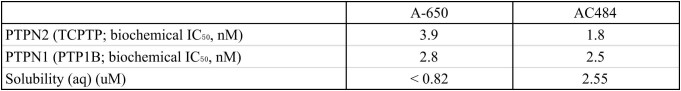
PTPN2/N1 inhibitor biochemical inhibition and solubility

**Extended Data Table 2:**
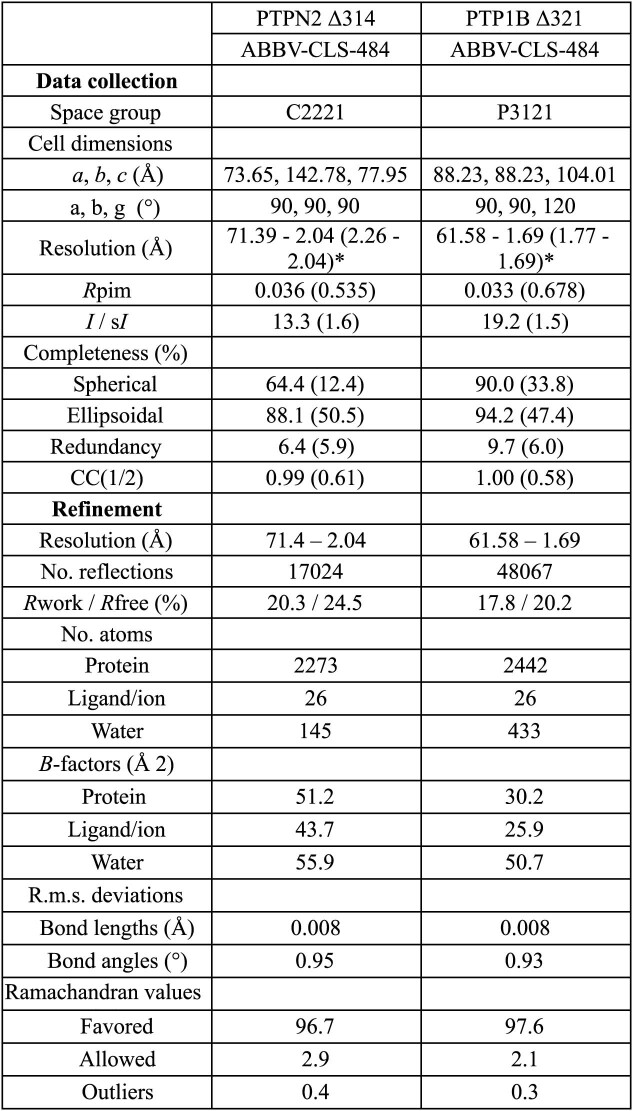
Critical interactions with PTPN2 and PTPN1 active sites

**Extended Data Table 3:**
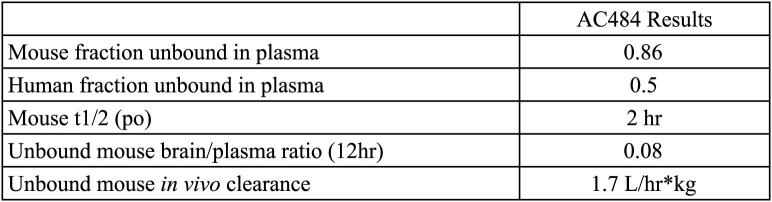
Unbound AC484 levels in plasma and brain

**Extended Data Table 4:**
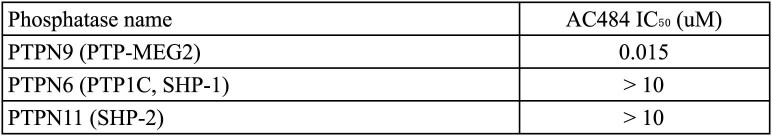
Selectivity profile of AC484 against different phosphatases

**Extended Data Table 5:**
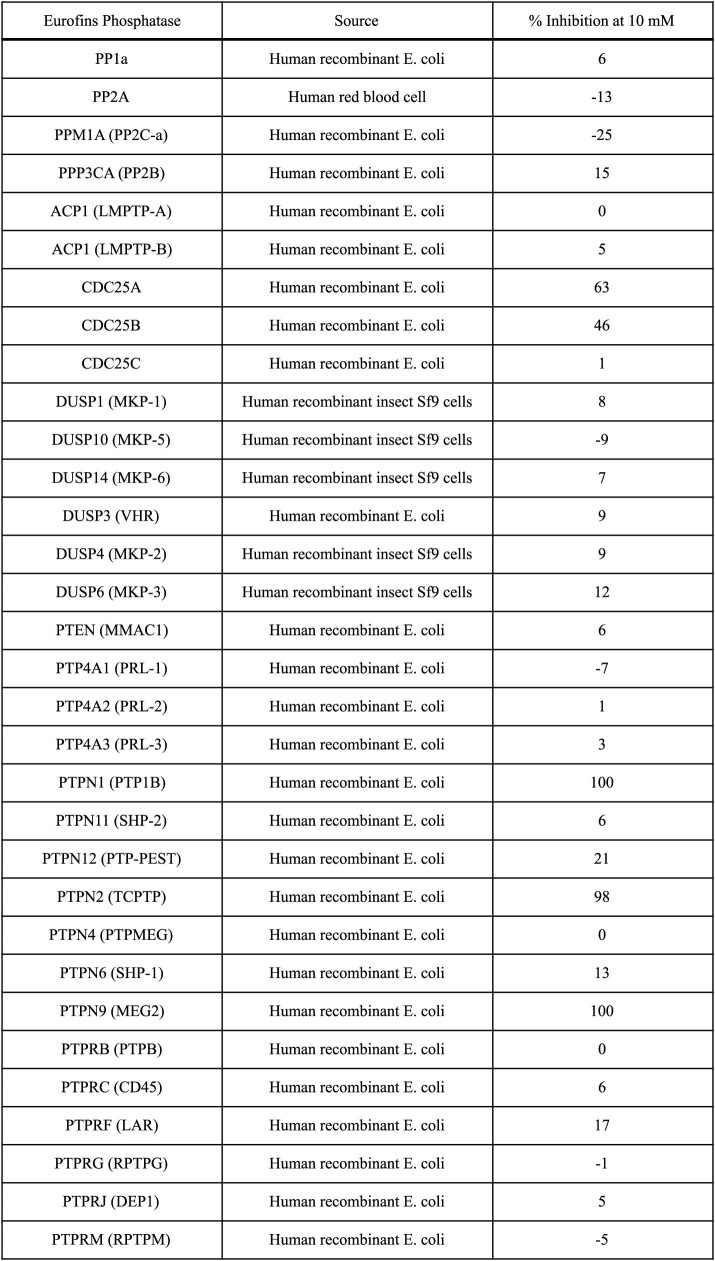
AC484 phosphatase selectivity screen

**Extended Data Table 6:**
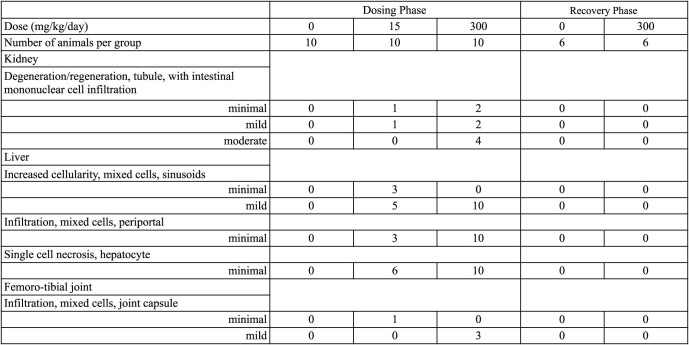
AC484 rat toxicity study

## Data Availability

Atomic coordinates and X-ray diffraction data were deposited in the Protein Data Bank with the accession number 7UAD. Raw data for scRNA-seq, bulk transcriptomic and ATAC–seq studies are available through the Gene Expression Omnibus database with accession number GSE237378. All other data used in the manuscript can be made available by the authors upon request. Source data are provided with this paper.
