# Supplementary material for: The PTPN2/PTPN1 inhibitor ABBV-CLS-484 unleashes potent anti-tumour immunity
Source: Nature. 2023 Oct 4;622(7984):850–62. doi: 10.1038/s41586-023-06575-7 (PMC10599993; doi:10.1038/s41586-023-06575-7)
Supplement: Supplementary file 1 — a, Gating strategies for Fig. 6. b, Gating strategies for Extended Data Fig. 8. c, Full western blot scans with MW markers for Fig. 6f and Extended Data Figs. 1e,o,p and 8g,h. [file 41586_2023_6575_MOESM1_ESM.pdf]

nature portfolio
https://doi.org/10.1038/s41586-023-06575-7

Supplementary information

The PTPN2/PTPN1 inhibitor ABBV-CLS-484 unleashes potent anti-tumour immunity

In the format provided by the authors and unedited

a

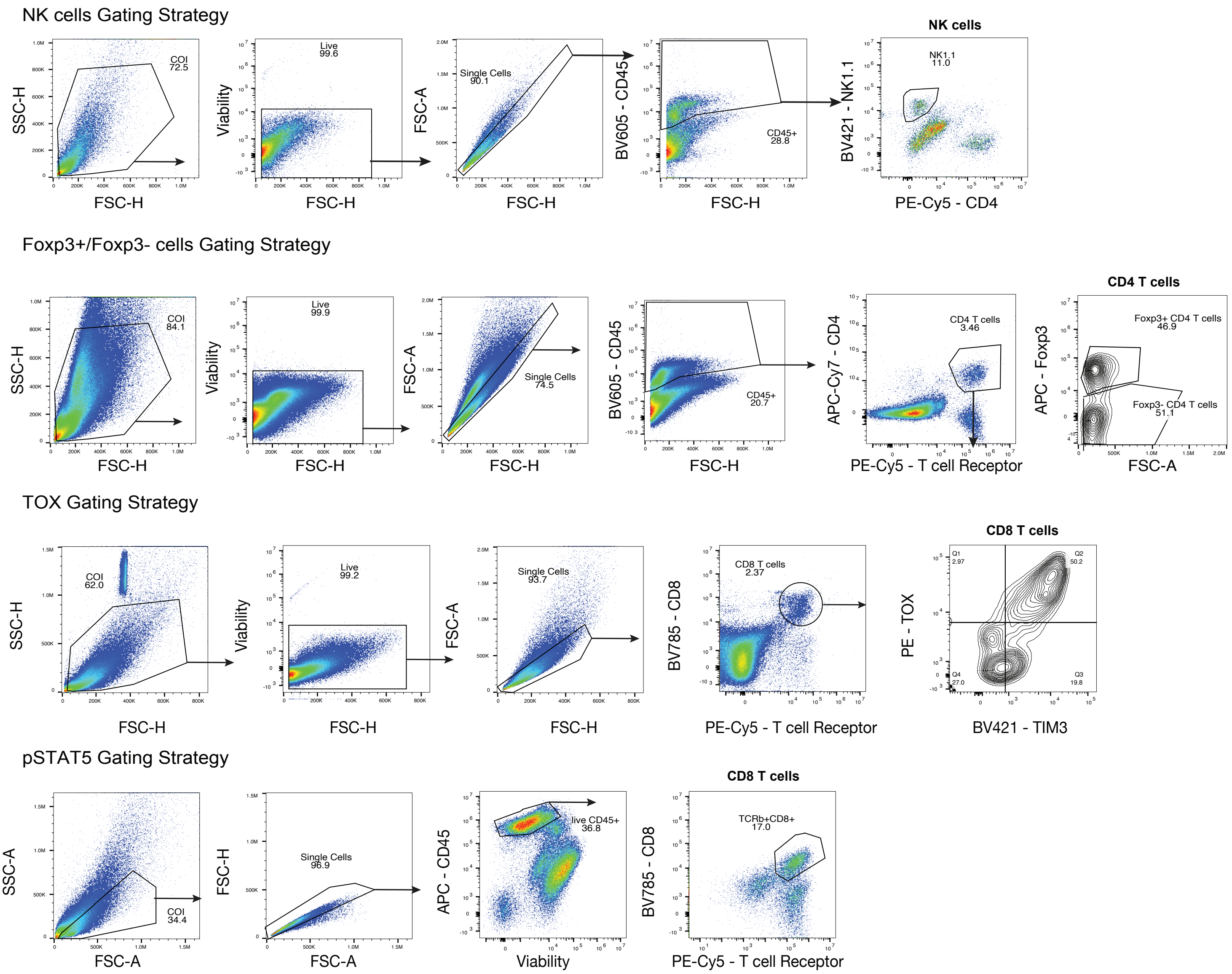

NK cells Gating Strategy
COI 72.5
Live 99.6
Single Cells 90.1
CD45+ 28.8
NK cells
NK1.1 11.0
SSC-H
FSC-H
Viability
FSC-A
BV605 - CD45
BV421 - NK1.1
PE-Cy5 - CD4
Foxp3+/Foxp3- cells Gating Strategy
COI 84.1
Live 99.9
Single Cells 74.5
CD45+ 20.7
CD4 T cells 3.46
CD4 T cells
Foxp3+ CD4 T cells 46.9
Foxp3- CD4 T cells 51.1
APC-Cy7 - CD4
PE-Cy5 - T cell Receptor
APC - Foxp3
TOX Gating Strategy
COI 62.0
Live 99.2
Single Cells 93.7
CD8 T cells 2.37
CD8 T cells
Q1 2.97
Q2 50.2
Q3 19.8
Q4 27.0
BV785 - CD8
PE - TOX
BV421 - TIM3
pSTAT5 Gating Strategy
COI 34.4
Single Cells 96.9
live CD45+ 36.8
TCRb+CD8+ 17.0
SSC-A
APC - CD45

b

CT26 TILs Gating Strategy

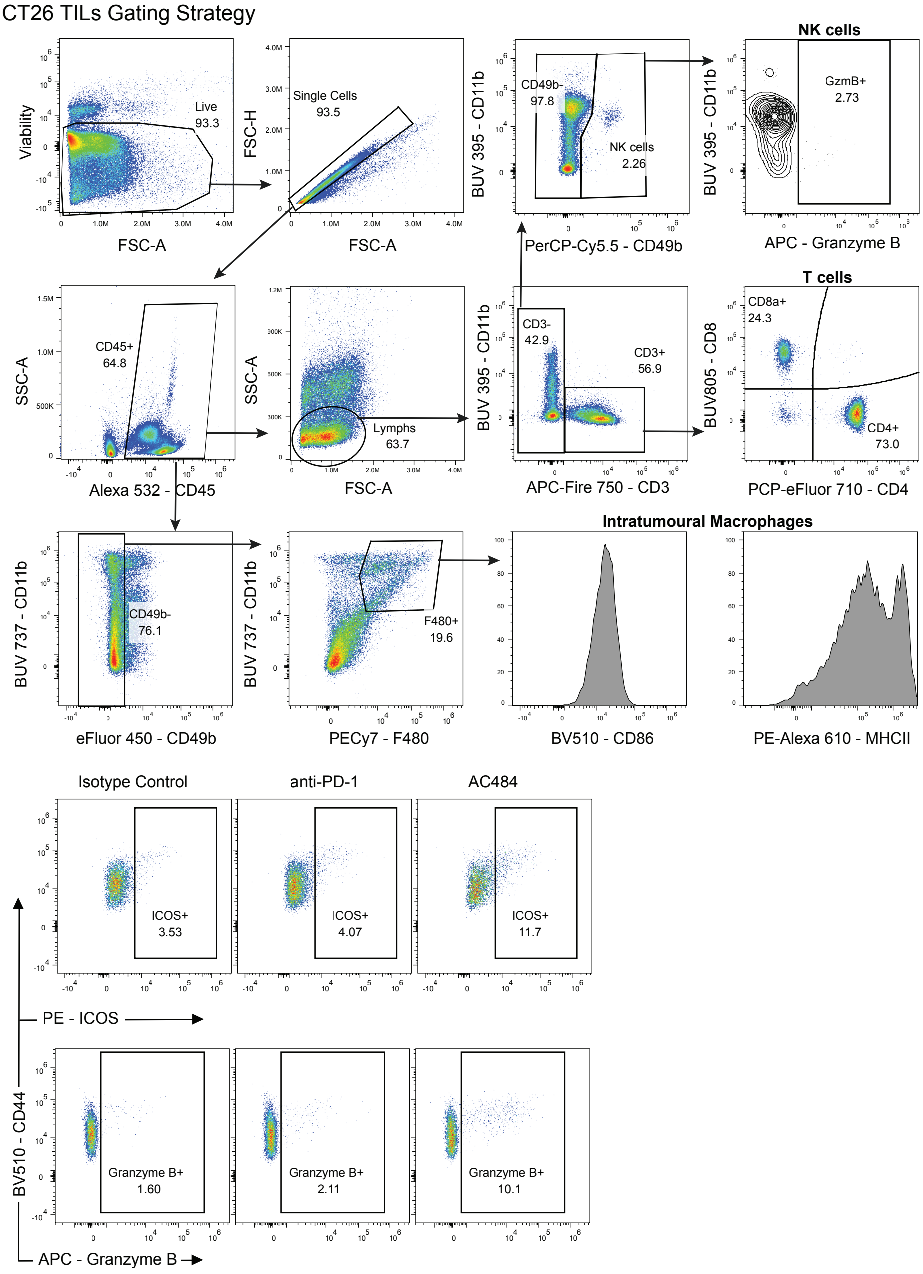

c

Figure 6f

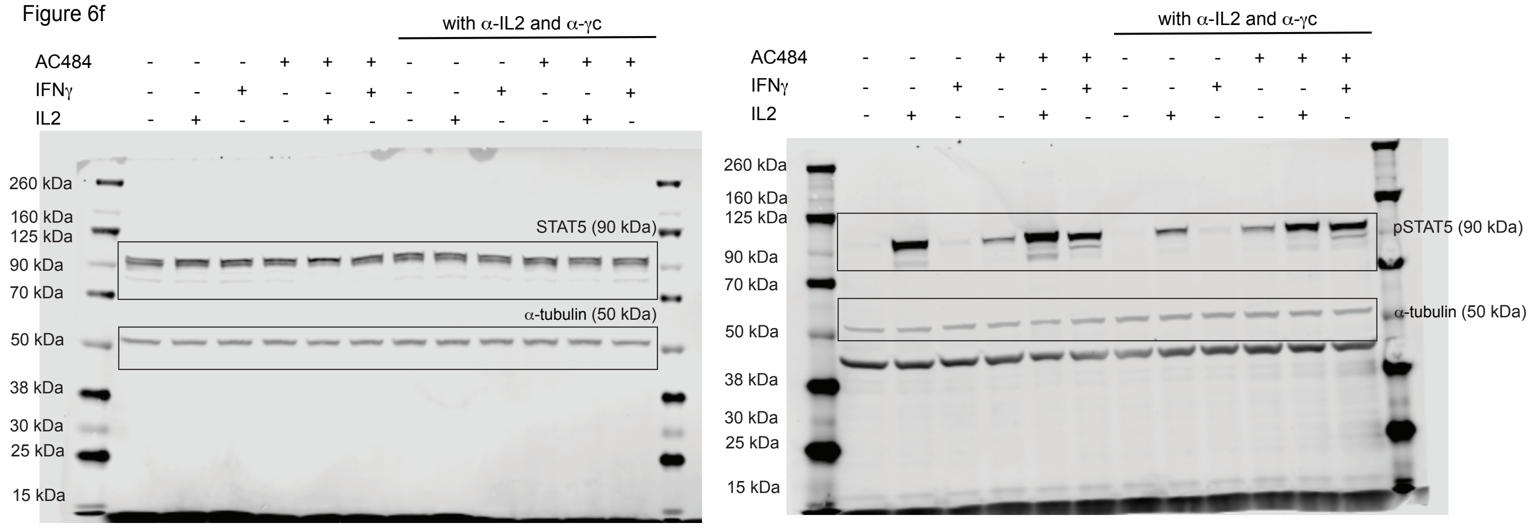

Extended Data Figure 1e

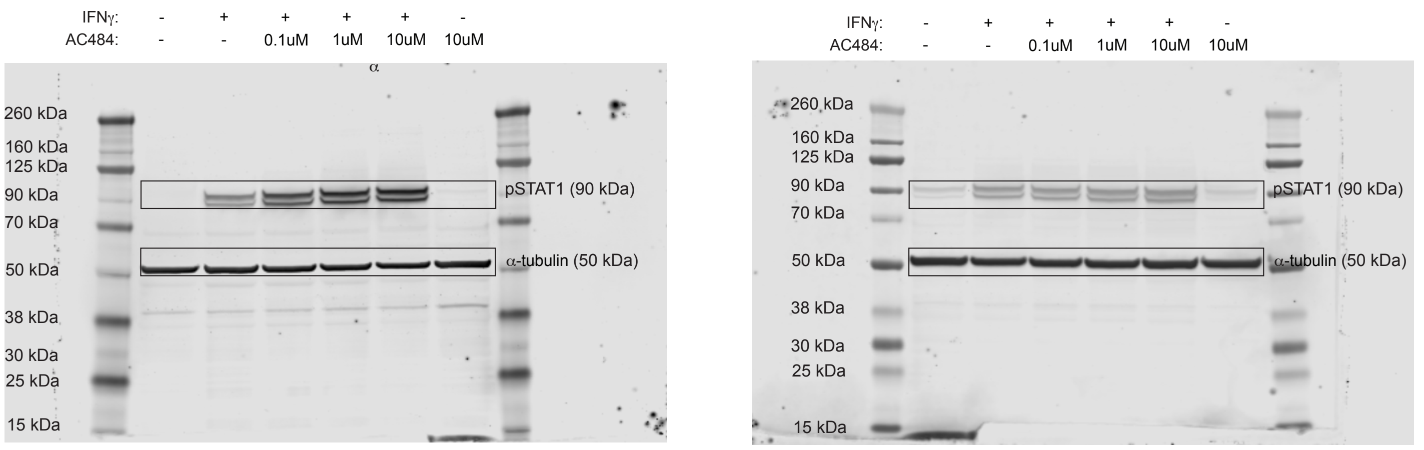

Extended Data Figure 1o

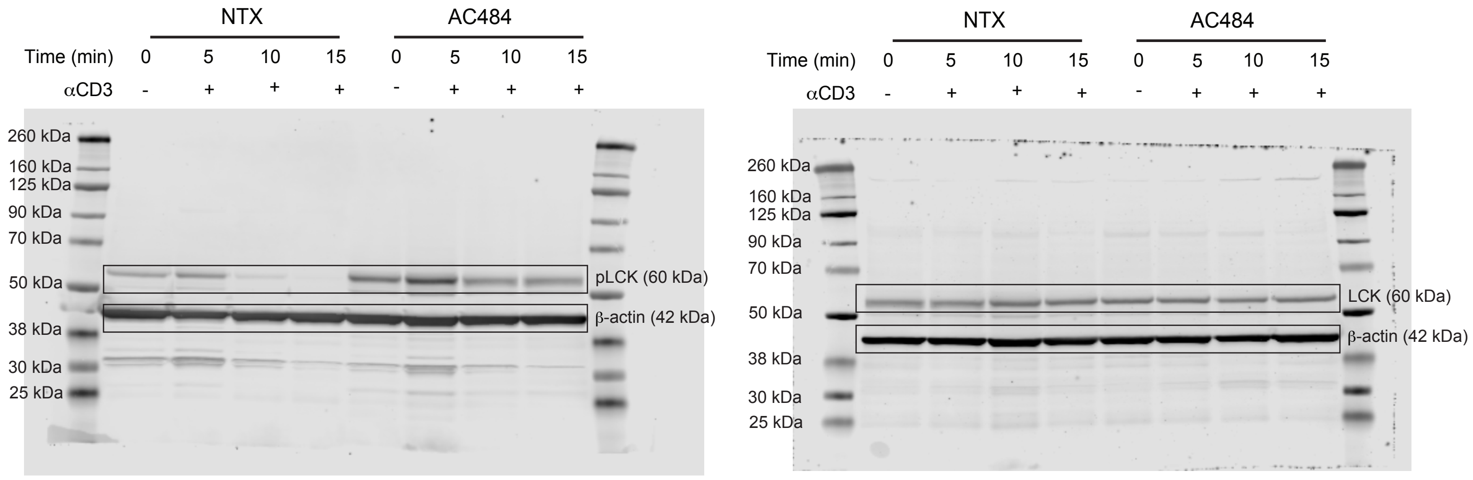

Extended Data Figure 1p

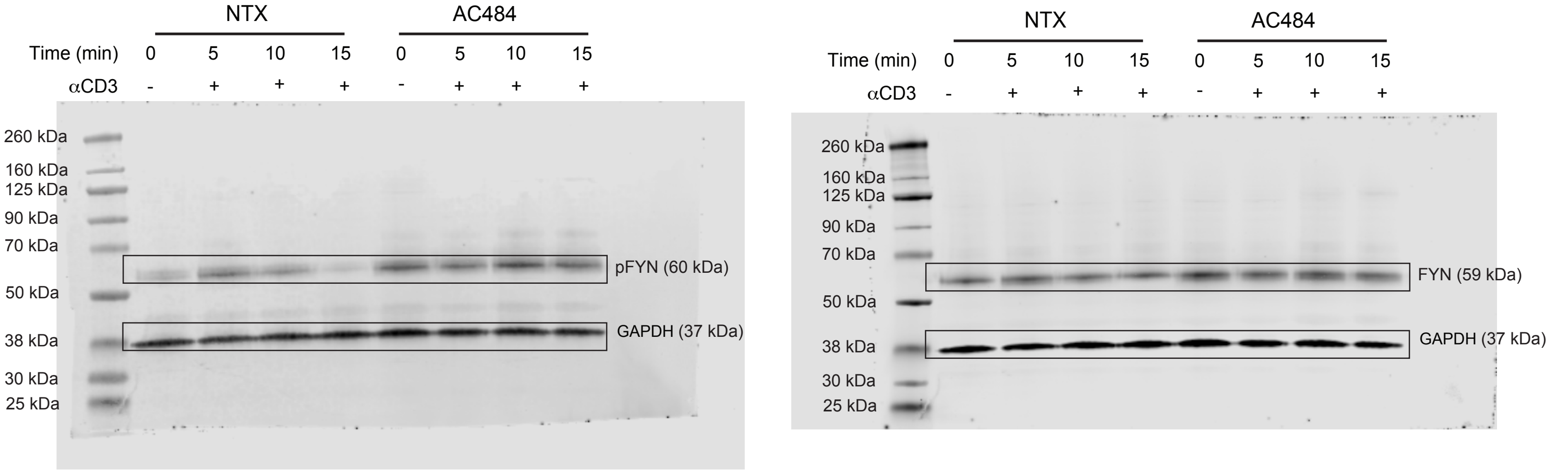

Extended Data Figure 8g

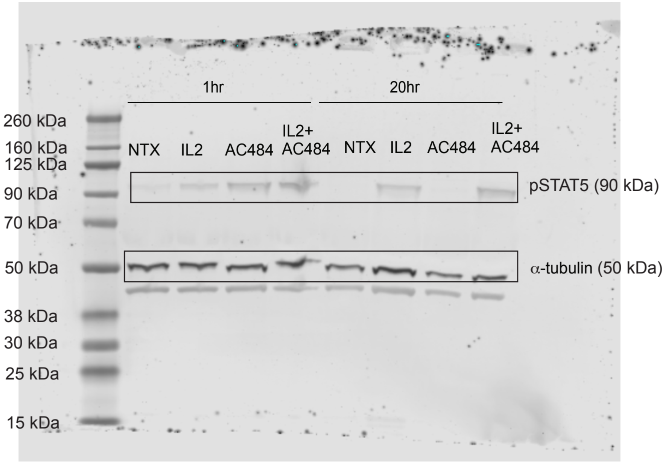

Extended Data Figure 8h

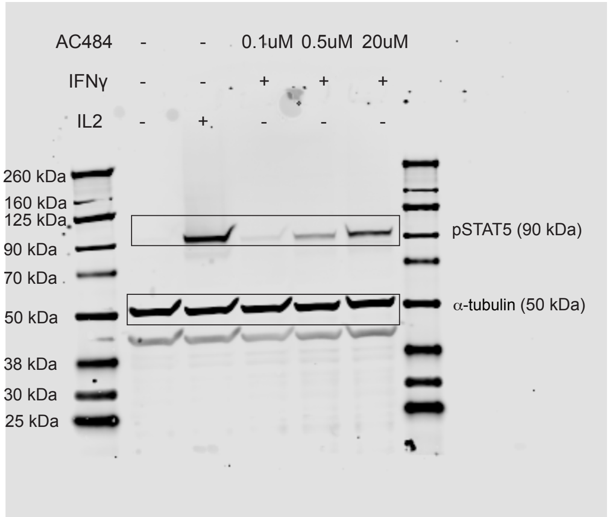

Supplementary Figure 1.

a. Gating strategy for NKCs, Foxp3^{+}/Foxp3^{-}, TOX/TIM3 and pSTAT5 in Figure 6a-b.

b. Gating strategy for NKCs, CD8 T cells and macrophages in Extended Data Figure 8b-e.

c. Full western blot scans with MW markers for Figure 6f, Extended Data Figure 1e, o, and p and Extended Data Figure 8g and h.
