# Supplementary material for: The PTPN2/PTPN1 inhibitor ABBV-CLS-484 unleashes potent anti-tumour immunity
Source: Nature. 2023 Oct 4;622(7984):850–62. doi: 10.1038/s41586-023-06575-7 (PMC10599993; doi:10.1038/s41586-023-06575-7)
Supplement: Supplementary file 2 — Reporting Summary [file 41586_2023_6575_MOESM2_ESM.pdf]

nature portfolio

Corresponding author(s): Christina K. Baumgartner, Jennifer M. Frost, Kathleen B. Yates, Philip R. Kym, Robert T. Manguso

Last updated by author(s): Jul 20, 2023

Reporting Summary

Nature Portfolio wishes to improve the reproducibility of the work that we publish. This form provides structure for consistency and transparency in reporting. For further information on Nature Portfolio policies, see our Editorial Policies and the Editorial Policy Checklist.

Statistics

For all statistical analyses, confirm that the following items are present in the figure legend, table legend, main text, or Methods section.

n/a	Confirmed	
☐	☒	The exact sample size (*n*) for each experimental group/condition, given as a discrete number and unit of measurement
☐	☒	A statement on whether measurements were taken from distinct samples or whether the same sample was measured repeatedly
☐	☒	The statistical test(s) used AND whether they are one- or two-sided<br>*Only common tests should be described solely by name; describe more complex techniques in the Methods section.*
☒	☐	A description of all covariates tested
☐	☒	A description of any assumptions or corrections, such as tests of normality and adjustment for multiple comparisons
☐	☒	A full description of the statistical parameters including central tendency (e.g. means) or other basic estimates (e.g. regression coefficient) AND variation (e.g. standard deviation) or associated estimates of uncertainty (e.g. confidence intervals)
☐	☒	For null hypothesis testing, the test statistic (e.g. *F*, *t*, *r*) with confidence intervals, effect sizes, degrees of freedom and *P* value noted<br>*Give P values as exact values whenever suitable.*
☒	☐	For Bayesian analysis, information on the choice of priors and Markov chain Monte Carlo settings
☒	☐	For hierarchical and complex designs, identification of the appropriate level for tests and full reporting of outcomes
☒	☐	Estimates of effect sizes (e.g. Cohen's *d*, Pearson's *r*), indicating how they were calculated

Our web collection on statistics for biologists contains articles on many of the points above.

Software and code

Policy information about availability of computer code

Data collection	Electrostatic calculation was done using Delphi (Open-Source from http://compbio.clemson.edu/delphi). Study Director (v4.5.0.0) was used for in vivo studies.
Data analysis	GraphPad Prism (8 and 9.1.0); Microsoft Excel (v2201 and v15); FlowJo (10.8.1); autoproc (1.1.7); PHASER (2.8.3); BUSTER (2.11.8); NanoStringNorm (1.2.1.1); Limma (3.46); Thermo Scientific Xcalibur Software (4.3); PEAKS Xpro (6.10); Python (3.7); DESeq2 (1.30.1); GSEA (4.1.0); cellranger (3.0.0); scanpy (1.7.2); MIXCR (3.0.13); Gliph2; bcl2fastq (v2.20.0); Trimmomatic (v0.36); FastQC (v0.11.7); Kallisto (v0.46.0); tximport (v1.24.0); Bowtie2 (v2.5.0); picard MarkDuplicates (v2.27.5); macs2 (v2.2.7.1); bedtools (v2.30.0); GREAT (v4.0.4); Integrative Genomics Viewer (v2.15.4); HOMER (v4.11.1); QuPath (0.3.0); R (v3.5.0); samtools (v1.6)

For manuscripts utilizing custom algorithms or software that are central to the research but not yet described in published literature, software must be made available to editors and reviewers. We strongly encourage code deposition in a community repository (e.g. GitHub). See the Nature Portfolio guidelines for submitting code & software for further information.

Data

Policy information about availability of data

All manuscripts must include a data availability statement. This statement should provide the following information, where applicable:

- Accession codes, unique identifiers, or web links for publicly available datasets
- A description of any restrictions on data availability
- For clinical datasets or third party data, please ensure that the statement adheres to our policy

Atomic coordinates and X-ray diffraction data were deposited to be available in the Protein Data Bank with the accession number 7UAD (activation in process, will

be available on July 26). Raw data for single-cell RNAseq, bulk transcriptomic, and ATAC-seq studies are available through the gene expression omnibus with accession number GSE237378. All other data used in the manuscript can be made available by the authors upon request.

Field-specific reporting

Please select the one below that is the best fit for your research. If you are not sure, read the appropriate sections before making your selection.

☒ Life sciences ☐ Behavioural & social sciences ☐ Ecological, evolutionary & environmental sciences

For a reference copy of the document with all sections, see nature.com/documents/nr-reporting-summary-flat.pdf

Life sciences study design

All studies must disclose on these points even when the disclosure is negative.

Sample size	Sample size of 5-10 mice/treatment group were used for in vivo studies as these group sizes were determined based on prior knowledge of typical biological variability with the tumor models and immunotherapy regimens employed and have historically been sufficient to provide statistical confidence in effects of immunotherapies on growth and survival effects (PMID: 32887697; 35256819, 31462409).
Data exclusions	Flow Cytometry data files were excluded when total cellularity, that is gated live, single cells, was less than 1,000 events. Furthermore, calculated subsets were omitted when the number of events in key lineages dropped below 500 events. This criterion was applied to the Total T cell gate in the T cell panel, and the Macrophage gate in the Myeloid Panel. Data from whole blood showing occasional overt clotting after overnight culture were excluded
Replication	All results presented in the manuscript were replicated at least twice in independent experiments.
Randomization	Within each in vivo experiment, mice were randomized into treatment conditions based on similar distribution of tumor volumes at the indicated time that treatments began or by cage following tumor innoculation. Randomization was not applicable for in vitro studies.
Blinding	Researchers were not blinded to treatment groups for in vivo or in vitro studies because knowledge of this information was essential to conduct the studies.

Reporting for specific materials, systems and methods

We require information from authors about some types of materials, experimental systems and methods used in many studies. Here, indicate whether each material, system or method listed is relevant to your study. If you are not sure if a list item applies to your research, read the appropriate section before selecting a response.

Materials & experimental systems

n/a	Involved in the study	
☐	☒	Antibodies
☐	☒	Eukaryotic cell lines
☒	☐	Palaeontology and archaeology
☐	☒	Animals and other organisms
☐	☒	Human research participants
☒	☐	Clinical data
☒	☐	Dual use research of concern

Methods

n/a	Involved in the study	
☒	☐	ChIP-seq
☐	☒	Flow cytometry
☒	☐	MRI-based neuroimaging

Antibodies

Antibodies used

Anti-mouse PD-1 antibody (17D2[mu IgG2a/κ] DANA) for in vivo blockade PD-1 and anti-mouse PD1 antibody (clone 29F.1A12, BioXcell) for in vivo blockade of PD-1 in the KPC model; For in vivo depletion experiments, anti-CD8b (Bio X Cell, clone 53-5.8), anti-NK1.1 (Bio X Cell, clone PK136, monoclonal), and IgG2a isotype control (Bio X Cell, clone C1.18.4, monoclonal) were used.

For Western blot assays, the following primary antibodies were used to detect designated protein expression: Anti-TCPTP (Abcam, ab180764), STAT1 (Cell Signaling, 9172), phospho-STAT1 (Cell Signaling, 9167), STAT5 (Cell Signaling, 94205), phospho-STAT5 (Cell Signaling, 9359), LCK (Invitrogen, AHO0472), phospho-LCK (Cell Signaling, 70926), phospho-SRC family kinase (Cell Signaling, 6943), FYN (Cell Signaling, 4023).

For T cell repetitive stimulation assay Human T-activator CD3/28 Dynabeads containing anti-CD3 and anti-CD28 antibodies (Thermo Fisher Cat# 11132D) was used.

For Flow Cytometry, the following anti-mouse fluorochrome-conjugated antibodies were used: CD8α (clone 53-6.7, BioLegend or BD Biosciences), CD4 (clone RM4-5 or GK1.5, BioLegend), TCRβ (clone H57-597, BioLegend), Tim-3 (clone 5D12, BD Biosciences or clone RMT3-23, BioLegend), Lag-3 (clone C9B7W, BD Biosciences), CD45 (clone 30-F11, ThermoFisher) CD45.2 (clone 104, BioLegend),

	NK1.1 (clone 108741, BioLegend), CD44 (clone 103028 or IM7, BioLegend), FOXP3 (clone JFK-16s, eBioscience), Granzyme B (clone GB11, BioLegend), Tox (clone TXRX10, eBioscience), Perforin (clone S16009B, BioLegend), PD-L1 (clone 10F.9G2, BioLegend), MHC-I (clone 28-8-6, BioLegend), MitoTracker™ Deep Red FM (ThermoFisher Scientific), CD278 (clone C3978.4A, BD Biosciences), CD27 (clone LG.3A10, BD Biosciences), KLRG1 (clone 2F1, BD Biosciences), CD69 (clone H1.2F3, BD Biosciences), Slamf6 (clone 13G3, BD Biosciences), PD-1 (clone 29F.1A12, BioLegend) Ki-67 (clone B56, BD Biosciences), CD62L (clone MEL-14, BioLegend), CTLA-4 (clone UC10-4F10-11, BD Biosciences), TCF7 (clone 2203, Cell Signaling), TNFα (clone MP6-XT22, BioLegend), CD3 (clone 17A2, BioLegend), IFNγ (clone XMG1.2, BioLegend), STAT5 (Cell Signaling, 94205), phospho-STAT5 (Cell Signaling, 9359), Anti-rabbit IgG (H+L), F(ab')2 Fragment (Cell Signaling, 4412).
Validation	Validation of the specificity of this antibody for mouse PD-1 and the effect of the Fc DANA (D265A-N297A) mutation to prevent binding to FcγR has been demonstrated previously (PMID: 35256819). Validation of the BioXcell antibodies available on the manufacturer's website: https://www.bxcell.com. Validation of antibodies is provided on the manufacturers websites: https://www.biolegend.com/, https://www.bdbiosciences.com/, https://www.thermofisher.com/, https://www.novusbio.com/, https://www.cellsignal.com/, https://bxcell.com.

Eukaryotic cell lines

Policy information about cell lines

Cell line source(s)	B16 and B16-GM-CSF (GVAX) lines were received as a gift from G. Dranoff (Dana-Farber Cancer Institute). The KPC pancreatic cancer cell line was a gift from A. Maitra and S. Dougan. MC38 colon carcinoma cell lines were obtained from the National Cancer Institute or as a gift from A. Sharpe. HT-29 colon carcinoma, A375, CT26.WT colon carcinoma (referred to as CT26), EMT-6 and 4T1 cells were purchased from ATCC. Nuclight Red expressing B16-OVA cells were generated at AbbVie using Incucyte Nuclight Red Lentivirus (Sartorius) .
Authentication	The parental cell lines were authenticated by ATCC and NCI
Mycoplasma contamination	All cell lines were tested for Mycoplasma and maintained in culture for no more than 16 passages from master cell banking. Cell lines were consistently maintained in a prophylactic dose of plasmocin and periodically tested for Mycoplasma with negative results.
Commonly misidentified lines (See ICLAC register)	No misidentified cell lines were used in this study.

Animals and other organisms

Policy information about studies involving animals; ARRIVE guidelines recommended for reporting animal research

Laboratory animals	Female C57BL/6, BALB/c and NOD.Cg-Prkdcscid Il2rgtm1Wjl/SzJ (NSG) mice and male NSG mice ages 6-12 weeks
Wild animals	No wild animals were utilized
Field-collected samples	No Field-collected samples were utlized
Ethics oversight	IACUC committee of AbbVie; IACUC committee of the Broad Institute of MIT and Harvard.

Note that full information on the approval of the study protocol must also be provided in the manuscript.

Human research participants

Policy information about studies involving human research participants

Population characteristics	PBMCs/ whole blood from healthy volunteers was used for in vitro immune cell activation studies. At AbbVie, PBMCs/ whole blood from cancer patients was acquired from Discovery Life Sciences and used for in vitro immune cell activation studies. At Broad, human T cells were from STEMCELL Tech from consented donors.
Recruitment	AbbVie employee volunteers who signed informed consent; Cancer patient's blood from Discovery Life Sciences
Ethics oversight	Human blood samples were acquired through the internal AbbVie Inc's blood donation program in accordance with AbbVie's Occupational Safety and Health Administration protocols. The protocol, under which human blood samples were acquired, was approved by and is reviewed on an annual basis by WCG IRB (Puyallup, Washington).

Note that full information on the approval of the study protocol must also be provided in the manuscript.

Flow Cytometry

Plots

Confirm that:

☒ The axis labels state the marker and fluorochrome used (e.g. CD4-FITC).

☒ The axis scales are clearly visible. Include numbers along axes only for bottom left plot of group (a 'group' is an analysis of identical markers).

☒ All plots are contour plots with outliers or pseudocolor plots.

☒ A numerical value for number of cells or percentage (with statistics) is provided.

Methodology

Sample preparation	In vivo mouse tumor models – Resected tumors were processed using the Miltenyi Biotec mouse tumor dissociation enzymatic kit along with the GentleMACS dissociator and associated '37C-m-TDK' program. Spleens and Lymph Nodes were processed in RPMI using the GentleMACS dissociator and associated 'm-spleen' program. 100uL whole blood was prepared in an equal volume of EDTA to prevent coagulation. Splenocytes were RBC lysed with 2mL of 1X RBC lysis buffer (Thermo-Fisher) for 4 minutes at RT, and blood was lysed with 300uL ACK lysis buffer (Thermo-Fisher) for 2 minutes at RT and immediately quenched with MACS Buffer (Miltenyi Biotec). Washing steps for tumor, spleen and blood were done with MACS Buffer and centrifuged at 300xg for 5 minutes. Lymph nodes were centrifuged for 10' at 400xg. Tumor and splenocytes were filtered with a 40um mesh filter prior to counting and staining. Tumor and spleen samples were counted, and up to 2 million live cells were stained for each sample. Blood and lymph node samples were split evenly among the two panels typically run. PBS-washed and pelleted cells were first stained with 50uL of fixable live/dead viability dye and then with 50uL of a cocktail of surface antibodies prepared at the proper dilution in Superblock blocking buffer (2% FCS, 5% normal rat serum, 5% normal mouse serum, 5% normal rabbit serum, 10ug/mL 2.4G2, PBS). Samples were stained for 30', washed twice, and fixed and permeabilized using the FoxP3 Transcription Factor Buffer set (Thermo-Fisher) according to the manufacturers protocol. Intracellular antibodies were cocktailed in Perm/Wash buffer at the appropriate dilution and samples were stained with 100uL at 4C overnight. Samples were finally washed in Perm/Wash twice, resuspended in MACS buffer, and passed through a final 30um mesh plate filter (Millipore) prior to acquisition on the Aurora Spectral Analyzer (Cytek Biosciences).<br>For a subset of experiments at the Broad, tumors were chopped before chemical and mechanical digestion with the Tumour Dissociation Kit (Miltenyi) and the gentleMACS Dissociator (Miltenyi) using the m-TDK-1 program. The resulting cell suspension was passed through a 70-µM filter. Isolation of immune cells was performed by density centrifugation with lympholyte reagent (Cedarlane labs) followed by positive selection for CD45+ cells with MicroBeads and magnetic separator (Miltenyi). Cells were blocked with TruStain FcX Plus (anti-mouse CD16/32) antibody (1:100, BioLegend) in PBS + 2% FBS. Dead cells were excluded using Live/Dead™ Fixable Blue Dead Cell Stain (1:1,000, ThermoFisher) added concurrently with surface antibodies. Samples were stained with indicated antibodies for 30 min on ice. After washing, cells were fixed with Foxp3/Transcription Factor Staining Buffer Set (eBiosciences) as per manufacturer's instructions, blocked with TruStain FcX, mouse and rat serum, then stained with intracellular antibodies. For pSTAT5 staining, cells were fixed in 4% PFA for 15 minutes at 37C and then permeabilized with 90% methanol for 20 minutes on ice before proceeding with blocking and staining for intracellular and extracellular markers. Samples at the Broad were analyzed on a Beckman Coulter Cytoflex LX.
Instrument	Aurora Spectral Analyzer (Cytek Biosciences), Fortessa X20 (BD Biosciences), NovoCyte Quanteon (Agilent), Cytoflex LX (Beckman Coulter)
Software	FlowJo v.10.8.1 (BD Biosciences), OMIQ (Insightful Science).
Cell population abundance	N/A
Gating strategy	For all flow cytometry experiments, gates were drawn based on single-stain and full-minus-one (FMO) controls and unstained samples.<br>In vivo mouse tumor models: Expert manual gating was done by a single analyst to minimize variance. Pre-processed FCS files were gated on live cells first using FSC vs. Viability Dye parameters making sure to include all live subsets from lymphocytes to tumor cells. CD45positive and negative cells were gated based on CD45 fluorescence and SSC signals. A region was easily drawn between the bimodal distribution to separate positive and negative fractions. CD45pos cells were further gated on single cells using FSC-H and FSC-A signals and drawing a region around events that fall along a correlation line. Downstream subsets were gated in a way to isolate major lineages (T cells, Myeloid cells, etc...) and then further gating each major lineage into specific populations. Finally, expression markers (e.g. ICOS, Granzyme B, iNOS) were gated based on unstained samples and/or fluorescence minus one controls, as needed. An example gating figure is provided in the Supplement.

☒ Tick this box to confirm that a figure exemplifying the gating strategy is provided in the Supplementary Information.
